# Is Gen AI an editor or a revision tool? A case study of EFL students in AI-assisted revision

**DOI:** 10.1371/journal.pone.0359661

**Published:** 2026-10-29

**Authors:** Han Zhang, Shanshan Ai, Lingyu Zhang, Yuhui Lin

**Affiliations:** 1 Hebei Normal University, Shijiazhuang, Hebei, China; 2 University of Malaya, Kuala Lumpur, Malaysia; 3 Hebei Minzu Normal University, Chengde, Hebei, China; Kalasalingam Academy of Research and Education, INDIA

## Abstract

As generative artificial intelligence (Gen AI) tools become increasingly integrated into language education, understanding how learners respond to AI-generated feedback during writing revision has become an important research concern. Existing studies have primarily examined the effects of AI on writing performance, with less attention to learners’ reported decision-making and reflection during revision. This qualitative study investigated learner-AI interaction, reported revision practices and learner perceptions among 18 first-year non-English-major students enrolled in a university EFL writing course. Data were collected through semi-structured interviews following a Gen AI-assisted revision task and analysed using thematic analysis. The findings identified three interaction patterns with Gen AI: passive AI reliance, adaptive AI engagement, metacognitive AI engagement. Participants differed in how they evaluated and used AI-generated feedback, including passive acceptance, selective uptake, and critical evaluation. Some reported increased confidence, motivation, and awareness of linguistic form, whereas others expressed concerns about overreliance, authorship, and loss of personal voice. These self-reported experiences provide insight into how EFL learners perceive and manage GenAI-assisted revision, highlighting the need for pedagogical approaches that foster critical AI literacy and reflective learner autonomy.

## Introduction

In L2 writing research, revision is widely recognised as a key process for language development and writing proficiency [1,2]. In contrast to first draft production, revision involves not only the correction of surface-level linguistic errors but also cognitive monitoring, evaluation and reconstruction, thereby linking language production with metacognitive development [3]. Particularly in English as a Foreign Language (EFL) contexts, students often lack sufficient linguistic confidence and revision strategies. As a result, their revision practices tend to focus on surface-level corrections rather than deeper restructuring of ideas and arguments.

With the rapid development of Generative Artificial Intelligence (Gen AI), particularly large language models such as ChatGPT, AI-assisted writing support has become increasingly prominent in educational settings. The adoption of these technologies is widely viewed as both inevitable and transformative for writing instruction [4,5]. GenAI systems can provide immediate feedback, linguistic refinement and structural suggestions, which may help learners improve the quality of their written output during revision [6]. Previous studies have shown that AI-assisted tools can support EFL students in conducting multiple rounds of revision involving grammar, vocabulary, and textual coherence, and in some cases, may partially replicate feedback functions traditionally performed by teachers [7,8].

Despite these promising developments, the integration of GenAI into writing practices has also raised important pedagogical concerns. Researchers have warned that excessive reliance on AI-generated feedback may weaken learners’ autonomy, writing identity, and critical engagement with their own texts [9,10]. Some studies report that students tend to accept AI suggestions mechanically without evaluating or questioning the proposed revisions, which may limit the development of independent writing skills over time [11].

Although an emerging body of research has examined metacognition, learner agency, and human–AI collaboration in AI-assisted writing, important questions remain regarding how these dimensions intersect during revision. Existing studies have shown that learners may monitor, evaluate, accept, reject, or negotiate AI-generated feedback rather than simply follow it. However, less is known about how EFL learners perceive the role of GenAI during revision, how these perceptions relate to their reported revision behaviours, and how they shape learners’ attitudes toward writing and learning. This study addresses this gap by providing a qualitative account of learners’ self-reported experiences following a GenAI-assisted revision task.

## Literature review

### Definition of editing and revision

In L2 writing research, editing is typically understood as the process through which learners identify and resolve linguistic problems in their drafts, including errors in grammar, vocabulary, mechanics, and clarity [3,12]. Although often associated with surface-level correction, studies have shown that editing in a second language can also involve higher-level cognitive operations such as noticing, evaluation and self-monitoring [13]. Schmidt proposed that when L2 learners detect discrepancies between their intended meaning and the language they produce, this awareness can trigger restructuring of their interlanguage system [14]. Such noticing may occur through self-editing or external feedback from peers, teachers or automated tools.

Editing is embedded within the broader construct of revision in L2 writing. Revision involves both surface-level changes, such as adjustments to vocabulary, grammar, and sentence structure, and meaning-oriented changes, including reorganising ideas, refining argumentation, and improving rhetorical coherence. L2 writing scholars describe revision as a recursive and reflective process in which writers reread, reconsider intentions, and refine their writing to better meet communicative goals [12,15]. In this sense, editing provides a linguistic foundation for revision, while revision offers the cognitive space for editing to contribute to deeper textual improvement.

Taken together, these perspectives highlight that editing and revision involve both linguistic correction and cognitive engagement. However, in EFL contexts, these processes are increasingly mediated by AI tools. While GenAI systems can efficiently provide surface-level corrections, such as grammar adjustments or stylistic refinement, learners may accept suggestions uncritically, limiting opportunities for metacognitive engagement. Scaffolding, such as think-aloud protocols or guided rewriting, may therefore be needed to encourage learners attend to linguistic form and evaluate the appropriateness of revisions [6]. Building on this literature, the present study conceptualises AI-assisted editing as situated within the broader revision process and examines how EFL learners attend to, evaluate and integrate AI-generated feedback during revision.

### From AWE to Gen AI writing revision

Automated Writing Evaluation (AWE), a form of machine scoring, has been widely used to support writing [13]. AWE systems are designed to recognise certain types of linguistic errors and provide automated feedback to help learners revise their texts [16]. Research indicates that AWE can support both editing and certain aspects of revision. For example, platforms such as Grammarly and Pigai website have been shown to improve learners’ writing performance by identifying grammatical errors and offering corrective suggestions [17,18]. In combination with teacher feedback, AWE may also support early drafting and surface-level editing. For instance, students who received both AWE feedback and instructor rubrics outperformed those who received instructor feedback alone, indicating that automated feedback can scaffold the initial stages of revision by alerting learners to linguistic or structural weaknesses [19].

However, the effectiveness of AWE is not consistently supported across contexts. For example, automated feedback did not reliably improve the final drafts of lower-proficiency EFL learners in China, suggesting that such tools may be less effective when students lack the metacognitive skills required to interpret feedback [20]. Similarly, AWE contributes little to writing development unless learners actively engage with its suggestions [21]. Although AWE may facilitate early drafting and surface-level revision [22], learner uptake often remains limited. Its pedagogical impact is also constrained by algorithmic opacity and limited transparency in analytic processes [23]. Thus, these findings suggest that the effectiveness of automated feedback depends largely on learners’ ability to interpret and meaningfully engage with the suggestions provided.

In response to these limitations, recent studies have increasingly turn to Gen AI tools such as ChatGPT, DeepSeek, and Doubao. Unlike traditional AWE systems, which primarily detect errors, GenAI can provide more flexible and context-sensitive support by suggesting alternative expressions, rephrasing meaning, reorganising ideas, and offering rhetorical options [24]. This development reflect a shift towards interactive computational systems capable of generating meaningful text through human–machine dialogue [25]. In educational settings, GenAI has been found to support personalised feedback, iterative revision and greater learner agency [26]. These capabilities align more closely with contemporary models of writing revision as recursive and meaning-oriented processes involving reflection, restructuring and negotiation of communicative goals [15].

Despite these possibilities, important challenges remain in the pedagogical use of GenAI. Although these systems can respond to prompts and assist with drafting [27,28], learners must critically evaluate the accuracy, appropriateness, and rhetorical suitability of AI-generated output [29]. For example, GenAI lacks genuine understanding and may produce superficial reasoning or biased content, making it difficult for learners to distinguish between meaningful revision and automated paraphrasing [30]. In addition, excessive reliance on GenAI may weaken learners’ development of higher-order thinking skills, including critical thinking, problem-solving, creativity and independent reasoning [30,31]. These concerns relate to broader debates about the educational use of Gen AI, including learner autonomy, plagiarism, data privacy and algorithmic bias [25,32]. When learners accept AI-generated suggestions without critical evaluation, opportunities for reflective revision and cognitive engagement may be reduced. These concerns highlight the importance of examining how learners interact with AI feedback during the revision process.

Taken together, these studies indicate that both AWE and GenAI can support editing and revision, yet their pedagogical impact ultimately depends on how learners engage with these tools. While GenAI offers more interactive and context-sensitive support than earlier automated systems, it also raises questions about learners’ engagement with AI-generated feedback. Understanding how learners interpret, evaluate and integrate such feedback therefore remains an important area for investigation.

### Gen AI-assisted writing revision in EFL context

Recent studies highlight that GenAI tools provide increasingly sophisticated support for editing and revision in EFL writing [33]. Compared with earlier automated systems, Gen AI can generate context-sensitive feedback, offer alternative expressions and assist learners in refining both linguistic accuracy and textual organisation. When learners submit their drafts to systems such as ChatGPT, they may receive personalised feedback that targets vocabulary, grammar and rhetorical clarity [34–36]. In this sense, GenAI functions not only as a linguistic editor but also a revision-oriented guide capable of suggesting improvements in content development and organisation [7,8]. Empirical studies have reported improvements across multiple dimensions of writing, including grammar, vocabulary, organization and content quality [6,37]. These findings indicate that GenAI has the potential to support both surface-level editing and meaning-level revision in EFL writing [10,38].

Importantly, recent work highlights that learners’ interaction with GenAI is shaped by affective and motivational factors. Studies show that AI literacy, technical familiarity and enjoyment significantly influence how learners engage with AI-mediated communication [39]. Similarly, AI-enhanced language learning can activate distinct emotional and autonomy-related learning profiles, suggesting that students’ engagement with GenAI is influenced not only by cognitive processes but also by affective experiences and perceptions of control. These findings highlight the multifaceted of nature of learners’ responses to GenAI-assisted editing, involving cognitive, emotional and motivational dimensions [40,41].

Despite these advantages, a growing body of research identifies important limitations in the pedagogical use of GenAI for writing revision. Scholars have raised concerns that learners may rely excessively on AI-generated suggestions, accepting revisions without critical evaluation and thereby reducing opportunities for independent editing and higher-order revision [6,10]. In some cases, students may bypass essential revision processes by directly incorporating AI-generated text into their drafts. Academic integrity issues may also arise when AI-produced content is submitted without sufficient learner contribution [9]. Moreover, studies suggest that GenAI feedback may still focus primarily on surface-level corrections rather than supporting deeper rhetorical development such as argumentation, coherence, and idea elaboration [11]. These findings indicate that the effectiveness of GenAI-assisted revision depends not only on the availability of automated feedback but also on how learners interpret, evaluate, and apply such feedback during the revision process.

These concerns are closely related to long-standing theories of revision in second language writing. Research on revision processes highlights the importance of noticing and reformulation in promoting language development [1]. Reformulation involves producing an improved version of a learner’s text while preserving meaning, while noticing refers to the learner’s conscious comparison between the original and reformulated versions. Through this comparison process, learners may become aware of linguistic and rhetorical differences, facilitating language awareness and improve both accuracy and fluency [2]. However, noticing alone does not determine revision decisions. Learners must evaluate AI-generated suggestions and decide whether to accept, reject, modify, or further question them, a process involving metacognitive regulation and writing agency. In this study, writing agency refers to learners’ capacity to retain intentional control over revision decisions in accordance with their intended meaning, personal voice, and writing goals. From this perspective, GenAI-generated revisions may function as reformulations that prompt noticing, while learners’ subsequent responses reflect metacognitive regulation and writing agency. Human–AI interaction therefore refers to the process through which learners interpret, evaluate, accept, reject, or modify AI-generated feedback during writing revision.

Taken together, existing research suggests that GenAI can provide valuable support for writing revision and may influence both linguistic development and learner motivation. However, much of the current literature focuses on writing outcomes, such as improvements in accuracy, organisation or overall text quality. Comparatively less attention has been paid to how learners cognitively engage with AI-generated feedback during revision. Qualitative evidence remains limited regarding how learners notice, evaluate, and integrate AI suggestions while revising their drafts. Addressing this gap is important for understanding the pedagogical implications of GenAI-assisted writing. Therefore, the present study investigates how EFL learners interact with GenAI during revision and how such interaction relates to their revision strategies, language awareness, and perceptions of learning. Accordingly, these concepts informed the formulation of the research questions:

RQ1: How do EFL learners perceive their interaction with Gen AI during writing revision?
RQ2: In what ways do learners engage in metacognitive activities during Gen AI-assisted writing revision?
RQ3: What are EFL students’ perceptions of Gen AI-assisted revision and its influence on English learning?

## Methodology

This study adopts a qualitative research design to investigate how EFL learners cognitively engage with Gen AI-assisted editing during revision. Situated within an interpretivist paradigm, the study sought to understand learners’ reported revision behaviours, noticing patterns, and reflective practices in response to AI-generated feedback.

### Participants

Participants were recruited from a first-year compulsory English course at University A, a public university in China. The class was purposively selected because the English instructor regularly incorporated GenAI-assisted writing revision into classroom activities, providing an appropriate context for investigating learners’ engagement with AI-assisted revision. The class consisted of 50 non-English majors, all of whom participated in the revision task.

Following the task, students’ revised drafts and AI interaction records were preliminarily reviewed and grouped into three broad patterns of AI engagement. This preliminary categorisation was used for purposive sampling rather than as the final analytical framework. Participants representing these patterns were then purposively invited for semi-structured interviews. Interviews continued until no substantially new information emerged, resulting in a final interview sample of 18 students (10 females and 8 males). Their English proficiency was benchmarked using Gaokao English scores, with an average of 103 (range = 82–127 out of 150), indicating an intermediate proficiency level.

### Procedure

The writing task was embedded within a unit on comparison-based essays in a compulsory English writing course for first-year non-English majors. The unit was based on *New Horizon College English (Fourth Edition)* and included a model essay illustrating the organisation and linguistic features of comparison-based writing. After analysing the model essay, students were asked to write an essay on “Differences between the younger generation and their parents’ generation”, following the linguistic features. The original task instructions were provided in Chinese to ensure accurate understanding, with an English translation provided for international readers (Fig 1).

**Fig 1:**
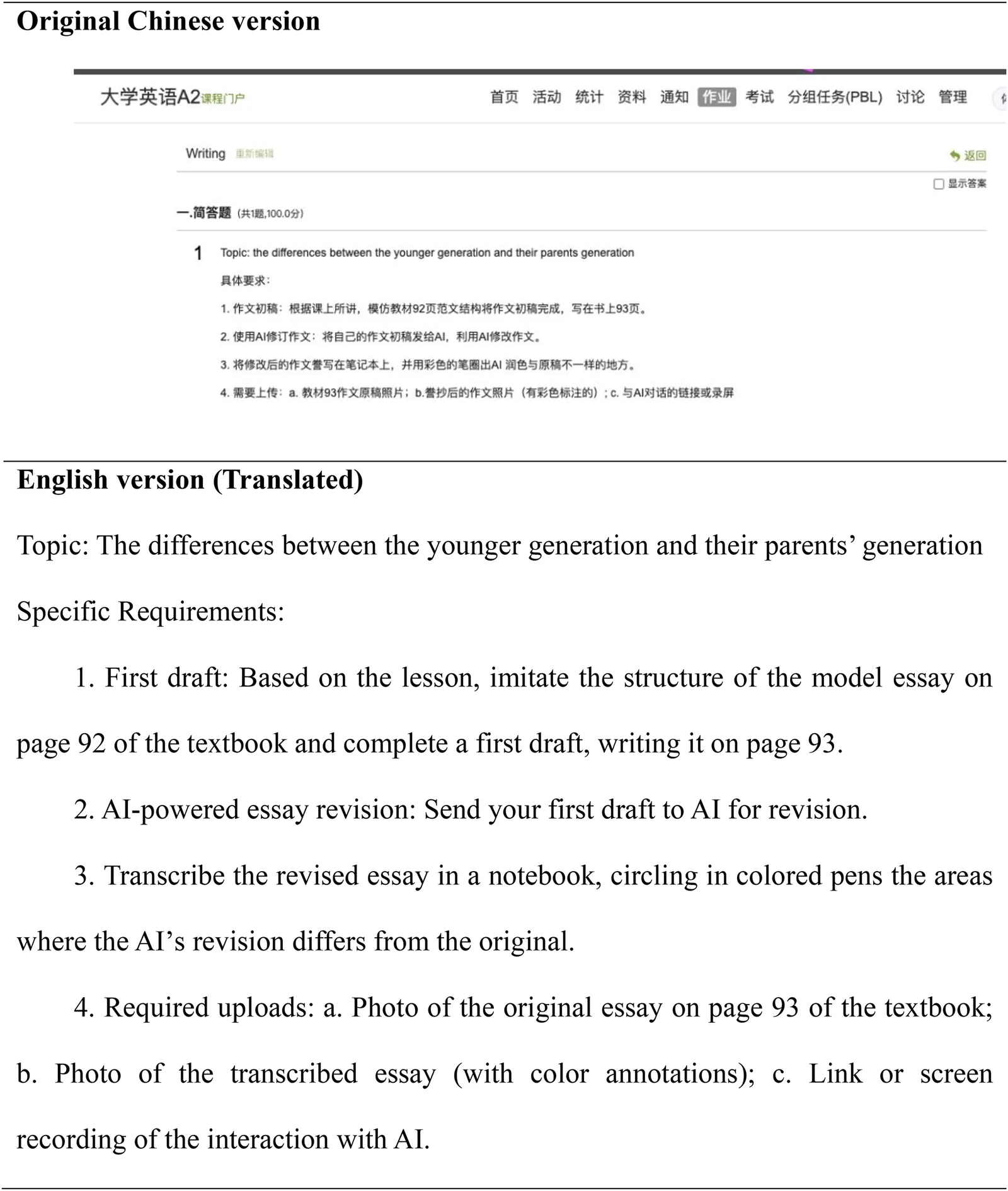
Writing task.

All 50 students completed their first drafts without AI assistance and then used GenAI to obtain feedback and revise their writing. They submitted their drafts by uploading or manually entering the text and could use commonly available GenAI tools, including Doubao, Kimi, and DeepSeek, according to their preferences. No specific platform or prompt template was prescribed, allowing variation in how students independently interacted with GenAI. This included prompt adjustment, questioning, and acceptance or rejection of AI suggestions. Although this reduced direct comparability across participants, such variation was analytically relevant to exploring different patterns of engagement.

As part of the course assignment, students transcribed the AI-revised version and marked differences from their original drafts using coloured pens. This comparison activity was intended to make AI-related changes visible and encourage students to consider whether the revisions aligned with their intended meanings. The original drafts, annotated revised versions, and AI interaction records were subsequently collected. These materials were preliminarily reviewed and grouped into three broad patterns of AI engagement to guide purposive selection for follow-up interviews. This preliminary categorisation served as a sampling strategy rather than the final analytical framework. Students representing the different patterns were invited for interviews, which continued until no substantially new information emerged.

### Data collection

Three sources of data were collected: students’ original and AI-revised drafts, records of their interactions with Gen AI, and semi-structured interviews. As described in the Procedure section, the drafts and AI interaction records were used to support preliminary categorisation and participant selection and to provide contextual information for the subsequent interviews. The semi-structured interviews constituted the primary qualitative dataset for thematic analysis.

Eighteen students participated in the interviews, which were conducted in Mandarin between June and July 2025, audio-recorded with participants’ consent, and transcribed verbatim. The interview protocol (Appendix in S1 Appendix) was informed by the concepts of human–AI interaction, reformulation, noticing, and writing agency. It explored four areas: (1) prior experience with AI tools, (2) use of GenAI during writing revision, (3) decision-making and metacognitive engagement when responding to AI feedback, and (4) perceptions of the influence of GenAI on writing and English learning.

**Fig 2:**
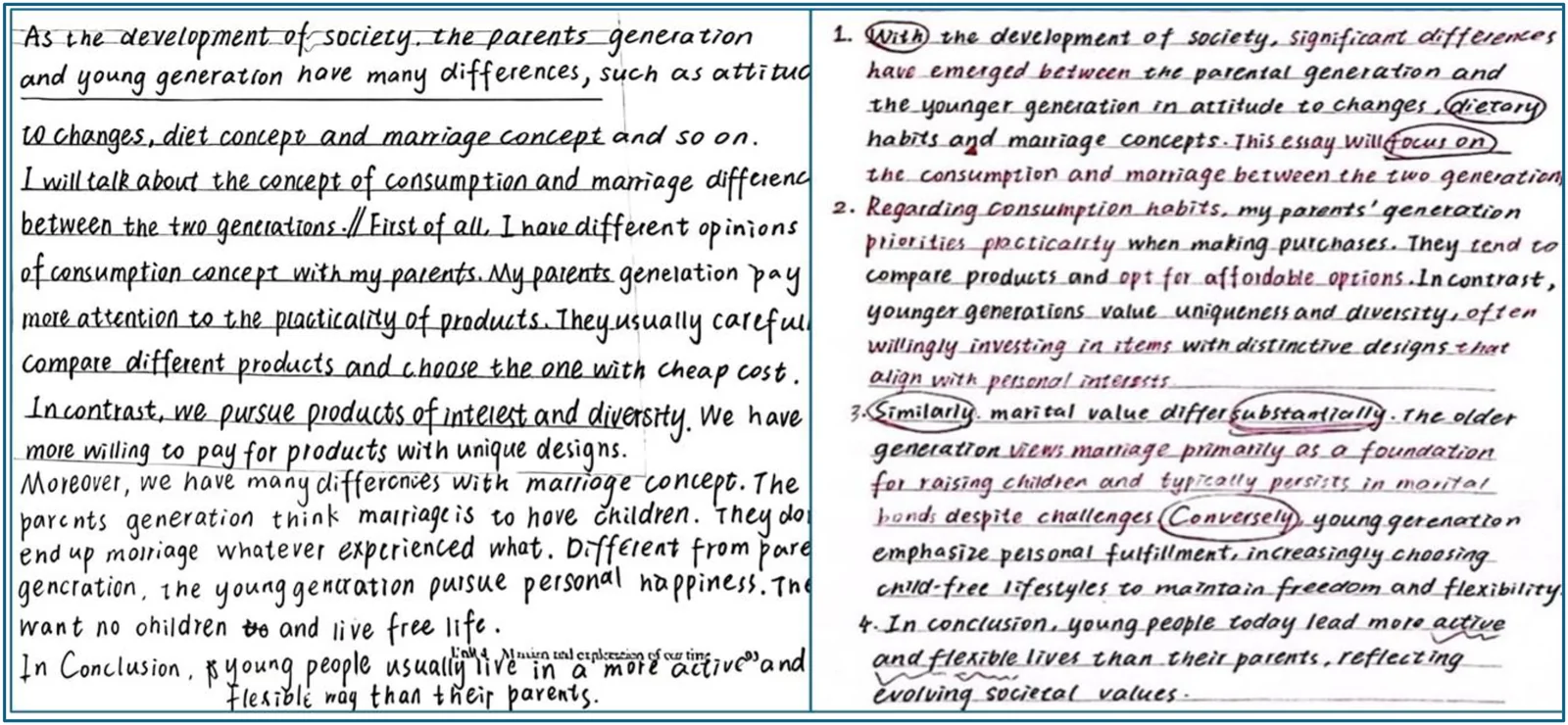
An example of a student who selectively adopted AI suggestions.

### Data analysis

Interview transcripts were analysed using a combined deductive–inductive thematic analysis. Deductive coding was informed by the research questions and sensitising concepts of human–AI interaction, reformulation and noticing, while inductive coding remained open to themes emerging from participants’ accounts. In this study, human–AI interaction refers to the process through which learners interpreted, evaluated, accepted, rejected or modified AI-generated feedback during writing revision. Rather than serving as fixed analytical categories, reformulation and noticing functioned as sensitising concepts that informed the interview protocol, initial coding, and interpretation of learners’ revision experiences. Writing agency informed the deductive coding of participants’ decision-making, including selective acceptance, informed rejection, prompt adjustment, modification of AI output, and efforts to retain authorial voice and control over the final text.

The coding procedure included three stages [42]. First, open coding was conducted line by line to identify meaningful units related to learners’ experiences of AI-assisted revision. At this stage, both theory-informed codes and data-driven codes were generated. Next, axial coding was used to compare and group related codes into broader different patterns of learner engagement. Finally, selective coding was employed to integrate these patterns into higher-order themes that addressed the three research questions.

To ensure analytic transparency, two researchers independently coded 20% of the data. The Cohen’s kapa coefficient was 0.81, with discrepancies discussed and resolved through consensus. A revised codebook was then applied to the full dataset by the lead coder.

### Ethic concern

This study adheres to the University’s ethical guidelines. Prior to data collection, participants were fully informed of the study’s aims and scope, and written consent was obtained. Participation was entirely voluntary, with no compensation offered, and participants were informed of their right to withdraw at any point without penalty. They could also request the deletion of their data at any time. Participation had no bearing on their academic achievements.

## Result

The analysis identified specific themes and dimensions corresponding to each research question (see Tables 1–3).

**Table 1:** Core themes, themes and coding derived from the qualitative data for RQ1.

Core themes	Themes	Coding
Passive AI reliance	Uncritical acceptance	Blind acceptance of AI revisions
		Perceived authority of AI
		Limited evaluation of suggestions
		Avoidance of responsibility
	Minimal cognitive Engagement	Lack of critical reading
		No follow-up questioning
		Passive use habits
		Limited learning motivation
Adaptive AI engagement	Prompt-based adjustment	Modifying prompts
		Guiding AI outputs
		Expressing personal preferences
		Recognizing AI limitations
	Selective revision decisions	Judging appropriateness
		Filtering suggestions
		Rejecting unsuitable revision
		Using AI as reference
	Surface-level interaction	Attention to wording
		Focus on linguistic errors
		Limited attention to meaning and structure
Metacognitive AI engagement	Critical evaluation of AI feedback	Evaluating logic and meaning
		Developing personal criteria
		Considering language, culture, and intention
	Reflective revision awareness	Awareness of language development
		Reflection on AI use strategies
		Reflection on efficiency
	Transformation of Writing Understanding	Writing as thinking process
		Moving beyond correction
		Thinks about how to use AI effectively.
		Process-oriented writing

**Table 2:** Core themes, themes and coding derived from the qualitative data for RQ2.

Core themes	Themes	Coding
Initial writing	Baseline	Unawareness
		Misunderstanding of language issues,
		Lack of proofreading
		Language blind spots
AI-triggered awareness	Noticing	Noticing awkward phrasing
		Recognizing errors
		Identifying repetition
		Triggered awareness by AI suggestions
Revision regulation process	Monitoring and comparing	Comparing AI revisions
		Checking original text
		Examining context
		Going back and forth
	Strategic revision and decision-making	Trying alternative expressions
		Comparing structures
		Applying linguistic strategies
		Rejecting/accepting suggestions
Metacognitive reflection	Evaluation revision outcomes	Assessing suitability
		Clarity
		Stylistic fit
		Reflecting on writing quality

**Table 3:** Core themes, themes and coding derived from the qualitative data for RQ3.

Core themes	Themes	Coding
Redefining Writing	From product-oriented writing to meaning-oriented communication	Reader-oriented writing
		Clarity of expression
		Writing for meaning
	Writing as knowledge construction	Restructuring thoughts
		Organizing logic
		Cognitive construction
	Writing as an iterative AI-supported process	Recursive revision
		Non-liner writing
		AI-assisted writing process
Transforming English learning engagement	Increased engagement and idea development	Motivation boost
		Willing to write
		Idea stimulation
	Negotiating autonomy and dependency	Reliance on AI
		Loss of control
		Shifted motivation
Cognitive engagement and metacognitive regulation	AI-mediated cognitive transformation	AI-guided reasoning
		Deeper thinking through feedback
		Prompt-thinking alignment
	Metacognitive monitoring of AI use	Self-awareness
		Reflective alertness
		Evaluating AI suggestion
	Cognitive risks of overdependence	Cognitive laziness
		Reduced mental effort
		Concern about thinking decline
Negotiating AI’ role and writing identity	AI as a functional writing partner	AI-as-tool
		Companionship
	Limitations of AI assistance	Inconsistent feedback
		Lack of empathy
		Conditional trust
	Maintaining authorship and identity	Ownership protection
		Personal voice
		Resistance
Motivation towards AI-assisted writing	Attitude and Motivation	increased motivation
		willingness to reuse AI
		appreciation of AI collaboration

RQ1: Learners’ interaction patterns with Gen AI during writing revision

The findings identified three qualitatively different patterns of learner-AI interaction: passive AI reliance, adaptive AI engagement, metacognitive AI engagement (Table 1).

### Passive AI reliance

Passive AI reliance was characterised by uncritical acceptance and minimal cognitive engagement. The students showed blind acceptance of revision, perceived authority of AI, limited evaluation of suggestions and avoidance of responsibility. Comments such as *“It always writes better than I do”* and *“AI will fix my writing anyway”* reflected this reliance. Some participants also reported limited critical reading and motivation to engage further with AI feedback. One participant described GenAI simply as *“a spell checker”* and stated, *“I don’t expect it to teach me anything.”*

### Adaptive AI engagement

Adaptive AI engagement was characterized by learners’ selective and purposeful use of Gen AI during the revision (Fig 2). Rather than accepting AI-generated suggestions uncritically, these learners compared outputs with their intended meanings and adjusted prompts when the initial responses did not meet their expectations. Their engagement reflected an emerging level of agency, although their evaluations generally focused on wording, tone, and linguistic appropriateness rather than broader issues such as argument development or textual organization. They actively refined prompts, noticed surface-level language features, selectively accepted or rejected AI suggestions, and sought clarification when necessary. For example, one participant used prompts such as *“Use simple words”* or *“Make it more like my level.”* Another explained, *“Sometimes the AI changes the sentence too much, so I keep my original version because it sounds more like what I want to say.”*

### Metacognitive AI engagement

Metacognitive AI engagement was characterized by active reflection and critical evaluation throughout revision. Rather than accepting AI-generated revisions at face value, these learners assessed suggestions in relation to meaning accuracy, logical coherence, tone, cultural appropriateness, and alignment with their communicative intentions. They were also willing to reject revisions after considering their effects on the quality and authenticity of their writing. Comments such as *“It completely changed what I wrote”* and *“It doesn’t sound like my writing anymore”* demonstrated awareness of the distinction between polishing their writing and allowing AI to rewrite it. Some participants further reported that comparison with AI output helped them notice weaknesses in their own language use. One student stated, *“It made me realize my original sentence didn’t sound native,”* while another observed the original sentence was *“too Chinese in structure.”* More importantly, several participants began to reconceptualise revision as an ongoing process of thought and refinement. As one student explained, *“Writing is not just about producing a grammatically correct sentence; it is a gradual process of developing, revising and refining ideas.”* In these cases, interaction with GenAI supported metacognitive engagement through comparison, self-evaluation, and reflective decision-making rather than simply providing corrections. The value of AI-assisted revision therefore lay not only in the revised text itself but also in the processes through learners interpreted, questioned and evaluated AI-generated feedback (Fig 3).

**Fig 3:**
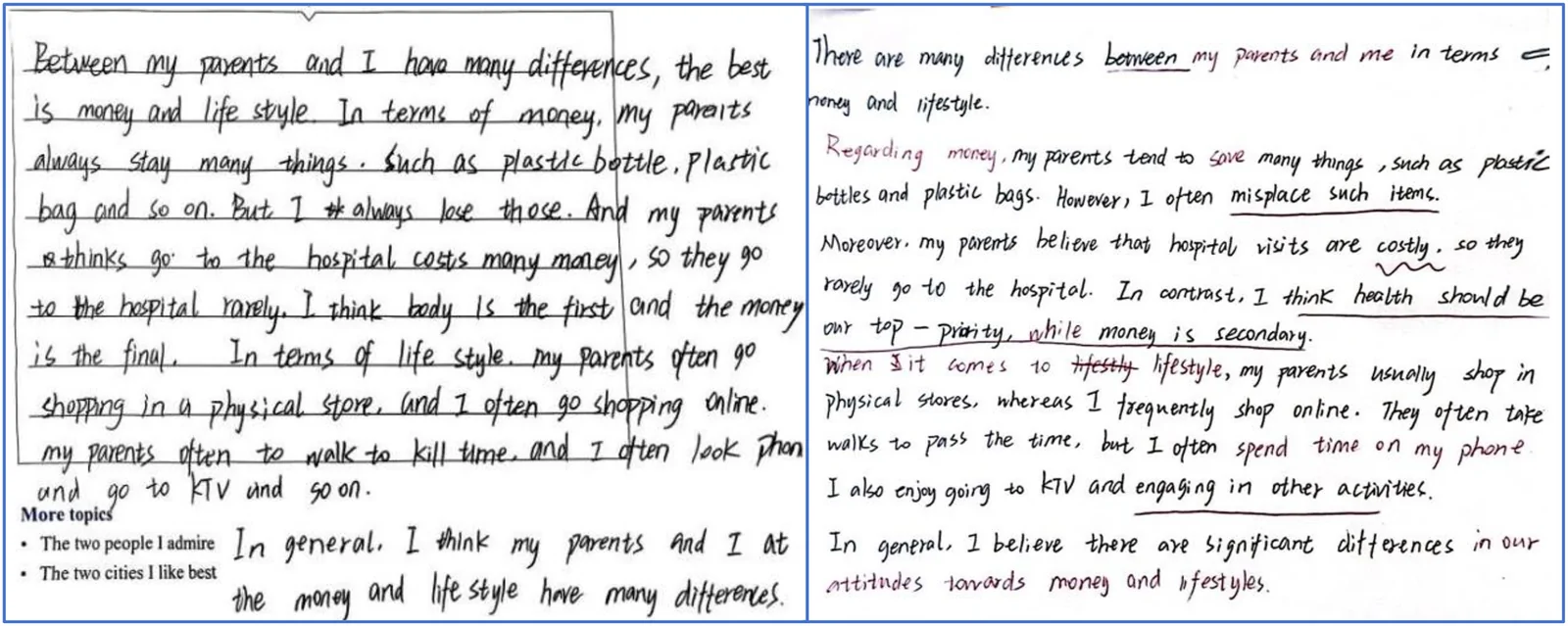
An example of a student who explore clarification through follow-up questions.

RQ2: The ways that EFL learners engage in metacognitive activities during Gen AI-assisted writing revision

Interview data revealed several forms of reported metacognitive engagement during AI-assisted revision (Table 2). As a baseline, many learners reported limited awareness of problems in their initial drafts before receiving AI feedback. Codes includes unawareness, of language issues, lack of proofreading and language blind spots. One participants admitted, *“I didn’t pay attention to the grammar at all at the time.”*

AI feedback subsequently activated learners’ awareness of writing problems. Participants reported that AI suggestions helped them notice previously overlooked issues, particularly at the linguistic level. For example, learners stated, *“I realised the sentence wasn’t smooth”* and *“I saw that AI pointed out my word repetition,”* indicating awareness of awkward expression, repetition and language accuracy. AI feedback that functioned as an external prompt that directed learners’ attention to previously unnoticed problems.

Following this awareness, learners engaged in revision regulation involving monitoring and strategic decision-making. Rather than simply adopting AI-generated revisions, learners compared AI suggestions with their original texts, examined contextual appropriateness, and moved repeatedly between the two versions. As one participant explained *“Even though it was accurate, it didn’t fit my writing.”* Some learners also experimented with alternative expressions, compared sentence structures, and applied linguistic strategies during revision. Decisions to accept or reject AI suggestions were based on their judgements of meaning, appropriateness, and writing intentions. For example, one participant stated, *“I didn’t take that suggestion from AI.”*

The later stage of revision involved metacognitive reflection on both writing outcomes and AI-assisted revision practices. Several learners evaluated whether AI suggestions improved their writing and reflected on how to use AI more effectively in future writing tasks. As one participant noted, *“I might still use it in the future, but I also want to think more on my own,”* suggesting an emerging awareness of the need to balance AI assistance with independent cognitive engagement. (Fig 4)

**Fig 4:**
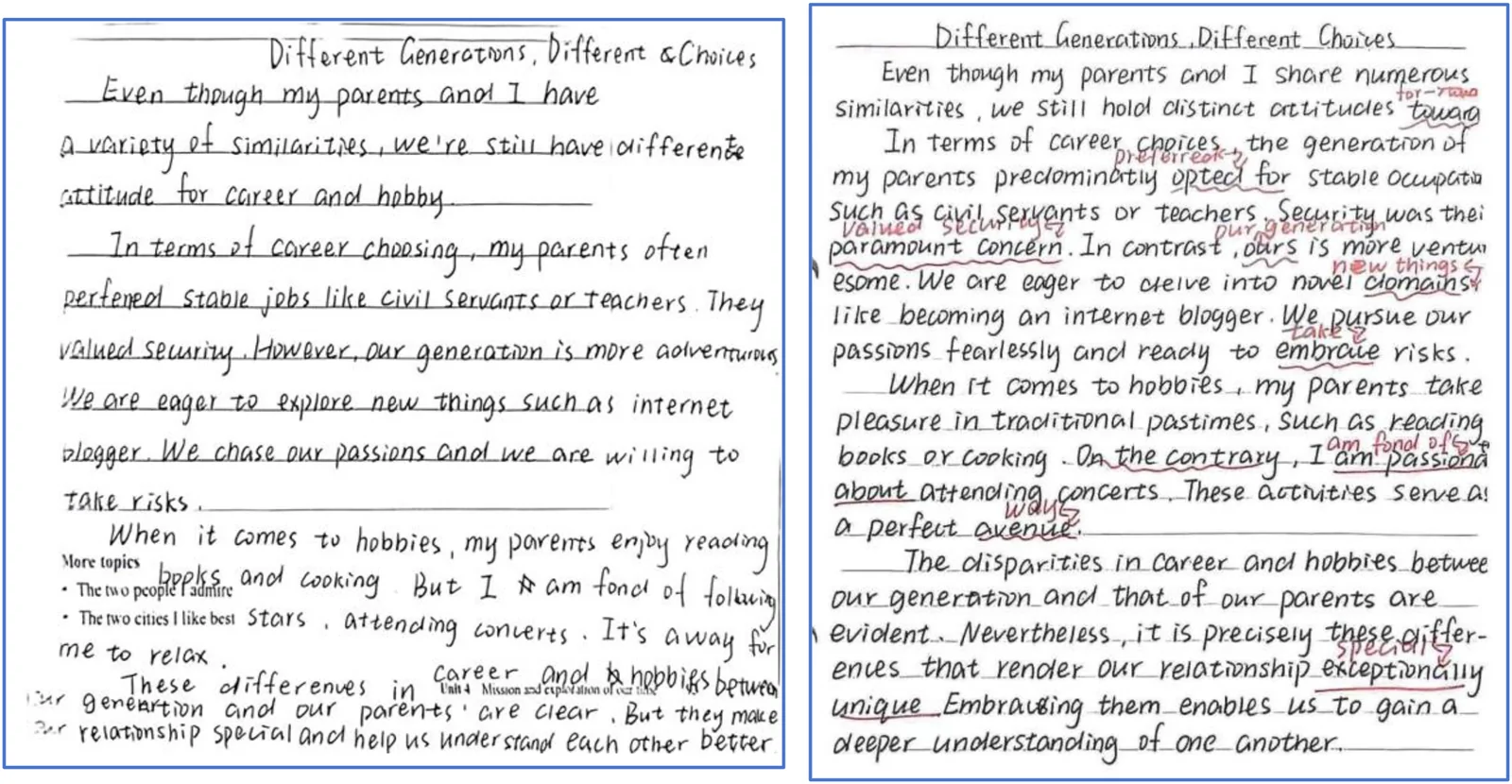
An example of a student who accept of feedback uncritically.

RQ3: EFL students’ perception of Gen AI in writing revision process and English learning

Interview findings revealed five dimensions of learners’ perceptions of GenAI-assisted writing revision: redefining writing, transforming English learning engagement, cognitive engagement, negotiating AI’s role and writing identity and motivation towards AI-assisted writing (Table 3).

First, learners began to reconceptualize writing as a meaning-oriented and iterative process rather than merely completing a writing task. Some participants reported revising from the reader’s perspective and paying greater attention to clarity and communication. Others described writing as a process of restructuring ideas and organizing logic through interaction with AI. Comments such as *“Sometimes I feel like I’m not the one writing anymore”.* These accounts reflected a shift from viewing writing primarily as text production towards seeing it as an iterative process of meaning development and refinement.

Second, learners perceived GenAI as affecting their engagement and motivation in different ways. Some participants became more willing to write because AI provided alternative expressions and stimulated ideas. As one learner noted, *“It feels easier to rewrite now because I can see more possibilities.”* Meanwhile, others expressed concerns about reliance on AI, loss of control, and reduced motivation to think independently, indicating a tension between increased engagement and dependency.

Third, learners reported changes in their thinking practices and metacognitive awareness when interacting with GenAI. Some participants noted that adjusting prompts and comparing AI outputs helped clarify their intentions and encouraged deeper reflection. However, learners also recognised potential cognitive risks, such as reduced mental effort and overdependence on AI. These accounts suggest that learners perceived cognitive benefits when they actively evaluated and regulated their use of AI.

Fourth, learners perceived GenAI as a useful but limited writing assistant. Participants appreciated its support in improving language expression and generating ideas but also noted its limitations in capturing personal voice, emotional tone, and contextual meaning. Some expressed concerns about whether extensively AI-revised texts still represented their own writing. Overall, learners negotiated the benefits of AI assistance while seeking to maintain authorship, autonomy, and control over their writing.

Finally, learners reported changes in their motivation and attitudes towards AI-assisted writing. Some participants described increased motivation to revise their writing and expressed willingness to use GenAI in future writing tasks. They also appreciated the interactive support provided by AI during revision. However, these positive attitudes were conditional on maintaining control over the writing process, suggesting that willingness to reuse GenAI was accompanied by awareness of the risks of overreliance.

## Discussion

### The pattern of Gen AI in EFL writing revision

The thematic analysis identified three patterns of learner engagement with GenAI in EFL writing revision: passive AI reliance, adaptive AI engagement, and metacognitive AI engagement. These patterns should not be interpreted as fixed learner categories or sequential developmental stages. Instead, they represent different ways in which learners interact with, interpret, and regulate their use of GenAI across writing contexts.

The first pattern, passive AI reliance, describes learners who primarily used GenAI as an external correction tool. They submitted their texts, received AI-generated suggestions, and often accepted revisions with limited evaluation [7,34,36]. While this approach enabled efficient access to linguistic support, it also created risks of cognitive dependency. Cotton et al. proposed that passive GenAI use may foster cognitive dependence by replacing some thought processes. Learners may become less activity involoved in cognitive processing when they can readily rely on such feature-rich system [35]. This finding suggests that access to automated feedback alone does not necessarily promote meaningful revision when learners engage with it uncritically.

The second pattern, adaptive AI engagement, reflects learners’ attempts to actively shape AI-assisted revision. These learners modified prompts, requested alternative expressions, compared outputs, and selectively accepted suggestions according to their writing goals. This pattern aligns with studies showing that well-crafted prompts can facilitate writing and enable AI to support content, organisation, grammar, and vocabulary [8,12,43]. Similarly, AI-assisted tools can help EFL learners correct linguistic errors and improve textual coherence and structure [10]. However, learners’ evaluations often focused on linguistic features such as wording, tone, and expression rather than deeper concerns related to argumentation or textual organisation. Adaptive engagement therefore represents an emerging form of learner agency, although critical evaluation remains limited (Fig 5).

**Fig 5:**
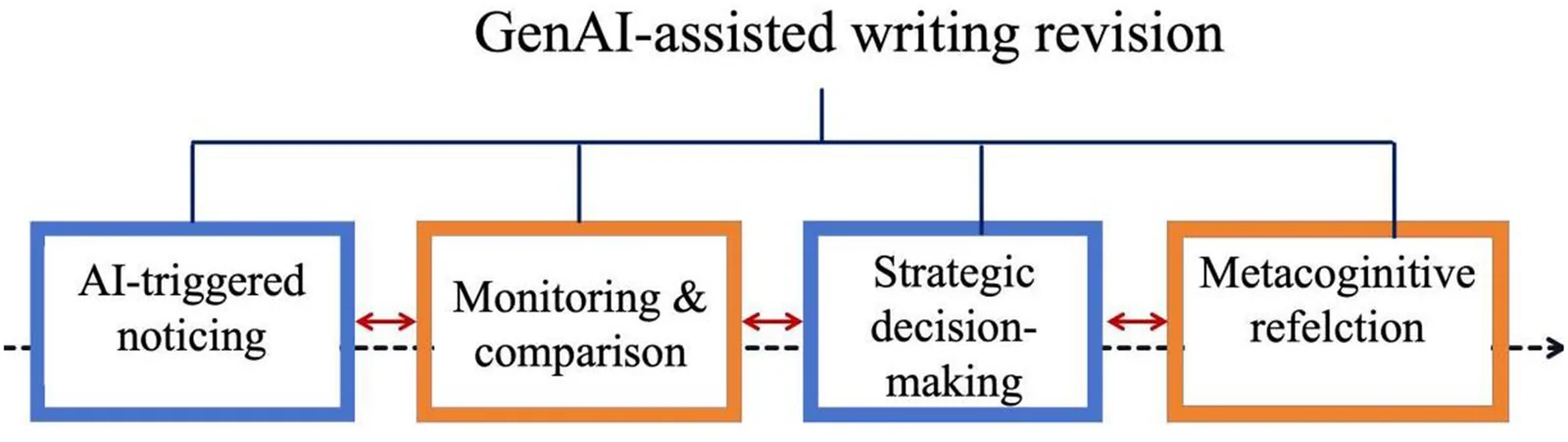
Metacognitive processes reported during GenAI-assisted writing revision.

The third pattern, metacognitive AI engagement, represents learners who actively monitored and evaluated AI-generated feedback. These learners considered whether AI suggestions matched their intended meanings, communicative purposes, and personal writing styles. They were willing to reject suggestions and reflect on their writing processes. This aligns with research emphasising that students should be empowered to think critically, contribute to disciplinary knowledge, and engage authentically with writing [44]. From this perspective, writing is not merely task completion but a cognitive and creative process requiring critical judgement and disciplinary thinking, for which AI is not a substitute [13]. Importantly, metacognitive engagement did not result from AI itself but from learners’ active interpretation and regulation of AI use. This finding is consistent with Fabio et al., who found that passive use of such resources may amplify heuristic thinking and compromise critical reasoning. Critical evaluation is therefore essential when learners select information and make revision decisions [45].

Overall, these patterns reveal a central tension between learner agency and dependency in AI-assisted writing revision. This finding resonates with research suggesting that the characteristics of AI can enable learners to assume responsibility for writing and claim greater authorship, while also creating risks to personal agency as AI becomes increasingly capable of anticipating users’ decisions and preferences. Learners must therefore avoid allowing AI to become a proxy “sage on the stage” that limits their agency and emancipatory potential [46]. These findings highlight the importance of task design and pedagogical approaches when integrating GenAI into writing instruction [45].

### Metacognitive engagement in GenAI-assisted writing revision

The findings revealed several forms of reported metacognitive engagement during GenAI-assisted revision, including noticing, monitoring, strategic decision-making, and reflection. These processes are not interpreted as sequential stages of development, but as different forms of engagement reported by learners within a single revision task. This finding extends previous research that has primarily emphasised GenAI’s capacity to provide immediate feedback and facilitate textual revision [10].

Participants reported that AI feedback drew their attention to linguistic or stylistic problems they had previously overlooked. This finding supports Schmidt’s noticing perspective, which emphasises conscious awareness of linguistic gaps as important for language development [14] and is consistent with research showing that insufficient metacognitive monitoring can prevent L2 learners from recognising discrepancies in their own writing [2]. However, noticing did not necessarily lead to deeper engagement, as some learners responded reactively by accepting corrections without further evaluation. This distinction extends previous research by showing that AI-triggered noticing and metacognitive regulation should not be treated as equivalent processes.

Metacognitive regulation was more evident when learners monitored AI feedback, compared alternative expressions, and selectively accepted, rejected, or modified suggestions. This finding is consistent with research linking effective revision to self-monitoring and strategic regulation [47]. It also supports studies showing that learners may negotiate between reliance and autonomy when interacting with GenAI [48]. The present findings suggest that the pedagogical significance of AI-triggered noticing depends partly on whether learners subsequently evaluate feedback and make intentional revision decisions.

Learners also reported reflecting on AI suggestions in relation to meaning, tone, rhetorical appropriateness, and authorial intention. This finding echoes research emphasising critical judgement in advanced writing and meaningful engagement with automated feedback [44,49]. Importantly, participants’ accounts indicated that evaluation sometimes involved protecting their intended meanings and personal voice. Rather than treating AI-generated feedback as authoritative, these learners questioned, modified, or rejected suggestions according to their writing intentions. This supports previous research on iterative AI-assisted revision [25], while demonstrating that such benefits depend on learners retaining control over revision decisions.

The findings also complicate claims that GenAI either promotes or undermines learner autonomy. Some learners reported increased confidence and motivation, whereas others recognised the risk of reduced cognitive effort. This tension is consistent with studies suggesting that AI can support learner agency while simultaneously encouraging dependency when used uncritically [26, 50]. Rather than producing either outcome inherently, GenAI appears to amplify different forms of engagement depending on how learners regulate its use.

Overall, the findings suggest that metacognitive engagement with GenAI cannot be inferred simply from learners’ exposure to AI feedback. Participants reported different forms of engagement, ranging from noticing linguistic problems to monitoring, strategic decision-making, and reflection. Given that these findings derive primarily from retrospective interviews following a single revision task, they should be understood as task-specific accounts rather than evidence of metacognitive development. The pedagogical issue is therefore not simply whether GenAI provides effective feedback, but how learners interpret, evaluate, and regulate their responses to that feedback.

### Learners’ perceptions of GenAI-assisted writing revision

The findings suggest that GenAI-assisted revision may have reshaped some learners’ perceptions of writing and English learning. Rather than viewing writing solely as the production of a correct final text, some participants described it as an iterative process involving planning, revising, and refining ideas. Several learners also became more aware of writing as structured thinking and meaning construction, echoing previous studies of AI-assisted writing [33,34]. This finding is consistent with research suggesting that AI tools can encourage learners to reconsider and reorganise their linguistic production [39]. The present study extends this work by showing that learners perceived revision not merely as correcting language but as progressively developing meaning. However, these perceptions emerged within a single revision task and should not be interpreted as evidence of lasting changes in writing beliefs.

Learners also reported greater engagement with revision when using GenAI. Previous studies have similarly found benefits of AI-assisted revision for linguistic accuracy, content organisation, and writing engagement [6,51]. In the present study, real-time and individualised feedback encouraged some learners to reconsider and reformulate their ideas, reflecting processes of noticing and reformulation [1]. Importantly, learners did not always accept suggestions passively; some selectively adopted feedback according to their rhetorical intentions. This finding supports research linking critical engagement with AI feedback to emerging learner autonomy and calibrated trust [29,52]. It also complements evidence that affective and autonomy-related factors shape learners’ engagement with AI-enhanced language learning [40]. Thus, increased engagement appeared to depend less on access to AI itself than on learners’ willingness to evaluate and regulate its support.

However, learners’ perceptions also revealed tensions surrounding authorship and personal voice. Some questioned whether extensively revised texts still represented their own writing. This concern echoes research warning that overreliance on GenAI may outsource not only linguistic production but also ideation and textual organisation [9,10]. Previous studies have similarly identified tensions between AI-supported empowerment and reduced learner control [11]. The present findings add to this debate by showing that learners themselves recognised this tension and sometimes rejected AI suggestions to preserve their intended meanings and authorial voice. This suggests that writing agency involves not simply using AI effectively but retaining control over what is ultimately represented as one’s own text [39].

Learners’ differing responses to AI-generated feedback further demonstrate the cognitive complexity of AI-assisted revision. For some, GenAI prompted reflection on lexical precision, logical progression, and meaning. This supports research suggesting that the quality of noticing, rather than mere exposure to corrective feedback, influences whether revisions contribute to learning [2]. In contrast, other learners accepted revisions with little critical evaluation, consistent with concerns that uncritical AI use may reduce cognitive investment and obscure learners’ actual writing competence [6,30,31]. Concerns about algorithmic opacity also emerged, echoing research showing that limited transparency can make it difficult for learners to evaluate the basis of AI-generated feedback [25]. Together, these findings suggest that the same technological affordances may support either reflective engagement or cognitive passivity depending on how learners respond to them.

An affective dimension was also evident in learners’ perceptions of GenAI. While many regarded AI as a helpful and efficient “companion”, they also recognised its limitations in emotional nuance and contextual understanding. This ambivalence reflects the importance of calibrated trust, whereby learners benefit from AI assistance without treating its output as inherently authoritative [29]. It is also consistent with research showing that students may increasingly perceive AI as a collaborative resource rather than merely a means of circumventing academic work [52–54]. The present findings suggest, however, that collaboration does not imply unquestioned acceptance; productive human–AI collaboration requires learners to recognise both the capabilities and limitations of AI.

Overall, learners’ perceptions of GenAI-assisted revision were multifaceted rather than uniformly positive or negative. GenAI was perceived as supporting motivation, agency, and metacognitive engagement when learners critically evaluated its feedback, while uncritical use was associated with concerns about dependency, authorship, and reduced cognitive investment. These findings therefore challenge deterministic views of GenAI as either inherently beneficial or detrimental to writing development. Its pedagogical value appears to depend on how learners interpret, regulate, and negotiate AI assistance while retaining responsibility for their writing.

## Conclusion

This study examined how EFL learners engage with GenAI during writing revision, focusing on their perceptions, revision practices, and metacognitive engagement. The findings suggest that GenAI functions neither merely as a surface-level editor nor as an autonomous writing guide. Instead, its pedagogical value depends on how learners interpret and regulate its use. While some learners demonstrated active evaluation, selective adoption, and reflective revision, others showed dependency, reduced cognitive effort, and limited critical engagement. These findings highlight the tension between learner agency and reliance in AI-assisted writing, suggesting that GenAI may support reflective and meaningful revision when learners retain control over decision-making and authorship.

Pedagogically, teachers should guide students to use GenAI as a reflective revision tool rather than an automatic correction mechanism through activities such as guided comparison, prompt refinement, and reflective revision. This study is limited by its primary reliance on self-reported interview data, which may involve recall bias and subjective interpretation, and by the absence of direct measures of learners’ cognitive processes. Future research could employ longitudinal designs, multiple data sources, and more direct measures of cognitive engagement to examine how learners’ engagement with AI relates to writing development across contexts.

## Supporting information

S1 Appendix Semi-structured interview protocol. (DOCX)

S1 File Interview data. (ZIP)

## References

[1] Sachs R, Polio C. Learners’ uses of two types of written feedback on a L2 writing revision task. Stud Sec Lang Acq. 2007;29(01). doi: 10.1017/S0272263107070039

[2] Qi DS, Lapkin S. Exploring the role of noticing in a three-stage second language writing task. Journal of Second Language Writing. 2001;10(4):277–303. doi: 10.1016/s1060-3743(01)00046-7

[3] Van de Poel K, Carstens WAM, Linnegar J. Text editing: a handbook for students and practitioners. Brussels: UPA; 2012.

[4] Lim WM, Gunasekara A, Pallant JL, Pallant JI, Pechenkina E. Generative AI and the future of education: Ragnarök or reformation? A paradoxical perspective from management educators. The International Journal of Management Education. 2023;21(2):100790. doi: 10.1016/j.ijme.2023.100790

[5] Stokel-Walker C, Van Noorden R. What ChatGPT and generative AI mean for science. Nature. 2023;614(7947):214–6. doi: 10.1038/d41586-023-00340-6 36747115

[6] Tsai CY, Lin YT, Brown IK. Impacts of ChatGPT-assisted writing for EFL English majors: feasibility and challenges. Educ Inf Technol. 2024;29(17):22427–45. doi: 10.1007/s10639-024-12722-y

[7] Li J. Research on AI-assisted Hybrid Teaching for English Writing. In: 2021 International Conference on Computers, Information Processing and Advanced Education (CIPAE), 2021. p. 309–12. 10.1109/cipae53742.2021.00080

[8] Stokel-Walker C. AI bot ChatGPT writes smart essays - should professors worry?. Nature. 2022:10.1038/d41586-022-04397-7. doi: 10.1038/d41586-022-04397-7 36494443

[9] Susnjak T, McIntosh T. ChatGPT: the end of online exam integrity?. Education Sciences. 2024;14(6):656. doi: 10.3390/educsci14060656

[10] Anson C, Straume I. Amazement and trepidation: implications of AI-based natural language production for the teaching of writing. JoAW. 2022;12(1):1–9. doi: 10.18552/joaw.v12i1.820

[11] Tran TTT. Enhancing EFL writing revision practices: the impact of AI- and teacher-generated feedback and their sequences. Education Sciences. 2025;15(2):232. doi: 10.3390/educsci15020232

[12] Ferris D. Response to students writing: implications for second-language students. USA: Lawrence Erlbaum Associates, Inc.; 2003.

[13] Al-Inbari FAY, Al-Wasy BQM. The impact of automated writing evaluation (AWE) on EFL learners’ peer and self-editing. Educ Inf Technol (Dordr). 2023;28(6):6645–65. doi: 10.1007/s10639-022-11458-x 36465424 PMC9684760

[14] Schmidt RW. The role of consciousness in second language learning. Applied Linguistics. 1990;11(2):129–58. doi: 10.1093/applin/11.2.129

[15] Hayes JR. A new framework for understanding cognition and affect in writing. The science of writing. Mahwah, NJ: Erlbaum; 2013.

[16] Weigle SC. English as a second language writing and automated essay evaluation. Handbook of Automated Essay Evaluation. Routledge. doi: 10.4324/9780203122761.ch3

[17] Mohsen MA, Alshahrani A. The effectiveness of using a hybrid mode of automated writing evaluation system on EFL students’ writing. Teaching English with Technology. 2019;19(1):118–31.

[18] Wang Y-J, Shang H-F, Briody P. Exploring the impact of using automated writing evaluation in English as a foreign language university students’ writing. Computer Assisted Language Learning. 2013;26(3):234–57. doi: 10.1080/09588221.2012.655300

[19] Link S, Mehrzad M, Rahimi M. Impact of automated writing evaluation on teacher feedback, student revision, and writing improvement. Computer Assisted Language Learning. 2020;35(4):605–34. doi: 10.1080/09588221.2020.1743323

[20] Huang S, Renandya WA. Exploring the integration of automated feedback among lower-proficiency EFL learners. Innovation in Language Learning and Teaching. 2018;14(1):15–26. doi: 10.1080/17501229.2018.1471083

[21] Li R, Meng Z, Tian M, Zhang Z, Ni C, Xiao W. Examining EFL learners’ individual antecedents on the adoption of automated writing evaluation in China. Computer Assisted Language Learning. 2019;32(7):784–804. doi: 10.1080/09588221.2018.1540433

[22] Chen C-FE, Cheng W-YEC. Beyond the design of automated writing evaluation: Pedagogical practices and perceived learning effectiveness in EFL writing classes. LLT. 2008;12(2):94–112. doi: 10.64152/10125/44145

[23] Boulanger D, Kumar VS. Explainable AI and AWE: balancing tensions between transparency and predictive accuracy. The Routledge International Handbook of Automated Essay Evaluation. Routledge; 2024. p. 445–68.

[24] Epstein Z, Hertzmann A, Investigators of Human Creativity, Akten M, Farid H, Fjeld J. Art and the science of generative AI. Science. 2023;380(6650):1110–1. doi: 10.1126/science.adh4451 37319193

[25] Feuerriegel S, Hartmann J, Janiesch C, Zschech P. Generative AI. Bus Inf Syst Eng. 2024;66(1):111–26. doi: 10.1007/s12599-023-00834-7

[26] Saputra I, Astuti M, Sayuti M, Kusumastuti D. Integration of artificial intelligence in education: Opportunities, challenges, threats and obstacles. A literature review. IJCS. 2023;12(4). doi: 10.33022/ijcs.v12i4.3266

[27] Kasneci E, Sessler K, Küchemann S, Bannert M, Dementieva D, Fischer F, et al. ChatGPT for good? On opportunities and challenges of large language models for education. Learning and Individual Differences. 2023;103:102274. doi: 10.1016/j.lindif.2023.102274

[28] AlAfnan MA, Dishari S, Jovic M, Lomidze K. ChatGPT as an educational tool: Opportunities, challenges, and recommendations for communication, business writing, and composition courses. JAIT. 2023. doi: 10.37965/jait.2023.0184

[29] Michel-Villarreal R, Vilalta-Perdomo E, Salinas-Navarro DE, Thierry-Aguilera R, Gerardou FS. Challenges and opportunities of Generative AI for higher education as explained by ChatGPT. Education Sciences. 2023;13(9):856. doi: 10.3390/educsci13090856

[30] Farrokhnia M, Banihashem SK, Noroozi O, Wals A. A SWOT analysis of ChatGPT: Implications for educational practice and research. Innovations in Education and Teaching International. 2023;61(3):460–74. doi: 10.1080/14703297.2023.2195846

[31] Sætra HS. Generative AI: here to stay, but for good?. Technology in Society. 2023;75:102372. doi: 10.1016/j.techsoc.2023.102372

[32] Hartmann J, Schwenzow J, Witte M. The political ideology of conversational AI: converging evidence on ChatGPT’s pro-environmental, left-libertarian orientation. arXiv. 2023. doi: 10.48550/ARXIV.2301.01768

[33] Saricaoglu A. The impact of automated feedback on L2 learners’ written causal explanations. ReCALL. 2018;31(2):189–203. doi: 10.1017/s095834401800006x

[34] Carvalho L, Martinez-Maldonado R, Tsai Y-S, Markauskaite L, De Laat M. How can we design for learning in an AI world?. Computers and Education: Artificial Intelligence. 2022;3:100053. doi: 10.1016/j.caeai.2022.100053

[35] Cotton DRE, Cotton PA, Shipway JR. Chatting and cheating: ensuring academic integrity in the era of ChatGPT. Innovations in Education and Teaching International. 2024;61(2):228–39. doi: 10.1080/14703297.2023.2190148

[36] Gao CA, Howard FM, Markov NS, Dyer EC, Ramesh S, Luo Y, et al. Comparing scientific abstracts generated by ChatGPT to real abstracts with detectors and blinded human reviewers. NPJ Digit Med. 2023;6(1):75. doi: 10.1038/s41746-023-00819-6 37100871 PMC10133283

[37] Javaid M, Haleem A, Singh RP, Khan S, Khan IH. Unlocking the opportunities through ChatGPT Tool towards ameliorating the education system. BenchCouncil Transactions on Benchmarks, Standards and Evaluations. 2023;3(2):100115. doi: 10.1016/j.tbench.2023.100115

[38] Fang Y. Perceptions of the computer-assisted writing program among EFL college learners. Educational Technology & Society. 2010;13(3):246–56.

[39] Liu H, Fan J. AI ‐Mediated Communication in EFL Classrooms: The Role of Technical and Pedagogical Stimuli and the Mediating Effects of AI Literacy and Enjoyment. Euro J of Education. 2024;60(1). doi: 10.1111/ejed.12813

[40] Liu H, Fan J, Xia M. Exploring individual’s emotional and autonomous learning profiles in AI-enhanced data-driven language learning: an expanded sor perspective. Learning and Individual Differences. 2025;122:102753. doi: 10.1016/j.lindif.2025.102753

[41] Révész A, Michel M, Lee M. Exploring second language writers’ pausing and revision behaviors: a mixed-methods study. Stud Second Lang Acquis. 2019;41(3):605–31. doi: 10.1017/S027226311900024X

[42] Fraenkel JR, Wallen NE, Hyun HH. How to design and evaluate research in education. 11 ed. Hightstown, NJ: McGraw Hill Publishing Co.; 2023.

[43] Praphan PW, Praphan K. AI technologies in the ESL/EFL writing classroom: The villain or the champion?. Journal of Second Language Writing. 2023;62:101072. doi: 10.1016/j.jslw.2023.101072

[44] Wise B, Emerson L, Van Luyn A, Dyson B, Bjork C, Thomas SE. A scholarly dialogue: writing scholarship, authorship, academic integrity and the challenges of AI. Higher Education Research & Development. 2024;43(3):578–90. doi: 10.1080/07294360.2023.2280195

[45] Fabio RA, Croce A, Calabrese C. Critical thinking in ethical and neutral settings in gifted children and non-gifted children. Children (Basel). 2022;10(1):74. doi: 10.3390/children10010074 36670625 PMC9856652

[46] Tsao J, Nogues C. Beyond the author: Artificial intelligence, creative writing and intellectual emancipation. Poetics. 2024;102:101865. doi: 10.1016/j.poetic.2024.101865

[47] Chang DH, Lin MP-C, Hajian S, Wang QQ. Educational design principles of using AI Chatbot that supports self-regulated learning in education: goal setting, feedback, and personalization. Sustainability. 2023;15(17):12921. doi: 10.3390/su151712921

[48] Zhang H, Ge S. Is Gen AI a professor in creativity? A case study in postgraduate students. Educ Inf Technol. 2025. doi: 10.1007/s10639-025-13644-z

[49] Nah F-H, Zheng R, Cai J, Siau K, Chen L. Generative AI and ChatGPT: Applications, challenges, and AI-human collaboration. Journal of Information Technology Case and Application Research. 2023;25(3):277–304. doi: 10.1080/15228053.2023.2233814

[50] Ryan RM, Deci EL. Intrinsic and extrinsic motivation from a self-determination theory perspective: definitions, theory, practices, and future directions. Contemporary Educational Psychology. 2020;61:101860. doi: 10.1016/j.cedpsych.2020.101860

[51] Lee Y-J. Can my writing be polished further? When ChatGPT meets human touch. ELT Journal. 2024;78(4):401–13. doi: 10.1093/elt/ccae039

[52] Zhao X. Leveraging Artificial Intelligence (AI) Technology for English Writing: Introducing Wordtune as a Digital Writing Assistant for EFL Writers. RELC Journal. 2022;54(3):890–4. doi: 10.1177/00336882221094089

[53] Tossell CC, Tenhundfeld NL, Momen A, Cooley K, de Visser EJ. Student perceptions of ChatGPT use in a college essay assignment: implications for learning, grading, and trust in Artificial Intelligence. IEEE Trans Learning Technol. 2024;17:1069–81. doi: 10.1109/tlt.2024.3355015

[54] Liu H, Song X. Exploring “Flow” in young Chinese EFL learners’ online English learning activities. System. 2021;96:102425. doi: 10.1016/j.system.2020.102425

