# Supplementary material for: Is Gen AI an editor or a revision tool? A case study of EFL students in AI-assisted revision
Source: PLoS One. 2026 Oct 29;21(10):e0359661. doi: 10.1371/journal.pone.0359661 (PMC13718921; doi:10.1371/journal.pone.0359661)
Supplement: Supplementary file 1 [file pone.0359661.s001.docx]

**Semi-structured interview**

1.	How frequently do you use AI tools in your daily life? What purposes do you usually use them for?

2.	Do you use AI tools for English learning, particularly English writing?

3.	Could you describe how you used Gen AI to revise your writing in a recent writing task?

4.	What types of feedback provided by Gen AI were most helpful for you? For example, grammar correction, vocabulary improvement, sentence restructuring, or adjustments to writing style.

5.	When using Gen AI for revision, did you always accept its suggestions, or did you sometimes modify or reject them? Could you provide specific examples and explain your reasons?

6.	After using Gen AI for revision, did you become more aware of aspects of your English writing that needed improvement? For example, recurring language problems or areas where your expression could be improved.

7.	After revising with Gen AI, do you think the final version better represented your original writing intentions? Why or why not?

8.	When reviewing AI-generated revisions, did you accept all suggestions, accept some suggestions, or reject most suggestions?

9.	How did you decide whether a suggestion should be adopted?

10.	How did you decide what prompts or instructions to provide when interacting with Gen AI?

11.	Did the process of using Gen AI make you feel more responsible for your own writing and learning? Could you share any specific examples?

12.	Do you think using AI tools has made you more independent in writing, or has it increased your dependence on external support? Why?

13.	Perceptions of writing and learning with Gen AI

14.	Did using Gen AI encourage you to think more deeply about your writing content or language choices?

15.	Do you think AI-assisted revision helped you reflect more on meaning and content, or did it mainly help with language-level corrections? Could you explain your experience?

16.	In your opinion, does using AI support or hinder your ability to develop original ideas in writing? Why?

17.	After this experience of AI-assisted writing, has your understanding of writing changed? If yes, how do you now perceive the writing process?

18.	Would you like to continue using AI as a self-learning tool for English writing in the future? Why or why not? How would you use it?

19.	Is there anything else you would like to share about how AI has influenced your writing, learning, or thinking processes?
