# Supplementary material for: Is Gen AI an editor or a revision tool? A case study of EFL students in AI-assisted revision
Source: PLoS One. 2026 Oct 29;21(10):e0359661. doi: 10.1371/journal.pone.0359661 (PMC13718921; doi:10.1371/journal.pone.0359661)
Supplement: Supplementary file 2 [file pone.0359661.s002.zip › Interview data/1.docx]

Interviewer: Do you like English?

Interviewee: Compared to what we're currently studying, I quite like advanced mathematics.

Interviewer: Why? Why do you like it more than advanced mathematics?

Interviewee: I feel... Do I need to compare? I feel... I don't like it that much.

Interviewer : Okay, how often do you use AI in your daily life?

Interviewee: Quite often, including my classmates.

Interviewer : In what situations do you usually use it?

Interviewee: For example, when the teacher asks questions, and in our English class, because the teacher requires us to write an English essay first. Then we submit two versions, the second version being AI-edited, and then we all call it out.

Interviewer : In other aspects of English, not just writing, is it allowed for you to use your phones to ask questions?

Interviewee: In other aspects. We use it quite often when the teacher asks questions in class. Yes!

Interviewee: Sometimes it lets us ask the AI.

Interviewer: Okay, so if it allows you to use it, do you use it in your daily life?

Interviewee: In daily life, for example, for those assignments. What might the assignment start with using AI to create an outline?

Interviewer : Even if he didn't ask you to use AI, right? Think back to this writing assignment; how did you use AI to polish your essay?

Interviewee : Mainly, I wrote it myself first, and then the AI acted as an instruction. Like an English professor asking you to revise my essay, and then seeing how the grammar and sentence structure became more sophisticated.

Interviewer: Of all the feedback it provided, which aspects were the most helpful?

Interviewee: It could point out the problems I didn't realize were there while writing. It could give us suggestions for improvement and make the essay more sophisticated.

Interviewer: What do you mean by "sophisticated"?

Interviewee: What aspects were more sophisticated? Russian. What synonyms can it use? Comparatively... it feels a bit complex. People usually use more colloquial language, but it provides a more formal, written version.

Interviewer: Do you think it includes any adjustments to grammar, vocabulary, sentence structure, or language style?

Interviewee: It will make some adjustments. I feel its structure is very clear, but when you try to connect it with real-life examples, it becomes very stiff and unnatural; it doesn't feel like something a human could write.

Interviewer: Can you share an example?

Interviewee: The example is something Teacher Shanshan assigned us for an essay. It was about using real-life examples, something related to success, like how to achieve success. When I used it, the example she gave me was stiff and didn't fit well; I had to revise it later.

Interviewer: You feel it doesn't fit your life because it provides examples?

Interviewee: It does have examples, but those examples aren't very suitable. It feels like a human could tell at a glance that it was written by AI.

Interviewer: When it gives you suggestions, do you...?

Interviewer : Have you ever rejected its suggestions?

Interviewee: I rejected it and asked it to revise the parts I felt were inappropriate, to see what it could do.

Interviewer : After using the AI, do you realize where you went wrong, or has your language improved?

Interviewee: It's very obvious. It can point out all my mistakes, grammar, etc., and it can quickly identify them.

Interviewer: Did you go back and check what it expressed?

Interviewee: His revisions were more advanced than mine, so I didn't check them much afterward. I checked them again, and I felt they were all correct.

Interviewer: You think he accurately expressed your writing intention, right?

Interviewee: As I just mentioned, he might be lacking in the example aspect, but everything else is fine.

Interviewer : I'll just take a quick look.

Interviewee: I'll let everyone take a look.

Interviewer: Okay, let me share my screen with you first.

Interviewee: Okay.

Interviewer : Hi. Is that your assignment this time? I see the parts marked in red are ones you revised, the parts marked in yellow are ones I noticed the AI provided, which you didn't modify. The AI didn't provide these sentence structures, why?

Interviewee: The AI gave it to me. Maybe when it's revising, there's some overlap, and some sentences it doesn't change, while others it makes more sophisticated.

Interviewer : You didn't use it. Why didn't you use it?

Interviewee: I didn't use it because I feel my own expression is okay. Some of what it gave me was too sophisticated, maybe not up to my current level.

Interviewer : You think it might be a bit fake, is that what you mean?

Interviewee : Yes!

Interviewer : Now, for example, when you submit your own assignments, you think these sentences are ones you can express yourself in the future.

Interviewee: Right!

Interviewer : The AI provides things you can use, but you might not think of how to use them later. Like the green highlighted area below, I see you wrote some improvement points. Was this provided by AI?

Interviewee: Yes, I asked it to give me some revision suggestions; it generated those for me.

Interviewer: Have you looked at these things since then?

Interviewee: Right after I finished writing, I would look at how it corrected me, and see if it improved my writing.

Interviewer: What aspects of your writing did you think it improved?

Interviewee : It makes some of my more colloquial parts more formal. It makes my sentence structure more sophisticated; it can combine my fragmented sentences into 11 sentences.

Interviewer: You understand. Okay, so in your writing process using AI, do you feel you've become more independent? Or have you become more reliant on AI?

Interviewee : Sometimes I feel like I’m not the one writing anymore. Before, when I wrote, I would try to find errors myself, but if I couldn't see them, I couldn't. But with AI, it can quickly point out my mistakes.

Interviewer : After it points out your mistakes, do you continue to learn and improve afterward?

Interviewee: Yes, it does help me improve, and it's quite useful. Looking back at my mistakes reduces the probability of repeating them.

Interviewer: When interacting with AI, what kind of commands do you think are most effective?

Interviewee: It's what I just mentioned, and what our teacher suggested: let the AI switch roles. Imagine you're a university English professor, and you can look at this essay and offer some suggestions for revision, something like that.

Interviewer : Since this is just one command—after it returns the revised version—do you provide any other commands?

Interviewee : Basically, no. Just ask it to revise it.

Interviewer: Have you considered using other commands, commands that are different from what your teacher provides, to make the revisions?

Interviewee : Not yet? It's something I need to do myself.

Interviewer : Do you feel that during the AI editing process, your English learning is affected? Do you feel it's your own responsibility, or is it just AI's?

Interviewee: AI mainly assists and helps us.

Interviewer: Do you think AI will affect your English learning?

Interviewee: It definitely has both good and bad effects. It can help me learn more sentence structures and vocabulary, but it can also make us more dependent on it. Sometimes it reduces our study time.

Interviewer: If you rely on it more, do you feel it will inspire your writing? Or the content of your writing, or give you more ideas about writing? Interviewee (00:14:29): With him, I just asked him to generate a simple outline for me, to give me some inspiration, and then I would write it down myself.

Interviewer: You would use this outline to brainstorm ideas for your article. Do you think the outline he provides can be further modified?

Interviewee: It can be further modified because I think the structure is quite clear. I would try writing it myself, and then have him revise it for me.

Interviewer: Do you always accept the suggestions he provides, or do you consider and perhaps reject some suggestions?

Interviewee: I tend to accept his suggestions; I feel they are quite objective.

Interviewer: Do you think AI makes you reflect on the content of your articles on a deeper level, especially regarding language expression?

Interviewee : It's more about language expression. I feel like it can't tell if my content has gone off-topic. It seems to give me more advice on structure and grammar, making me focus on the suggestions rather than the content itself.

Interviewer : It can influence your own thinking. So you need to grasp the content yourself, right?

Interviewee : The main thing is to stay on topic. I'd definitely be willing to use AI because I feel it's quite helpful for our learning.

Interviewer: So, in the future, would you be willing to use this AI as a self-study tool for English without a teacher's guidance? Could you elaborate on how it could help?

Interviewee: I might still use it in the future, but I also want to think more on my own. For example, when you write an essay or do a translation exercise, it can quickly point out your mistakes, help you correct them, and improve your translation skills.

Interviewer: Since it's about writing, have you used AI for learning in other areas?

Interviewee: After I finish translating from English to Chinese to English, I ask it to look at what problems there are.

Interviewer: What do you think of its translations?

Interviewee : I feel its language is quite advanced. Sometimes I just can't think of certain words, and it can give me some suggestions.

Interviewer: Do you think you've truly learned from the suggestions the AIgives you?

Interviewee: Anyway, I record what it expands, like expanding my vocabulary, and try to remember it.

Interviewer: In general, regarding AI-assisted writing, in terms of experience, do you feel that the writing process itself is different from your previous understanding?

Interviewee: The differences?

Interviewer: What was your original concept of writing quickly?

Interviewee: Before AI, I wrote entirely by myself, first outlining my ideas and then slowly writing. But with AI, the process seems to be much faster, don't you think?

Interviewer: Did you not put in a lot of effort?

Interviewee: I did write a little beforehand, but it felt like I didn't need to identify problems or ask teachers; I could just ask the AI.

Interviewer : What do you think of this method? Wang Chenyu (00:18:49): Actually, it's more about what the teacher taught. AI sometimes overlooks emotional aspects; it's rather rigid.

Interviewer : What do you think these emotional aspects include?

Wang Chenyu: Okay.

Interviewer : Yes...

For example, since you said it ignores emotion, what's an example?

Interviewee : It can directly translate things like rhetorical devices, but it lacks the beauty and doesn't quite capture the original meaning.

Interviewer: I think you must have had experience with AI before, or you must have had peer evaluations in middle school. What do you think?

Interviewer : Compared to peer evaluation, do you think AI is much better? Peers?

Interviewee: Anyway, I think its suggestions are quite useful, but it's not comprehensive enough. We might have students look at each other's work before, but it wasn't as efficient or accurate as AI.

Interviewer: Do you think it has any shortcomings?

Interviewee : Its shortcomings... Regarding the emotional aspects I just mentioned, and his/her emphasis on structure and sentence patterns...

Interviewer: Do you have anything to add about AI and your writing?

Interviewee: Nothing much, basically what you said, teacher.

Interviewer: Okay, then my interview is over. That's all. Thank you for participating and for sharing these important viewpoints.

Interviewee: Okay. Goodbye, teacher!

Interviewer : You can call the next person in now.

Interviewee: I'll call him/her.

Interviewer: Thank you, bye-bye!
