# Supplementary material for: Is Gen AI an editor or a revision tool? A case study of EFL students in AI-assisted revision
Source: PLoS One. 2026 Oct 29;21(10):e0359661. doi: 10.1371/journal.pone.0359661 (PMC13718921; doi:10.1371/journal.pone.0359661)
Supplement: Supplementary file 2 [file pone.0359661.s002.zip › Interview data/10.docx]

Interviewer : No need. The interview is about exploring the relationship between AI and English writing, so the content of our interview will not be known to others.

Interviewee : It's okay, feel free to speak!

Interviewer: Did you choose English as your major because you like English?

Interviewee : I wanted to study Chinese Language and Literature in the college entrance exam, but my score wasn't high enough, so I applied to almost every major and ended up getting into English.

Interviewer: How often do you use AI in your daily life?

Interviewee: Quite often. Thank you.

Interviewer: In what areas do you use it?

Interviewee: For example, when I need to write a proposal, or for an event I need to participate in, I use AI. I tell it the theme, it generates a proposal for me, and then I revise it based on what it generates. Also, sometimes when we do some less-than-ideal assignments, we might use AI to check them first, and then revise them based on our understanding of the course content.

Interviewer: Do you use AI a lot in your English learning process?

Interviewee: Actually, quite a bit.

Interviewee: For example, if you see a long passage and don't want to read it from beginning to end, you can just take a picture and send it to the AI. Then it will summarize the main idea of your article in the simplest language. It's quite convenient.

Interviewer: In English writing, how do you handle references?

Interviewee : I remember when I took the TEM-4 (Test for English Majors-Level 4) essay, the teacher told me that I could use AI to help me with the writing, to help me evaluate it. The AI helped me evaluate my writing, and then it could revise it based on what I wrote. I think I did that. The teacher assigned me three questions and made me write three essays, which I then sent to the AI. The AIsaid it had me grade them based on the TEM-4 (Test for English Majors-Level 4) grading criteria, and then, based on its own understanding, it revised them. I felt...

Interviewee: What Teacher He Zhen and Teacher Lin said is actually quite similar, but there are some parts that don't quite match the source.

Interviewee : There were discrepancies, so I looked at some official evaluation criteria for his specialties on Xiaohongshu (Little Red Book). I also found some of his additional criteria. Then I looked at them again.

Interviewer: Did you ultimately accept his work or reject it, because it differed from the evaluation criteria?

Interviewee: I feel like I only... The good part was the language corrections he made for me, such as the use of punctuation. There's another word, and I've checked the word choice and potential spelling and grammatical errors, but his evaluation criteria...

Interviewee: I just glanced at it. I asked him to base it on the 4P I standard, and I just had him review it briefly. I didn't really... really, really, really adopt his approach!

Interviewee : Right.

Interviewer : I understand. Have you used it to polish your papers?

Interviewee: Actually, it does exist in this field.

Interviewer: It depends on you.

Interviewee: Recently, it's not the first round of thesis proposals, so there might be some points that need expansion, or you could search for more information online, but you might not be able to write it as well yourself.

Interviewer: How did you use it?

Interviewee : If you ask him to expand on it, he might expand it even better, and then you can modify it based on what he expands. It seems pretty good. I feel it's most helpful, and its best use is helping you organize and understand information.

Interviewer: So, which aspect of the feedback he provides do you find most helpful?

Interviewee : Maybe you already have a certain understanding of the information. After he organizes it for you, you might have a clearer understanding based on his organization. The prerequisite is that you must give him the complete information; you can't just give him a little bit, and then what he generates will definitely be incomplete.

Interviewer: Have you tried, for example, feeding the TEM-4 scoring criteria you just mentioned from Xiaohongshu to the AI and letting the AI evaluate it?

Interviewee : I sent it to Doubao. The scoring criteria, and the one Doubao used before was the same one. I didn't tell him the scoring criteria, but the one he analyzed for me was almost identical to the scoring criteria for our TEM-4 exam. However, when I used DeepSick to check, it was completely different. So, I stopped using it and just used Doubao directly.

Interviewer: Even after you said you didn't give DeepSick the scoring criteria, he didn't use it accordingly.

Interviewee: I didn't use DeepSick; I used Doubao directly because Doubao was very similar to it before. Later, I memorized it again, and it was still about the same.

Interviewer: I understand. So, in the process of using AI for polishing, after the final version is given to you, do you clearly realize which aspects you are prone to making mistakes in order to improve yourself later?

Interviewee : Actually, I know, I feel... The improvement might be slower because writing requires, for example, punctuation in English writing. It involves distinguishing between half-width and full-width characters, and there's also the matter of grammar usage. When I write, I'll definitely make grammatical errors and spelling mistakes. If I'm writing on paper, I definitely can't recognize those. On a computer, spelling errors are underlined, so I can easily identify them, but when I'm actually writing, I can recognize my own mistakes.

Interviewee : With Deep Sick, I can be quite open. I dare to copy and paste, then modify it. I feel that way there's no problem. That's really my feeling if you experience it.

Interviewer : When you're learning on your own later, you might still trust AI more, and your own efforts won't be as significant.

Interviewee : You'll definitely think about the benefits of AI. You're thinking of writing about your practical work, for example, the actual writing might decrease.

Interviewer : Do you feel that in this process, you've become more independent? Or more reliant on AI?

Interviewee: It seems so. Actually, it always seems so. For example, if you're thinking about what you want to write, you're definitely always independent. You actually have expectations about what you're going to write—what you want it to look like, what your thought process is. But in the actual writing process, you definitely rely more on AI.

Interviewer: So, in the actual writing process, what aspects do you usually rely on AI for?

Interviewee : Specifically, how to use the lexical syntax. I might directly provide a framework, let them write it for me, and then refine it based on what they wrote, and then modify it myself.

Interviewer : Understood. What kind of instructions do you think you should give him so he can better understand what you want?

Interviewee: First of all, I feel that the teacher is mainly responsible for writing?

Interviewer : Okay, just a moment, I think I have a package to pick up.

Interviewee : First, I feel you should tell him your identity, what tone he should use to speak to you, and what role he should play. Secondly, you should give him your thought process. For example, in the first paragraph, what is the title, and how should the writing structure be? For example, in the first part, I should write an introduction, in the second part, I should analyze the reasons, propose corresponding measures, and finally write a summary. I feel the best way to give him this thought process is to tell him your reasons and your measures after you have thought them through. All right. Just give him all these specific things, and he will help you organize your logical thinking. I feel that this is quite clear.

Interviewee : Finally, you tell him what you want to write in your own style. You can give him a text and ask him to write it in the same style.

Interviewer : In the process of using AI, do you feel more responsible for your English learning?

Interviewee : It's okay, teacher. I don't feel more responsible.

Interviewer: So you're saying you've shifted the responsibility to AI? To AI?

Interviewer: So, for example, after the final score is generated, you feel that it's a score given by AI anyway.

Interviewee: That's impossible, but it might still be imposed on myself. This might also be a way of evaluating myself. I haven't thought about this question yet. I feel like I mostly evaluate myself, because I feel that evaluation isn't real; it doesn't reflect my true level.

Interviewer: Do you think your true level is higher?

Interviewee: What might be lower than the level of pollution?

Interviewer: Do you think your writing ability has decreased or improved after the advent of AI?

Interviewee : I feel that it has definitely improved, but not by much. It's effective, but not by much. Because it only generates my own thoughts.

Interviewer: Yeah.

Interviewee: When I actually practice writing on my own, I haven't done it. I feel that writing is something that still needs to be practiced by oneself. For example, I feel that not practicing is not enough. I feel that writing definitely has a purpose and a topic; it needs to be based on your own purpose and topic, and you need to express your own viewpoint.

Interviewer: So, what is your understanding of writing itself?

Interviewer : I mean writing in general, not just English writing. With the advent of AI, how has your understanding of this concept changed?

Interviewee: I feel it's an ability to process and manipulate information.

Interviewee: Perhaps you're using AI to help you process and manipulate information, but I don't think it can really help you.

Interviewer: Do you think it hinders or helps you to come up with original viewpoints?

Interviewee: Even though it's AI, it still has limitations. It might be based on what others have generated, and then use that to help you generate your own. It definitely needs to be based on other people's viewpoints to help realize its own.

Interviewer: Where do you think its limitations lie?

Interviewer : Do you think it's stealing other people's ideas? Do you think its viewpoints are correct?

Interviewee : Actually, after writing so much, I feel that some of its viewpoints are quite correct. In cases where they're incorrect, perhaps because your expression isn't clear enough, or perhaps it's because its suggestions aren't quite right. For example, if you ask it to suggest some measures regarding environmental protection, although these issues are quite general, I feel that the measures it proposes are quite practical.

Interviewer: I understand! Now, can you recall if you used peer evaluation or teacher evaluation in high school or other classes? Do you think AI would be a perfect evaluator?

Interviewee : Okay, I feel AI needs improvement. It's not that it's imperfect, but AI can indeed quickly identify some of your mistakes, but it's not as lifelike or expressive as a human.

Interviewer: What do you mean by lifelike or expressive?

Interviewee: For example, with scoring criteria, a human evaluates based on their own understanding of the criteria. When they evaluate an essay, they likely have their own interpretation. When you ask AI to evaluate your work, I feel it will be slightly faster than a human in terms of pronunciation, punctuation, spelling, and grammar. However, if you don't tell it the evaluation criteria, its evaluation might not be as accurate.

Interviewer: Do you have any other points you'd like to add regarding AI and English writing? Interviewee: I'd like to add something.

I feel for you. I think you can use AI to help you with your writing, but you can't completely rely on it. For example, don't just give it a template and expect it to write it yourself. You must review it yourself first, and don't just give it the top ideas and let it write it itself. You must base your writing on your own thoughts and ideas, let it generate the template for you, then combine it with what it generates, and finally refine it.

Interviewer: Anything else?

Interviewee : After all, AI isn't nonexistent, but it still relies on human judgment and doesn't have much emotion. I feel you still need to have your own judgment and thinking. For English writing, you need to have your own judgment and thought. Although AI is convenient, you can't rely on it too much.

Interviewer : That's true. Then I don't have any other questions.

Interviewee : It's alright, I'm honored to be able to help you, thank you, teacher, bye-bye!

Interviewer : Thank you very much! Thank you! Thank you! I hope your studies go smoothly! Bye!
