# Supplementary material for: Is Gen AI an editor or a revision tool? A case study of EFL students in AI-assisted revision
Source: PLoS One. 2026 Oct 29;21(10):e0359661. doi: 10.1371/journal.pone.0359661 (PMC13718921; doi:10.1371/journal.pone.0359661)
Supplement: Supplementary file 2 [file pone.0359661.s002.zip › Interview data/11.docx]

Interviewer: Daily AI usage frequency and scenarios:

Interviewee: I use AI quite frequently, about two or three times a day. Mainly for researching materials, outlining course assignments, and occasionally adjusting PowerPoint layouts. I also use it a lot in English learning, especially for writing essays. I almost always let AI guide my thinking before writing an essay—but I've always felt that no matter how useful AI is, it's just a tool; it lacks the "warmth" of a human being, which is the biggest difference between it and us.

Interviewer: The process of using AI to polish an article during a writing session:

Interviewee: Last time, when writing the English essay "The Influence of Campus Culture on International Students," after finishing the first draft, I pasted the content into AI and told it, "Help me clarify the logic, change the language to an academic style, and add some connecting phrases." It did make the paragraphs flow better, replaced "a lot of" with more formal phrases like "a considerable number of," and added connecting sentences, but it still felt a bit lacking. For example, when I wrote about the loneliness of international students arriving in China, the AI simply made the sentences smoother, but it couldn't convey the nuanced empathy that a human could. This is because it lacks human emotion.

Interviewer: The level of help from different types of AI feedback

Interviewee: The most useful features for me were sentence restructuring and language style adjustments! I often write convoluted sentences, and the AI helps me make them cleaner and smoother. Vocabulary replacement was also okay, but I had to choose it myself; sometimes it picked words that were too obscure and unsuitable for assignments. However, I used grammar correction less; I would check grammar software after writing. But it couldn't handle emotional optimization—after all, it lacks genuine emotion and can't understand the feelings behind the words, so it couldn't help me strengthen the warm expression.

Interviewer: Whether to fully accept AI suggestions

Interviewee: Definitely not! Except in cases of conflicting viewpoints, I would revise the parts involving emotional expression myself. For example, when I wrote a short English birthday greeting, the AI made the content very elaborate, but it lost the "personality" and genuine concern I wanted to express. This kind of content requires emotion; the AI's version felt too stiff. I immediately reverted to my original words. After all, it lacks human emotion and cannot express that unique feeling.

Interviewer: Understanding my own shortcomings after using AI polishing:

Interviewee: I have a clearer understanding of two aspects! On the one hand, I recognize my own language flaws, such as the repetitive use of "however" and awkward word order in relative clauses. On the other hand, I understand the limitations of AI—it can help me optimize "how to write," but it cannot help me convey the emotion of "why I wrote it this way." For example, when writing the English essay "My Childhood," I wanted to express my longing for my grandmother. The AI could only correct the grammar, but it couldn't capture the warmth of those memories. This made me even more certain: while AI is good, the biggest difference between it and a human is that it lacks emotion; emotional expression still requires personal effort.

Interviewer: Does polishing more accurately express the writing intention?

Interviewee: For objective writing, yes, but for emotional writing, it's not necessarily the case! For example, when writing practical assignments like "English speech skills," the AI helped me tightly integrate examples and viewpoints, resulting in accurate expression. However, when writing an essay requiring emotion, such as "love for one's hometown," the AI made the language smoother, but it lost the homesickness and longing I originally wanted to convey—after all, it lacks genuine emotional experience and cannot naturally reproduce such delicate intentions.

Interviewer: Criteria for deciding whether to adopt AI suggestions

Interviewee: Mainly based on three points: whether the viewpoint is correct, whether the language is natural, and whether it involves emotion. If the content aims to convey sincerity and deep affection, I generally don't use all of the AI's suggestions because it lacks human emotion; the resulting text is too "cold," unlike my own words.

Interviewer: Does AI make writing more independent or more dependent

Interviewee: Still, it's "half-relying on it, half-relying on myself!" It helps me quickly revise language and make the logic flow, saving time; however, I must express my own viewpoints and emotions—AI can make sentences look beautiful, but it can't help me generate genuine feelings about an event. For example, when writing about "having a conflict with a friend and then making up," the feelings of guilt and cherishing the relationship can only be conveyed through my own reflection. This part cannot rely on AI, which makes me more aware that my value lies in having emotions and the ability to think.

Interviewer: How to ask/instruct AI when interacting with it:

Interviewee: I will tailor my approach to different situations! When correcting objective assignments, I will be very specific, such as "Help me polish this essay on the impact of AI on English writing, connecting examples and viewpoints, and changing repetitive verbs"; but when writing emotional content, I will only ask it to "correct grammatical errors, but avoid sentences expressing emotions," because I know it doesn't have human emotions, and too much correction would lose the original intention.

Interviewer: Does using AI to polish increase my sense of responsibility for learning

Interviewee: Yes! On the one hand, I want to fill in the language gaps pointed out by the AI; on the other hand, I want to practice my own emotional expression—since the AI lacks emotion and can't help me convey genuine feelings, I have to practice myself. For example, when writing English letters, I deliberately use more nuanced words to express care and gratitude, making the writing warmer, not just "correct."

Interviewer: Would you be willing to use AI as a self-study tool for English writing in the future?

Interviewee: Yes! I plan to use it to practice IELTS chart writing, letting it score me and point out grammatical problems; but for essays, I'll still figure out the viewpoints and emotional expression myself. After all, AI lacks human emotion and can't write compelling content; this part requires personal accumulation.

Interviewer: Does AI inspire reflection on writing content/language?

Interviewee: Yes! Especially reflection on "how to convey emotion through language." For example, after the AI corrects a sentence, I compare: "My writing is warm but the language is rough, the AI's is fluent but cold, how to balance?" Gradually, I've learned to retain my own emotions while using the AI's language skills—making the writing smooth without losing emotion.

Interviewer: Does AI make you reflect on content or remain at the language level? Interviewee: More reflection on "how emotions are conveyed through language!" AI helps me overcome the language hurdle, giving me the energy to think: "How to express genuine feelings with simple words?" For example, when writing "Thank you for your help, teacher," AI might use flowery honorifics, but I feel "your patience and care really touched me" is more authentic because it's my genuine feeling—AI doesn't have that feeling, so it won't write it that way. This also makes me pay more attention to the emotional core of the content.

Interviewer: Does AI help or hinder original viewpoints?

Interviewee: It assists in originality, but emotionally driven originality still depends on oneself! For example, I have an idea that "good English writing needs emotional resonance." AI can help me list phenomena where "articles without emotion are stiff," but it can't help me write about cases I've personally experienced and can resonate with—these original works that require emotional experience can only be done by myself; AI cannot replace them.

Interviewer: A change in the understanding of "writing" itself

Interviewee: Previously, I thought writing was just "writing correctly and fluently," but now I think it's a combination of "emotion + expression"—language is the vehicle, emotion is the soul. AI can refine the medium, but it can't give it a soul. This has made me realize that the core of writing is the existence of "me," my unique emotions and experiences—something AI can never replace.

Interviewer: Any additional content?

Interviewee: I'm increasingly convinced that AI is just an "efficient language tool," not a "good writer." Even though my own writing sometimes has grammatical errors, it still conveys a raw, unrefined sentiment—this is the biggest difference between AI and humans: it lacks genuine emotion and cannot write with warmth and soul. In the future, I will remember that writing requires both using AI to optimize language and preserving one's own emotions and thoughts; this is the true meaning of writing.
