# Supplementary material for: Is Gen AI an editor or a revision tool? A case study of EFL students in AI-assisted revision
Source: PLoS One. 2026 Oct 29;21(10):e0359661. doi: 10.1371/journal.pone.0359661 (PMC13718921; doi:10.1371/journal.pone.0359661)
Supplement: Supplementary file 2 [file pone.0359661.s002.zip › Interview data/12.docx]

Interviewer: How frequently do you use AI in your daily activities? In what areas? How often do you use it for English learning? And English writing?

Interviewee: I use AI quite frequently, about 1-2 times a day. Mainly for checking course materials, organizing teaching design frameworks, helping to revise first drafts of English assignments, and occasionally using it to organize the ideas for assignments comparing Chinese, English, and French cultures.

Interviewee: I use it a lot in English learning, especially for English writing—whether it's short course essays or research reports, I always use AI to smooth out the logic after finishing the first draft.

Interviewer: Please describe how you used AI to polish your writing in a particular writing assignment?

Interviewee: Last time, when I wrote a short English essay titled "Family Affection in Scrambled Eggs with Tomatoes," after completing the first draft, I copied the content into AI and told it, "Help me refine the language style of this article, add more detailed descriptions of the mother-daughter interaction, and replace some Chinese expressions with natural English phrases (for example, instead of directly translating 'scrambled eggs' as 'fry eggs,' use the more common 'scrambled eggs')." It first changed my sentence "Mom taught me to cut tomatoes" to "Mom guided my hand as I sliced the tomato, her fingers covering mine to steady the knife," and added the detail of "tomato juice splashing onto my sleeve." Then it replaced the awkward phrases with more idiomatic expressions, making it much smoother to read. I realized sometimes my writing too Chinese in structure.

Interviewer: What types of feedback from AI are most helpful to you? For example, grammar correction, vocabulary replacement, sentence restructuring, and language style adjustment.

Interviewee: The most useful to me are vocabulary replacement and sentence restructuring!

Interviewee: For example, I often use "very important," and the AI will replace it with more precise words like "vital" or "essential." Sentence restructuring is also very useful—my long sentences tend to be convoluted, and the AI can transform them into concise and fluent short sentences without losing the original meaning.

Interviewee: I occasionally use language style adjustment, but I have to filter them myself; I use grammar correction less often, as I check my work with grammar software after I finish writing.

Interviewer: When using AI, do you completely accept its suggestions, or do you sometimes reject or modify them? Can you give an example explaining why?

Interviewee: I don't completely accept them!

Interviewee: For example, when I wrote an English report on "Cultural Differences in Toasting Between China, the UK, and France," the AI changed my view that "toasting in China is about 'personal connection'" to "toasting in China is about 'social etiquette'"—this weakened the core emotional connotation, so I changed it back. After all, my arguments revolved around "personal connection," and the AI didn't understand the emotions behind this culture; the change actually went astray.

Interviewer: After using AI editing, are you more aware of where you tend to make mistakes or where your language expression needs improvement?

Interviewee: Yes! For example, every time AI edits my work, I find myself repeatedly using the same conjunction (like "however" too often), and the word order in relative clauses is awkward. Last time, when writing a short essay about family, AI corrected my grammatical error "the tomato which I cut it" to "the tomato I sliced," and I realized I often add extra objects. Since then, I've paid special attention to this.

Interviewer: After editing, do you feel the final version more accurately expresses your writing intention?

Interviewee: It depends: For objective content, yes; for emotional content, not necessarily.

Interviewee: For example, when writing about "the differences in etiquette in toasting culture," the AI helped me connect the case studies and viewpoints more closely, resulting in accurate expression. However, when writing about "motherly love in scrambled eggs with tomatoes," the AI's editing made the language smoother, but it lost the gentle feeling of "a mother's hand covering my own" that I originally wanted to convey—after all, it lacks genuine emotional experience and cannot accurately reproduce such delicate intentions.

Interviewer: When you see AI polishing, do you accept it completely, partially, or not at all? How do you decide whether to adopt it?

Interviewee: I mostly accept it partially. My criteria are threefold:

① Does it align with my core viewpoint?

② Is the language natural (does it sound too "machine-like")?

③ Does it involve emotional/cultural connotations? If it requires conveying emotions like "human touch" or "affection," or "the cultural core behind toasting," I would be cautious about using AI suggestions because it lacks these experiences, and editing it might lose its essence.

Interviewer: Do you feel that using AI tools has made you more independent in the writing process, or more reliant on external help? Why?

Interviewee: It's "semi-independent, semi-dependent": It depends on it to help me quickly optimize language and logic, saving a lot of time revising initial drafts; but it's independent in that the viewpoints and emotional expression must be my own—for example, the core feelings in a short essay about family affection, the emotional connotations of toasting culture—the AI can't think of these for me. This makes me clearer: my value lies in having emotions and thoughts; the AI is just an auxiliary tool.

Interviewer: When interacting with AI, how do you decide what questions to ask or what instructions to give?

Interviewee: I'll be very specific, giving instructions based on the scenario:

For example, when revising an objective report, I'd say, "Help me add the case study of 'British gentlemanly toasting culture' to this report on toasting culture, and replace three repeated verbs".

Interviewee: If it's writing an emotional short essay, I'd say, "Help me correct the grammatical errors in this family affection article, but don't change sentences expressing details"—after all, I know the AI has no emotions, so I need to limit it from touching my core expression.

Interviewer: Does using AI for polishing make you feel more responsible for your English learning or writing? Do you have any specific examples to share?

Interviewee: Yes! For example, last time the AI pointed out that I kept using the subjunctive mood incorrectly, so I specifically reviewed my grammar book and did a set of subjunctive mood exercises. After all, I can't rely on AI to correct everything; I have to fill in the gaps myself—especially since I'll be a teacher in the future, I need to solidify my language foundation first. That's where this sense of responsibility comes from.

Interviewer: Would you be willing to use AI as a self-study tool for English writing in the future? Why? How?

Interviewee: Yes! The reason is that it can help me practice language efficiently (such as correcting grammar and refining expression); but I will combine it with my own reflection. Sometimes the AI suggestion helps me notice a problem, and then I quickly adjust the sentence to make it clearer. For example, I'll write a short English essay every day, let the AIscore it and point out problems, and then I'll summarize the types of errors and practice accordingly—I won't just rely on the AI to correct everything and then ignore it; I'll treat it as an "error correction assistant," not a "writing ghostwriter."

Interviewer: In the process of using AI, has it inspired you to think more about the content or language of your writing?

Interviewee: Yes! For example, when AI changed my sentence "Mom taught me to cook" to "Mom walked me through stirring the eggs," I pondered, "Why is 'walk through' more evocative than 'teach'?" Gradually, I learned to use more nuanced verbs to express emotions. Also, when writing about the culture of clinking glasses, AI added the detail that "in France, people look at each other when clinking glasses," which prompted me to research "why eye contact is a form of etiquette," expanding the depth of the content.

Interviewer: Do you feel that using AI has made you reflect more on the content expression of your writing, or is it merely at the language level? Could you elaborate?

Interviewee: Mainly at the language level, but occasionally it extends to the content.

Interviewee: For example, after AI helped me revise the language of my short essay about family affection, I suddenly realized that my example only mentioned "cutting tomatoes," omitting the detail of "Mom added a spoonful of sugar"—this is a deficiency in content, something I only realized while revising the language. But most of the time, AI can only help me optimize "how to say it," while "what to say (content, emotion)" still requires my own reflection, since it lacks genuine thought and feeling.

Interviewer: In your opinion, does AI help or hinder you from developing original viewpoints in your writing?

Interviewee: It assists on originality!

Interviewee: For example, I had a vague idea that "good writing requires 'emotion + detail'," and AI helped me list examples of how "emotion without detail is hollow," making my viewpoint more concrete. However, the core original viewpoint (such as "the culture of toasting is a vehicle for emotion") still had to be refined by myself. AI can help me "ground" my viewpoints, but it can't help me "generate" them.

Interviewer: Overall, has your understanding of "writing" itself changed through this experience of using AI-assisted writing?

Interviewee: Yes! I used to think writing was just about "writing correctly and smoothly," but now I think writing is a combination of "emotion + expression"—language is the vehicle, but emotion and thought are the soul. AI can refine the vehicle, but it can't give it the soul. This made me realize that the core of writing is "my" existence; I have unique emotions, experiences, and thoughts, which AI can never replace.

Interviewer: Besides the questions above, is there anything else you'd like to share about the impact of AI on your writing, learning, or thinking methods?

Interviewee: I think the biggest impact of AI is that it has taught me to be a "selector"—before, writing was "thinking + writing," now it's "thinking + AI helping me optimize + me selecting and adjusting." In this process, I have to judge whether the AI's suggestions are correct and whether they fit my style, which has actually honed my discernment.

Interviewee: Moreover, I'm increasingly clear that AI is a "tool," not an "opponent." It can save me time, but the core of learning is still "my active thinking and active accumulation"—for example, emotional expression and cultural connotations can only be experienced and pondered by myself; this is the true meaning of learning.
