# Supplementary material for: Is Gen AI an editor or a revision tool? A case study of EFL students in AI-assisted revision
Source: PLoS One. 2026 Oct 29;21(10):e0359661. doi: 10.1371/journal.pone.0359661 (PMC13718921; doi:10.1371/journal.pone.0359661)
Supplement: Supplementary file 2 [file pone.0359661.s002.zip › Interview data/13.docx]

Interviewer: how often and how often do you use AI?

Interviewee: About 3 times a day on average.

Interviewer: About. In what areas do you use it?

Interviewee: Mainly for information retrieval, or for example, writing, or finding inspiration, and also in my studies.

Interviewer: Okay, in English writing, do you use it a lot in your English learning?

Interviewee: Relatively a lot.

Interviewer: And English writing? Interviewee: I also do a lot of English writing because it helps me brainstorm, check grammar, and polish my writing.

Interviewer: Could you describe how you used AI to polish your writing in a particular writing task?

Interviewee: I remember once I needed to write an argumentative essay about the alienation of interpersonal relationships in the digital age. I first completed my draft, built the framework, and filled in the core arguments and points. Then I input my draft into the AI, giving it the instruction to polish the English essay, making its expression more fluent and academic, and checking the logical connections.

Interviewer: Okay, what types of feedback from the AI were most helpful to you?

Interviewee: I think the most helpful were sentence restructuring and language style adjustments. Because the grammar correction and vocabulary substitution are basic, they can quickly correct my basic mistakes. However, sentence restructuring allows me to see how to combine a simple sentence, or several simple sentences, into a complex and idiomatic long sentence. This makes the article more rhythmic. And then, adjusting the language style guides my writing from colloquial expressions to a more formal academic writing style.

Interviewer: So, when you use AI, do you completely accept its suggestions, or do you reject or modify them?

Interviewee: I partially accept them; I don't completely adopt all of its suggestions.

Interviewer: Can you give an example to illustrate why?

Interviewee: For example, in my article about interpersonal relationships, I wrote a sentence: "Technology makes us connected but also lonely." The AI's suggestion was: "paradoxically, while technology has interconnected individuals across the globe, it has surprisingly in that it engendered a profound sense of isolation." This modification is very elegant, and its vocabulary is also very sophisticated. However, I hesitated at the time because while the expression "in dan- in gendered a profound sense of solution" was accurate, it was too formal and didn't quite match the overall tone of my article. So, I ultimately opted for a compromise. I chose to keep "paradoxically" and the more concise "connects us globally." This ensured the sentence retained the tone I wanted after the revision.

Interviewer: Okay, after using AI editing, are you clearly aware of which aspects of your writing you tend to make mistakes in? Or where your language expression needs improvement?

Interviewee: By repeatedly comparing the AI's suggestions, I clearly saw my weaknesses. I found myself tending towards overly simple sentences and repetitive conjunctions, such as "and," "but," and "buts." The AI also made me realize the importance of sentence variety and vocabulary richness in English writing. Now, when writing, I consciously remind myself to try using different clauses and phrases to enrich sentence structure.

Interviewer: So, after you've finished polishing it? Do you feel the final version better expresses your ideas? Does it accurately convey your writing intent?

Interviewee: In most cases, yes, because AI acts like a high-powered magnifying glass. It helps uncover the ambiguous areas where I understood but couldn't articulate them. Through its restructuring and word substitution, the final version usually expresses my thoughts more precisely and powerfully, making my writing intent clearer to the reader.
