# Supplementary material for: Is Gen AI an editor or a revision tool? A case study of EFL students in AI-assisted revision
Source: PLoS One. 2026 Oct 29;21(10):e0359661. doi: 10.1371/journal.pone.0359661 (PMC13718921; doi:10.1371/journal.pone.0359661)
Supplement: Supplementary file 2 [file pone.0359661.s002.zip › Interview data/14.docx]

Interviewer: Okay, our first question is: How often do you use AI in your daily activities? In what areas do you mainly use it? How much do you use it in English learning, especially in English writing?

Interviewee: I use AI quite frequently in my daily life. For example, I use it to look up information and organize study notes. Occasionally, I also use it to assist in writing some English articles. I use AI quite often in my English learning, after all, journalism majors have certain requirements for English reading and writing skills.

Interviewer: Could you describe in detail how you used AI to polish your article in a particular English writing task?

Interviewee: Let me give you an example from one of my articles, an English commentary on media convergence. After finishing the first draft, I send it to AI to help me with some simple vocabulary correction, or to make simple words more sophisticated. It helps me replace some words with more advanced ones, and uses more precise academic expressions. It adjusts the word order to make my logic stronger and the expression smoother.

Interviewer: Hmm, it sounds like the AI feedback is quite comprehensive. So, among these feedbacks, which type helps you the most? Is it grammar correction, vocabulary replacement, or sentence restructuring and style adjustment?

Interviewee: I think vocabulary replacement and language style adjustment help me the most. News writing is very particular about style, and sometimes I confuse formal and semi-formal expressions. The AI helps me point out where I need to be more rigorous, where I can be more flexible. And the vocabulary recommended by the AI isn't particularly obscure, so I can improve the professionalism of my writing. Using AI is much more efficient than looking up words in a dictionary yourself.

Interviewer: So, do you completely accept the AI's suggestions, or do you reject or modify them? Could you give an example explaining why?

Interviewee: I rarely accept everything completely; most of the time I adopt only a small part. For example, last time I wrote a lead for a news report. The AI suggested I change what was originally a simple summary into a more complex compound sentence, saying that this expression would be more eloquent. But a news lead needs to be straightforward; too much complexity would feel superfluous. So I only adopted one professional term it recommended, ensuring both efficient information delivery and optimized expression.

Interviewer: After using AI for polishing, do you become clearer about where you tend to make mistakes in your writing, or in what areas you need to improve?

Interviewee: Yes. For example, I saw that AI pointed out my word repetition in articles and misuse of conjunctions between parallel sentences. This made me realize that although my English score on the college entrance exam was not low, my basic grammar details were not solid enough. It also made me pay more attention to the overall coherence of the article, rather than the correctness of individual sentences.

Interviewer: Does the revised version more accurately express your writing intention? How do you judge whether to adopt the AI's suggestions?

Interviewee: Most of the time, yes. The AI can help me identify ambiguous parts and make my intention clearer. There are two main criteria for judgment: one is whether it meets the requirements of the writing task, and the other is whether it aligns with my original thought process.

Interviewer: With long-term use of AI-assisted writing, do you feel you have become more independent or more reliant on external help? Why?

Interviewee: I think I've become more independent. The AI is more like my vocabulary database or an intelligent proofreader, not a replacement for me. It saves me a lot of time that I would otherwise spend looking up words. It allows me to focus more on content editing and logical organization.

Interviewer: When interacting with AI, how do you decide what questions to ask and what instructions to input?

Interviewee: I adjust the process at different stages based on the writing task. After the first draft is completed, the instructions become more specific, and then it's roughly the same. This helps me open up my thinking faster and doesn't limit my creativity.

Interviewer: Has using AI made you more responsible for your English learning or writing? Do you have any specific examples to share?

Interviewee: Yes, before, when I did English homework, I felt like I was done once it was finished. But after using AI, I actively compare the differences before and after revisions. I analyze why I didn't think of such expressions, and I also write down the mistakes I often make in my notebook. This way, I realize that many of my frequently made mistakes have significantly decreased. This proactive awareness is a manifestation of responsibility.

Interviewer: Would you be willing to continue using AI as a self-study tool for English writing in the future? If you're willing, how do you plan to use it?

Interviewee: Of course. Will you use it more specifically in the future? Firstly, to improve my weaknesses; secondly, when writing professional English articles. When writing English articles, I'll adapt it to the styles of different media. Thirdly, I'll try using AI for writing feedback and review, helping me to develop subsequent learning plans.

Interviewer: In the process of using AI, has it inspired you to think more about the content or language of your writing? Do you feel that it makes you reflect more on the expression of content, or does it just stay at the language level?

Interviewee: I feel that it has greatly inspired my thinking, and more importantly, I've reflected more on the expression of content. For example, when I wrote an article about the impact of AI on the news industry, AI could supplement it during the polishing process; it mentioned that it could add specific cases to support the viewpoints. This makes me actively look for relevant cases to make the article more persuasive. This is a deeper understanding of the content from the language level.

Interviewer: So, do you think AI helps or hinders your original viewpoints in writing?

Interviewee: It helps. AI doesn't generate viewpoints for me; it just suggests ways to refine them based on my existing ones. It also recommends expansion methods, allowing me to improve my viewpoints from more angles, rather than being limited to my own understanding.

Interviewer: Overall, has your understanding of writing itself changed through your experience with AI writing?

Interviewee: Yes, it has. I remember when I was in high school, I thought writing was just about recording what I learned in class, and translation was just about copying down. It felt like rote memorization. But in college, AI made me realize that good writing requires not only fluent language but also clear logic, solid content, and a suitable style. It's not something that can be done in one go.

Interviewer: Finally, besides what we've discussed, is there anything else you'd like to share about how AI has affected your writing, learning, or thinking?

Interviewee: I think the greatest value of AI is that it reduces the cost of English writing, allowing me to express myself boldly.
