# Supplementary material for: Is Gen AI an editor or a revision tool? A case study of EFL students in AI-assisted revision
Source: PLoS One. 2026 Oct 29;21(10):e0359661. doi: 10.1371/journal.pone.0359661 (PMC13718921; doi:10.1371/journal.pone.0359661)
Supplement: Supplementary file 2 [file pone.0359661.s002.zip › Interview data/15.docx]

Interviewer: Next, we will discuss issues related to AI-assisted English writing.

Interviewer: You can share more details based on your own experience.

Interviewer: First question, in what aspects of your daily activities do you use AI?

Interviewer: In general?

Interviewer: In English learning?

Interviewer: And English writing?

Interviewee: I use AI quite frequently, about 2-3 times a day, mainly to collect news topic materials and organize interview outlines.

Interviewee: Outlines.

Interviewee: I extract the core information from trending events and occasionally use it to plan my studies and internships.

Interviewee: I use it even more frequently in English learning; I use it almost every day.

Interviewer: Could you describe how you used AI to polish your writing in a particular writing assignment?

Interviewee: The most memorable example is probably the English commentary on the impact of social media on news dissemination that we were required to write in the last course.

Interviewee: An argument.

Interviewee: I first built a framework, then wrote down the core arguments and simple examples I could think of.

Interviewee: But many sentences in junior and senior high school textbooks are simple sentences, and the vocabulary is very basic.

Interviewee: For example, the repeated use of "good" and "bad" to describe the impact, and the logical connections are very stiff; even I can't read it smoothly.

Interviewee:

Interviewer: What types of feedback from AI are most helpful to you?

Interviewer: For example, grammar correction, vocabulary replacement, sentence restructuring, and language style adjustment.

Interviewee: Grammar correction and vocabulary substitution helped me the most.

Interviewee: Because I'm gay, my English foundation isn't very strong.

Interviewee: Good.

Interviewee: I often make mistakes like tense confusion and subject-verb disagreement when writing essays.

Interviewee: Wrong.

Interviewee: For example, if I mix up the present simple tense and the present perfect tense, the AI will directly mark it and correct it, and even briefly explain the reason, which helps me remember.

Interviewer: When using the AI, do you completely accept its suggestions, or do you sometimes reject or modify them?

Interviewer: Can you give an example to explain why?

Interviewee: Mostly I partially accept them, but occasionally I reject them.

Interviewee: Because some of the AI's modifications are too complex for me to understand, and they don't quite match my expression abilities.

Interviewee: For example, in my last English news analysis on the responsibility of mainstream media in public emergencies, I mentioned that machine stream models should tell the truth clearly. I changed it to "machine stream models are obligated to Discuss the vacancy in the context of emergencies." I stared at it.

Interviewee: I stared at this revised version for a long time, and I didn't recognize many of the words.

Interviewee: Yes.

Interviewee: And I found it too convoluted.

Interviewee: News writing should be easy to understand at a glance.

Interviewer: After using AI editing, are you more aware of where you tend to make mistakes, or where your language expression needs improvement?

Interviewee: Very clear.

Interviewee: Yes.

Interviewee: Before, I had no idea I had so many grammatical errors.

Interviewee: For example, the use of articles "a" and "the," I often omit or misuse them.

Interviewee: When the AI was proofreading, it told me this was specific, referring to a particular report, and also pointed out prepositional phrases.

Interviewer: After the proofreading, did you feel the final version more accurately expressed your writing intent?

Interviewer: The image?

Interviewee: Yes, it's significantly more accurate, and others can understand it too.

Interviewee: Yes.

Interviewee: For example, last time I wrote about the impact of algorithmic recommendations on news consumption.

Interviewee: I originally wanted to express that algorithmic recommendations make it easier for users to see similar news.

Interviewee: News.

Interviewee: It's difficult to access different viewpoints.

Interviewee: But the first draft was a mess.

Interviewee: After access, AI proofreading, and my adjustments, it corrected grammatical errors and accurately expressed my core ideas.

Interviewee: Law.

Interviewee: It was much easier than my own first draft.

Interviewer: When you see AI editing, do you accept it completely, partially, or not at all?

Interviewer: How do you decide whether to adopt it?

Interviewee: I mainly accept it partially.

Interviewee: Yes.

Interviewee: My criteria are simple. First, can I understand it?

Interviewee: Do you know how to use it?

Interviewee: If the AI editing is too complex and exceeds my English level, I won't adopt it.

Interviewee: Second, does it deviate from my writing intention?

Interviewee: If the meaning changes after editing, I will adjust it.

Interviewer: Have you ever felt that using AI tools made you more independent in the writing process, or more reliant on external help?

Interviewer: Why?

Interviewee: I think it's both somewhat reliant and gradually becoming more independent.

Interviewee: Independent.

Interviewee: The reliance is because my English foundation is weak; without AI, I can't even find grammatical errors.

Interviewee: The articles I write are incomprehensible to most, but at the same time, I'm becoming more independent.

Interviewer: Because the AI tells me why it should be revised this way. For example, when interacting with the AI, how do you decide what questions to ask it?

Interviewer: Or what kind of instructions do you input?

Interviewee: I ask questions based on my weaknesses.

Interviewee: First, I clarify the type of writing task, such as whether it's a news release or a commentary.

Interviewee: An article.

Interviewee: Then I input instructions based on my own problems.

Interviewee: For example, when writing a breaking news report in English, I input, "Help me correct the grammatical errors in this news report."

Interviewee: Simplify complex sentences.

Interviewer: Does using AI for editing make you feel more responsible for your English learning or writing?

Interviewer: Do you have any specific examples to share?

Interviewee: Yes, it definitely makes me feel more responsible.

Interviewee: Sense of responsibility.

Interviewee: Before, when I did English assignments, I would just scribble a few sentences and hand them in, not caring at all about whether they were right or wrong.

Interviewee: But after using AI, seeing all the grammatical errors it pointed out and the suggestions for improvement...

Interviewee: I realized how bad my English was, and I felt I couldn't keep being so careless.

Interviewer: Would you be willing to use AI as a self-study tool for English writing in the future?

Interviewer: Why?

Interviewer: How?

Interviewee: I would be very willing; it's so useful for someone like me with a weak English foundation.

Interviewee: Yes.

Interviewee: Because AI can help me correct mistakes anytime and give me suggestions.

Interviewee: I don't have to wait for the teacher to grade it; it's very efficient.

Interviewee: And it can adjust the difficulty of the corrections according to my level.

Interviewer: Otherwise, during the AI process, did it stimulate more of your own thinking about the content or language of your writing? Interviewee: Yes, AI not only corrects my language but also points out content issues.

Interviewee: For example, this argument lacks supporting examples, and the logic isn't very smooth.

Interviewee: This makes me rethink my writing.

Interviewee: For example, when I wrote about how short videos improve news dissemination,

Interviewee: I only wrote that short videos make news dissemination faster, and the AI suggested adding a specific example.

Interviewer: Do you feel that using AI has made you reflect more on the content expression of your articles, or is it just staying at the language level?

Interviewer: Could you elaborate?

Interviewee: At first, I mainly stayed at the language level because I was most worried about grammatical errors.

Interviewee: And the writing didn't flow, but gradually I also started to reflect on the content expression.

Interviewee: Yes.

Interviewee: Because in modern literature writing, fluent language alone isn't enough; the reader also needs to understand the core argument.

Interviewee: For example, when I wrote about the challenges of journalism in the digital age.

Interviewee: In my first draft, I mixed up privacy breaches and fake news.

Interviewer: In your opinion, does AI help or hinder you from expressing original viewpoints in your writing?

Interviewee: I think it helps because my English is weak; often I have ideas but can't express them.

Interviewee: I can only write simple and general sentences.

Interviewee: AI can help me express original viewpoints and make them more specific.

Interviewee: Specificity.

Interviewer: Overall, has your understanding of writing itself changed through this experience of using AI-assisted writing?

Interviewee: Quite a big change.

Interviewee: Before, I thought English writing was just about putting words together into sentences, as long as the grammar was correct.

Interviewee: Okay.

Interviewee: Especially since my foundation is weak, I was satisfied just to finish and submit it.

Interviewee: But now I understand that writing is about clearly conveying your thoughts to others.

Interviewee: For journalism majors, English writing also requires enabling readers to quickly grasp the core information.

Interviewer: Besides the above questions, is there anything else you'd like to share about the impact of AI on your writing, learning, or thinking?

Interviewee: I'd like to add that AI not only helped improve my English writing but also gave me more confidence in learning English.

Interviewee: Yes.

Interviewee: Before, my English test scores were low, and I was always afraid of making mistakes when writing essays, so I was particularly resistant to English writing.

Interviewee: But after using AI, I can see my writing gradually improving.

Interviewer: Thank you very much for your detailed sharing. Your experience is very valuable to our research. This interview has now concluded.
