# Supplementary material for: Is Gen AI an editor or a revision tool? A case study of EFL students in AI-assisted revision
Source: PLoS One. 2026 Oct 29;21(10):e0359661. doi: 10.1371/journal.pone.0359661 (PMC13718921; doi:10.1371/journal.pone.0359661)
Supplement: Supplementary file 2 [file pone.0359661.s002.zip › Interview data/16.docx]

Interviewer: Okay, how often do you use AI in your daily activities?

Interviewee: I use AI almost every day, mainly for researching, organizing notes, and polishing text.

Interviewee: I use it quite a lot in English, especially in writing.

Interviewee: Every time I write an English essay, I have AI check for grammatical errors or awkward sentences.

Interviewer: Could you describe how you used AI to polish your writing in a particular writing task?

Interviewer: What?

Interviewee: Once, I was writing an essay about the influence of campus media. I wrote it myself first, then copied it and sent it to AI to check for grammatical errors.

Interviewee: And also to see if it could make the sentences sound more natural.

Interviewee: It helped me correct several long sentences and replaced some repetitive words with more appropriate expressions.

Interviewer: What types of feedback from AI are most helpful to you?

Interviewer: Helpful?

Interviewee: I think the most helpful feedback is sentence structure adjustment and phrase replacement.

Interviewee: Sometimes I feel my sentences are too convoluted, and AI can help me simplify them and remind me which words are not used accurately.

Interviewer: When using AI, do you always accept its suggestions completely, or do you sometimes reject or modify them?

Interviewer: Uh...?

Interviewee: I don't always accept them completely.

Interviewee: Once, it changed a metaphor I wrote into a very ordinary expression. I felt that would lose my own style, so I kept the original sentence.

Interviewee: I only accepted its subsequent grammatical corrections.

Interviewer: After using AI for polishing, are you more aware of where you are more prone to making mistakes, or where your language expression needs improvement?

Interviewee: Yes, I find that I often write sentences that are too long.

Interviewee: Long.

Interviewee: The logic is also prone to being convoluted. It made me realize my original sentence didn’t sound native.

Interviewer: After completing the polishing, do you feel that the final version more accurately expresses your writing intention?

Interviewer: Images?

Interviewee: Most of the time, yes, especially after it helped me clarify the logic, making the article much clearer to read.

Interviewee: But sometimes his revisions are too rigid, making my own voice sound less natural.

Interviewer: When you see AI editing, do you accept it completely, partially, or not at all?

Interviewer: How do you decide whether to accept it or not?

Interviewee: I usually accept it partially. I look at whether the changes make the expression more accurate and natural.

Interviewee: If it looks strange after the revision, I won't accept it.

Interviewee: The criterion is whether it sounds like my own voice.

Interviewer: Do you think using AI tools has made you more independent in the writing process, or more reliant on external help?

Interviewer: Why?

Interviewee: I think I've become more independent.

Interviewee: Yes.

Interviewee: Although it seems like I'm relying on it, it actually makes me clearer about where my writing is weak and teaches me how to revise.

Interviewee: Gradually, I've been able to write more fluent sentences on my own.

Interviewer: When interacting with AI, how do you decide what questions to ask it or what instructions to give it?

Interviewer: Uh...?

Interviewee: I first check my own work to see where it might be lacking, like a sentence that doesn't flow smoothly or a word I'm using poorly.

Interviewee: Then I tell the AI to take a look; I don't just dump everything on it.

Interviewer: Does using AI for editing make you feel more responsible for your English learning or writing?

Interviewer: Do you have any specific examples you can share?

Interviewer: Yes.

Interviewer: Yes.

Interviewee: Before, I would just finish writing an essay and forget about it. Now I repeatedly look at the AI's corrections and think about why those corrections are better.

Interviewee: Once, I specifically copied down the sentences it corrected and compared them with my own. I found the difference was quite significant.

Interviewee: After that, I became more conscious of imitating its expression style.

Interviewer: Would you be willing to use AI as a self-study tool for English writing in the future? Interviewer: Why?

Interviewee: How?

Interviewee: How?

Interviewee: Of course I'd like to. It's like a teacher always online, able to point out my problems.

Interviewee: I plan to have it correct my essays every time I finish writing them, and then write down common mistakes to avoid repeating them.

Interviewer: In using AI, has it inspired you to think more about the content or language of your writing?

Interviewee: Yes, it doesn't just correct the language; sometimes it asks me, "Is this the main point of your expression?"

Interviewee: This makes me rethink what I really want to say.

Interviewee: Writing isn't just about writing correctly; it's also about writing clearly and having a focus.

Interviewer: So, do you think using AI has made you reflect more on the content and expression of your writing, or is it just limited to the language level?

Interviewee: I think it's more about the content level.

Interviewee: The surface.

Interviewee: Although it mainly claims to correct language, once the language was corrected, I realized that what I wanted to express wasn't clear.

Interviewee: For example, once I wrote about the influence of the media on students.

Interviewee: It asked me what kind of influence, positive or negative.

Interviewee: I then realized that my writing was too vague.

Interviewer: In your opinion, does AI help or hinder you from expressing original viewpoints in your writing?

Interviewer: Viewpoints.

Interviewee: I think it helps.

Interviewee: It doesn't write viewpoints for me, but it helps me express my true viewpoints more clearly.

Interviewee: Clearly.

Interviewee: Sometimes my writing is too convoluted, and after it corrects it, I realize that this is actually what I wanted to say.

Interviewer: So, overall, through this experience of using AI-assisted writing, has your understanding of writing itself changed?

Interviewee: Yes.

Interviewee: I used to think writing was simply putting down my thoughts. Now I think writing is not just about producing a grammatically correct sentence; it is a gradual process of developing, revising and refining ideas

Interviewee:

AI has shown me that what I write can be even closer to what I truly want to express.

Interviewer: Besides these issues, is there anything else you'd like to share about the impact of AI on your writing, learning, or thinking methods?

Interviewer: Huh?

Interviewee: I think AI is more than just a tool; it's like a mirror, reflecting the problems in my writing and the flaws in my thinking.

Interviewee: It doesn't write for me, but it makes me clearer about how I should write.

Interviewee: From now on, whether I'm writing in Chinese or English, I will maintain this habit of writing first, then revising, and reflecting as I revise.
