# Supplementary material for: Is Gen AI an editor or a revision tool? A case study of EFL students in AI-assisted revision
Source: PLoS One. 2026 Oct 29;21(10):e0359661. doi: 10.1371/journal.pone.0359661 (PMC13718921; doi:10.1371/journal.pone.0359661)
Supplement: Supplementary file 2 [file pone.0359661.s002.zip › Interview data/17.docx]

**Interviewer:** Great. Let’s start with your everyday use of AI.
How frequently do you use AI tools in your daily life, and for what purposes?
Do you use them often in English learning?

**Participant:** I use AI almost every day. I mainly rely on it to look up information, organise my notes and polish my writing. Because I know AI will fix my writing anyway. In terms of English learning, I also use it quite often, especially when I’m writing. Whenever I write an English composition, I usually ask AI to check whether there are any grammatical mistakes or awkward expressions.

**Interviewer:** Could you describe a specific writing task where you used AI to refine your work?

**Participant:** Sure. I once had to write an essay about the influence of campus media.
I first drafted the essay by myself, then copied the whole text into an AI tool and asked it to check for grammatical issues.
It not only corrected the grammar but also pointed out several unclear sentences that I had not noticed.

**Interviewer:** What kinds of feedback from AI are most helpful to you?

**Participant:** The most useful feedback is usually about sentence structure and word choice.
Sometimes my sentences become unnecessarily long or complicated, and AI helps me simplify them.
It also reminds me when my vocabulary use is not very accurate.

**Interviewer:** When AI provides suggestions, do you accept all of them, or do you selectively decide what to keep?

**Participant:** I never accept everything blindly.
For example, there was once a metaphor I wrote that AI rewrote into a very plain sentence.
I felt it completely changed my tone and personal style, so I kept my original metaphor and only accepted the grammatical corrections.
I usually judge based on whether the revised sentence still sounds like something I would naturally say.

**Interviewer:** After using AI to polish your writing, do you feel more aware of your own weaknesses or recurring errors?

**Participant:** Yes, definitely.
I realised the sentence wasn’t smooth, they tend to be too long and sometimes my logic becomes confusing. It feels easier to rewrite now because I can see more possibilities. This helps me understand which areas I need to improve.

**Interviewer:** Do you feel that the final version, after AI assistance, conveys your writing intention more clearly?

**Participant:** Most of the time, yes.
The logic becomes clearer and the overall flow improves.
But sometimes AI makes the tone too formal or rigid, which feels less like my own voice.

**Interviewer:** Do you think using AI makes you more independent or more reliant on external help in your writing?

**Participant:** Surprisingly, I think it makes me more independent.
Even though it seems like I’m relying on AI, it actually helps me understand my own problems better.
Over time, I’ve learned how to revise my sentences before even asking AI, because I already know what kinds of issues I tend to have.

**Interviewer:** When interacting with AI, how do you decide what to ask or what instructions to give?

**Participant:** I first look at the parts that feel wrong—for example, a sentence that doesn’t flow smoothly or a word I’m not confident about.
Then I highlight those specific issues to AI instead of giving it the entire text without thinking.
I want AI to help, but I still need to think actively.

**Interviewer:** Does using AI make you feel a stronger sense of responsibility toward your own English learning or writing?

**Participant:** Yes. In the past, I would submit my essays without thinking too much.
Now I carefully review the changes AI makes and reflect on why those changes improve the writing.
Once I even rewrote all the corrected sentences by hand to compare them with my original version—it made me more conscious of my own habits and mistakes.

**Interviewer:** Would you be willing to use AI as a self-learning tool for English writing in the future?

**Participant:** Absolutely. It’s like having an online teacher who is always available.
I plan to use it after every writing task and keep a record of my common errors so I can avoid repeating them.

**Interviewer:** Has interacting with AI encouraged you to think more deeply about your content or language?

**Participant:** Yes. AI doesn’t just correct grammar; sometimes it asks questions like, “Is this your main point?” or “Do you mean positive or negative influence?”
These questions push me to clarify my intentions and strengthen my argument.

**Interviewer:** Do you feel AI helps or hinders your ability to produce original ideas?

**Participant:** I think it helps. AI doesn’t create ideas for me, but it helps me express my original ideas more clearly.
Sometimes my writing is too vague, and once AI reorganises it, I realise what I truly wanted to say.

**Interviewer:** Overall, has your understanding of writing changed after using AI?

**Participant:** Yes, it has.
Previously, I thought writing simply meant putting ideas onto paper.
Now I see that writing is a process of constant revision, gradually getting closer to what you genuinely want to express.
AI has shown me that my writing can always be clearer and more refined.

**Interviewer:** Before we end, is there anything else you would like to share about how AI has influenced your writing, learning, or thinking habits?

**Participant:** I feel that AI is more than a learning tool.
It’s like a mirror that reflects my weaknesses in writing and even in thinking.
It doesn’t replace my work, but it shows me how to improve.
No matter whether I’m writing in Chinese or English, I think I’ll keep the habit of drafting first, revising carefully and reflecting on each revision.
AI has changed the way I learn and think, not just the way I write.
