# Supplementary material for: Is Gen AI an editor or a revision tool? A case study of EFL students in AI-assisted revision
Source: PLoS One. 2026 Oct 29;21(10):e0359661. doi: 10.1371/journal.pone.0359661 (PMC13718921; doi:10.1371/journal.pone.0359661)
Supplement: Supplementary file 2 [file pone.0359661.s002.zip › Interview data/18.docx]

Interviewer: Okay, hello, thank you for participating in this interview.

Interviewer: Next, we will discuss issues related to AI-assisted English writing.

Interviewer: Could you share your experience in more detail?

Interviewer: In your daily life, in what areas do you use AI frequently?

Interviewer: In what areas?

Interviewer: In English learning?

Interviewer: In English writing, etc.

Interviewee: I use AI quite frequently in my daily life, about 1 to 2 times a day, mainly to collect news topics, organize interview materials, and extract the context of hot events.

Interviewee: I also occasionally use it to plan study tasks.

Interviewee: Of course, I use it even more frequently in English learning, almost every day.

Interviewee: Yes. Interviewee: For example, memorizing basic news vocabulary, reading along with English news clips, correcting pronunciation, etc.

Interviewee: Etc.

Interviewer: Okay, could you describe how you used AI to polish your writing in a particular writing assignment?

Interviewee: The most memorable was the last course assignment requiring us to write a short English commentary on teenagers' use of short videos and their news awareness.

Interviewee: I first built a simple framework and wrote down the core ideas.

Interviewee: But the first draft was full of simple sentences, and the vocabulary was very basic, for example, repeatedly using "important" and "bad."

Interviewee: The logical connections were also weak, for example, using "and" and "but" to force paragraphs.

Interviewee: Then I copied the entire article into the AI, inputting instructions to help me correct grammatical errors and use simple words with expressions more suitable for news commentary.

Interviewer: Okay.

Interviewer: Yes.

Interviewer: What types of feedback from the AI were most helpful to you? Interviewer: For example, grammar correction, vocabulary replacement, sentence restructuring, and style adjustment.

Interviewee: Grammar correction and sentence restructuring are the most useful.

Interviewee: My English foundation isn't very strong, and I often make mistakes like tense confusion and subject-verb disagreement in my writing.

Interviewee: For example, mixing up the simple past tense and the present perfect tense.

Interviewee: The AI will directly mark and correct these errors, and also provide a simple explanation of the reasons.

Interviewer: When using the AI, do you completely accept its suggestions, or do you sometimes reject them, or modify them?

Interviewer: Can you give an example to explain why?

Interviewee: I mostly accept them partially, but occasionally I reject them because some of the AI's modifications are too complex; I not only can't understand them, but I also can't use them.

Interviewee: And they might deviate from the concise and easy-to-understand requirements of news writing.

Interviewer: After using the AI for editing, are you more aware of where you tend to make mistakes?

Interviewer: Mistakes?

Interviewer: Or is it that my language expression needs improvement?

Interviewee: It's very clear now. I had no idea I made so many mistakes with articles and prepositions before.

Interviewee: For example, I often forgot to add the definite article "the" before "report".

Interviewee: When I wrote "Pay attention to" as "Pay attention in", the AI would mark it and correct it.

Interviewer: After the AI polishing, did you feel that the final version more accurately expressed your writing intention?

Interviewee: Yes, for example, my own writing was much more accurate, and others could quickly grasp the core meaning.

Interviewee: For example, when I wrote about the impact of algorithmic recommendations on news consumption, I originally wanted to express that algorithmic recommendations allow users to only see news they are interested in.

Interviewee: It's difficult to access different viewpoints.

Interviewee: But the first draft was a mess.

Interviewer: So after seeing the AI polishing...

Interviewer: After...

Interviewer: Would you accept it all, partially, or not at all?

Interviewer: How do you decide whether to accept something or not? Interviewee: I mainly accept it partially.

Interviewee: I have two criteria for judgment. First, can I understand it and use it?

Interviewee: Second, does it deviate from my writing intention and news writing style?

Interviewer: Do you think using AI tools has made you more independent in the writing process?

Interviewer: Yes?

Interviewer: Or have you become more reliant on external help?

Interviewer: Why?

Interviewee: I think I've gradually become more independent while relying on it.

Interviewee: Independent.

Interviewee: I rely on it because without AI, I wouldn't be able to find grammatical errors in my English, and my writing wouldn't be coherent.

Interviewee: But at the same time, AI has also promoted my independence because it tells me why I need to revise.

Interviewer: When interacting with AI, how do you decide what questions to ask it or what instructions to input?

Interviewee: It depends on my writing task and weaknesses.

Interviewee: First, clarify the writing type, such as whether it's a news draft or a commentary article.

Interviewee: Then, input instructions based on my needs. For example, when writing a breaking news report in English, I would input "help me correct grammatical errors in this news report."

Interviewee: Make the sentences concise and easy to understand, ensuring the core elements such as time, place, people, and events are included.

Interviewee: Adhere to the simple and clear style of news writing.

Interviewee: At the same time, I can also directly ask the AI when I encounter sentences I don't know. Many elderly people get local news through community newspapers.

Interviewee: How can I revise this sentence to conform to English news expression?

Interviewee: This way, the AI feedback will be more tailored to my actual needs.

Interviewer: So, during the AI polishing process, does it make you feel more responsible for your English, English learning, or writing?

Interviewer: Yes?

Interviewer: Do you have any specific examples you can share?

Interviewee: Some definitely make me feel more responsible.

Interviewee: Before, when I did English homework, I would just write a few sentences to get it over with, not caring about whether they were right or wrong.

Interviewee: But after using AI, seeing all the errors and suggestions it marked made me realize that my English needed improvement.

Interviewee: I also felt I couldn't be so careless anymore.

Interviewee: Yes, I was.

Interviewer: So, would you be willing to use AI as a self-study tool for English writing in the future?

Interviewer: Why?

Interviewer: How?

Interviewer: How?

Interviewee: Absolutely. It's so useful for someone like me with a weak English foundation.

Interviewee: Yes.

Interviewee: Because AI can help me correct mistakes and give suggestions anytime, without waiting for a teacher to revise, which is very efficient. And it can adjust the difficulty of the corrections, make it more like my level.

Interviewee: First, I'll keep a notebook of my mistakes, recording all the grammatical errors and prepositional phrase errors marked by AI, and review them regularly.

Interviewee: Second, accumulate practical vocabulary.

Interviewee: Use vocabulary.

Interviewee: Memorize the simple yet professional news vocabulary recommended by AI, along with example sentences.

Interviewee: Third, practice specifically, such as practicing English in particular.

Interviewee: Write English leads, and after writing, have the AI evaluate whether it contains core elements and whether the grammar is correct, so you can gradually improve.

Interviewer: In the process of using AI, has it inspired you to think more about your writing content or language?

Interviewee: Yes, the AI not only corrects the language but also points out problems with the content, such as the lack of supporting examples for the argument or the illogical flow.

Interviewee: This makes me rethink the content I write.

Interviewee: For example, regarding the impact of short videos on news dissemination, I only wrote that short videos make news spread faster.

Interviewee: The AI suggested adding a specific example, such as a hot topic spreading across the entire internet within hours through short videos.

Interviewee: I thought of a recent social hot topic to add.

Interviewee: The article suddenly became much more specific.

Interviewer: Do you think using AI made you reflect more on the content expression of your writing, or did it only stay at the language level? Could you elaborate?

Interviewee: At first, it mainly stayed at the language level because I was worried about grammatical errors and incoherence.

Interviewee: But gradually I also started to reflect on the content expression, because in modern literary writing, fluent language alone is not enough.

Interviewee: The core concept also needs to be clear to the reader.

Interviewee: For example, when I wrote about the challenges of news ethics in the digital age, in the first draft, I mixed up privacy breaches and fake news.

Interviewee: When the AI polished it, it not only corrected the grammar but also suggested separating the two topics.

Interviewer: So, in your opinion, does AI help or hinder you from expressing original viewpoints in your writing?

Interviewee: I think it helps, because my English foundation is average. Often, I have thoughts in my mind but can't express them, so I can only write simple, general sentences.

Interviewee: AI can help me express original viewpoints.

Interviewee: It can also make your viewpoints more specific.

Interviewer: Overall, has your understanding of writing itself changed through this experience of using AI-assisted writing?

Interviewee: Changed?

Interviewee: Quite a big change. Before, I thought English writing was just about putting words together into sentences, as long as the grammar was correct.

Interviewee: Especially since my foundation wasn't very strong, I was satisfied just to finish and submit it.

Interviewee: But now I understand that writing is about clearly understanding your thoughts and accurately communicating them to others.

Interviewee: For journalism majors, English writing also needs to ensure readers quickly grasp the core information.

Interviewer: Besides the above questions, is there anything else you'd like to share?

Interviewer: Do you have any thoughts on how AI has affected your writing, learning, or thinking methods?

Interviewer: ?

Interviewee: I'd like to add that AI not only helped me improve my English.

Interviewee: My writing ability has also given me more confidence in learning English.

Interviewee: My confidence.

Interviewee: My English test scores used to be quite low, and I always made mistakes in my essays, so I really disliked writing in English.

Interviewee: But after using AI, I can see my writing gradually improving.

Interviewee: From being full of grammatical errors to becoming fluent and easy to understand, I've slowly stopped being afraid.

Interviewer: Okay, thank you very much for your detailed sharing.

Interviewer: Your experience is very valuable to our research. This interview has now concluded.
