# Supplementary material for: Is Gen AI an editor or a revision tool? A case study of EFL students in AI-assisted revision
Source: PLoS One. 2026 Oct 29;21(10):e0359661. doi: 10.1371/journal.pone.0359661 (PMC13718921; doi:10.1371/journal.pone.0359661)
Supplement: Supplementary file 2 [file pone.0359661.s002.zip › Interview data/2.docx]

**Interviewer:** Let's begin our interview. First, could you share your English score from the college entrance examination?

**Interviewee:** I scored a little over 90 in the college entrance exam.

**Interviewer:** Great. Do you like English as a subject?

**Interviewee:** Compared to what we’re currently studying, I actually enjoy advanced mathematics more.

**Interviewer:** Why is that? Why would you prefer advanced mathematics over English?

**Interviewee:** Umm… do I have to compare? I just feel that English is not something I naturally like. It’s not that I dislike it completely, but my interest is definitely stronger in mathematics.

**Interviewer:** How often do you use AI in your daily life?

**Interviewee:** Quite often. Not just me—many of my classmates also use it frequently.

**Interviewer:** In what kinds of situations do you usually use it?

**Interviewee:** For example, when the teacher asks questions in class. Especially in English class, our teacher requires us to first write an English essay ourselves, then submit two versions. The second version must be revised using AI. So the whole class basically uses it regularly.

**Interviewer:** Besides writing, in other aspects of English learning, are you allowed to use your phone to ask AI?

**Interviewee:** Yes. When the teacher asks questions in class, sometimes she even tells us to ask AI directly. So we use it quite a lot.

**Interviewer:** How about outside class? Do you use AI independently?

**Interviewee:** Yes. For some homework assignments, especially ones that require writing, I might start by asking AI to help me create an outline and then write based on that.

**Interviewer:** When working on your writing assignment, how exactly did you use AI to polish your essay?

**Interviewee:** I usually write the whole piece myself first. After that, I ask the AI to take on a role—like a university English professor—and revise my essay. It helps me check grammar, improve expressions, and make my writing sound more sophisticated.

**Interviewer:** Of all the feedback AI gives you, which parts are most helpful?

**Interviewee:** Mainly the parts I didn’t realise were problems. It can point out hidden grammar mistakes or awkward expressions and offer suggestions to make the writing clearer and more refined.

**Interviewer:** What do you mean when you say “more sophisticated”?

**Interviewee:** It often replaces simple, conversational words with more formal or academic synonyms. The style becomes more complex and polished—more like something written in a formal context rather than casual speech.

**Interviewer:** So AI adjusts grammar, vocabulary, structure, and style?

**Interviewee:** Yes. Its structure is very clear. But when it tries to connect ideas to real-life examples, sometimes the output feels stiff and unnatural—like something a human wouldn’t say.

**Interviewer:** Could you share an example?

**Interviewee:** Yes. Our teacher once asked us to write an essay about success—how to achieve success. When I asked AI to provide examples, the example it generated was very general and didn't fit my own experience. It felt obviously written by AI. I had to revise that part myself.

**Interviewer:** You mean the example didn’t match your real-life context?

**Interviewee:** Exactly. The AI example sounded too standardised. A human could tell immediately that it wasn’t written by a student.

**Interviewer:** Have you ever rejected AI’s suggestions?

**Interviewee:** Yes. If I feel something is inappropriate or unnatural, I ask it to revise again or I simply remove that part.

**Interviewer:** After using AI, do you feel your language skills improved?

**Interviewee:** Yes. It points out all my mistakes very quickly. Grammar, collocations, sentence structure—these become clearer to me. When I review them, I feel my understanding improves and I’m less likely to repeat the same mistakes.

**Interviewer:** For example, in this assignment, I noticed you didn’t adopt some suggestions AI provided. Why?

**Interviewee:** Those sentences were too sophisticated—beyond my current English level. I want my writing to look like something I can naturally produce, not something artificially inflated.

**Interviewer:** So you feel some AI expressions sound fake?

**Interviewee:** Yes. I want to gradually reach that level, but I don’t want my assignment to look unrealistically advanced.

**Interviewee:** Honestly, more reliant. Before, I had to check mistakes myself or ask the teacher. Now AI spots the errors immediately, so I depend on it more.

**Interviewer:** Yet you still learn from the feedback?

**Interviewee:** Yes. I review the corrections, especially the grammar parts, and I try to remember new vocabulary. It does help me improve.

**Interviewer:** What kinds of commands do you think are most effective?

**Interviewee:** The ones our teacher taught us. For example, asking AI to act as a university English professor who reviews my essay and offers revision suggestions.

**Interviewer:** Have you tried other types of prompts?

**Interviewee:** Not yet. I think I should experiment more, but so far I’ve only used the basic ones.

**Interviewer:** Do you feel AI affects your English learning—positively or negatively?

**Interviewee:** Both. It helps me learn new sentence structures and vocabulary, but it also makes me overly dependent. Sometimes it reduces the time I spend thinking independently.

**Interviewer:** Does it give you inspiration for writing?

**Interviewee:** Yes. Sometimes I ask it for a simple outline to brainstorm ideas. Its structure is usually clear, but I still write the content myself.

**Interviewer:** Does AI make you reflect on the deeper content of your essay?

**Interviewee:** Not really. It focuses more on language and structure. It can’t tell if I’m off-topic. So I still need to manage the content myself.

**Interviewer:** Have you used AI in translation tasks?

**Interviewee:** Yes. When I translate Chinese to English or English to Chinese, I ask AI to check my accuracy. Its vocabulary is more advanced than mine, and it gives me useful alternatives.

**Interviewer:** Do you actually learn from the vocabulary it suggests?

**Interviewee:** I try to. I write down the new words and review them.

**Interviewer:** Has AI changed your understanding of the writing process?

**Interviewee:** Yes. Before AI, I had to outline everything and identify problems myself. Now the process is much faster—I don’t need to spend so much time checking errors because AI handles that part.

**Interviewer:** But AI sometimes ignores emotional aspects?

**Interviewee:** Yes. For example, rhetorical devices—when AI translates or rewrites them, they lose the original emotional nuance or beauty.

**Interviewer:** Compared to peer evaluation you experienced in middle school, what do you think of AI?

**Interviewee:** AI is definitely more efficient and accurate. But Even though it was accurate, it didn’t fit my writing. Peer comments sometimes captured emotions or personal perspectives that AI cannot.

**Interviewer:** Do you have anything to add about AI and your writing?

**Interviewee:** Not much. Basically what we’ve discussed covers my experiences.

**Interviewer:** Okay, thank you for participating. This concludes our interview.

**Interviewee:** Thank you, teacher. I’ll call the next student in.
