# Supplementary material for: Is Gen AI an editor or a revision tool? A case study of EFL students in AI-assisted revision
Source: PLoS One. 2026 Oct 29;21(10):e0359661. doi: 10.1371/journal.pone.0359661 (PMC13718921; doi:10.1371/journal.pone.0359661)
Supplement: Supplementary file 2 [file pone.0359661.s002.zip › Interview data/3.docx]

interviewer: This interview is about the relationship between AI and English learning and writing development. It's a research project exploring how AI can help you improve your writing skills without affecting your grades. And Teacher AI won't know the content of our interview. Yeah.

interviewee: It's okay, Teacher.

interviewer : Just tell the truth. Let me know your English score from the college entrance exam.

interviewee: I think it was around 70 points!

interviewer: Do you like English?

interviewee: Not really, for example...

interviewer: How often do you use AI in your daily life?

interviewee: For our major courses, we use ethnology. For example, if I don't understand a part, I'll use AI to help me read it, or for some easy assignments, like writing 1000 words or something. Normally, you wouldn't use AI for this sentence?

interviewer: How often do you use AI in your English learning?

interviewee: Not much. When I use English, I generally only use one app “English speak fluent”, which is for memorizing vocabulary. I also use AI, which is for memorizing vocabulary. I've hardly used AI at all.

interviewer: Actually, you're also a beginner in writing. Are you trying to use it following the teacher's instructions?

interviewee: Yes, just like in high school. I just wrote whatever the teacher told me to write. Generally speaking, if the teacher doesn't require it, I don't need to use it, and I won't use AI to correct anything.

interviewer: How did you use AI in this writing assignment? interviewee (00:06:06): I made some requests and first deleted my article, asking the AI to be lenient. I gave the AI a qualifier for some requests. That's how I did it.

interviewer: So how did you use it to polish it?

interviewee: For example, I would tell it, "I require you to read the whole article. After you finish the whole paragraph, I ask you to correct some grammatical errors and sentence errors. Then, for example, I ask you to replace some of my writing with more advanced vocabulary, like... I also ask you to add some sentences and see if you can optimize the sentence order."

interviewer: Do you think the feedback it provides is helpful to you?

interviewee: I feel that if I really studied or prepared properly... Speaking of the CET-4/6 exams, I took the CET-4 myself.

interviewee: If it's really like that, I feel it's very useful. Very useful, at least.

interviewer: But...

interviewee: For example, if you want to ask a teacher or go to university, I guess it's okay in high school, but in university... Teachers have many students to deal with, and it's impossible for them to give you an immediate answer. For example, if you ask a question, they might not be able to answer immediately, or they might say you'll have to wait a while, or... I feel that if you do that, you should first ask AIIA to correct some errors.

interviewee: I feel it's quite convenient, and you don't have to bother the teacher or anything.

interviewer: So, since you're doing this now, have you done it?

interviewee: I haven't done it.

interviewer: So why didn't you do that?

interviewee: Firstly, my focus isn't really on English right now. I occasionally memorize vocabulary. My overall approach to English learning is just memorizing vocabulary. Even in high school, my overall learning has been primarily vocabulary memorization. I haven't studied much else, except perhaps grammar. In high school, I did a lot of practice questions. In university, besides the assignments given by teachers, I don't actively create my own practice questions. That's the situation.

interviewee : Okay!

interviewer : In this usage, did you fully accept his suggestions? Did you reject any of his suggestions?

interviewee: There are some things that still need to be verified, such as adding multiple AIs, like asking multiple questions together.

interviewer: Which of his suggestions did you adopt, and which did you not? interviewee (00:08:49): It's not just the simplest grammatical errors. I feel AI wouldn't make those kinds of basic mistakes. For example, word order, I feel. AI still can't be like a human. Emotion. I feel it can't handle word order like a human. It can think, but it can't act like a human.

interviewee: To be honest, I can't give many examples because the AI's editing function is almost negligible.

interviewer: Can you give a specific example?

interviewee: I rarely use it. I remember the links were all like that. I forgot exactly how I did it. But I remember clearly that I didn’t take that suggestion from AI.

interviewer : After using AI editing, do you feel you can clearly identify where you are prone to making mistakes?

interviewee : Actually, it's very simple. I feel it's normal for someone at my level. For example, I think my vocabulary is okay, but... I feel I have a common problem: when writing, I can't think of advanced words, or I can only think of the simplest ones. Sometimes the AI changes the sentence too much, so I keep my original version because it sounds more like what I want to say

interviewee : The AI can... the best feeling is that it can replace some things for me, for example... some sentence errors, I can write some advanced ones. For example, "but... no," for example, some openings, etc. I feel it always writes better than I do. It copies a bit, and I feel it really follows what I say.

interviewer: Do you think your language has improved after using it?

interviewee: If I were to plan ahead, I would really study English well to prepare. Actually, if I use it for a long time, I feel it should have some impact, that is, on the way I revise my writing.

interviewer : So in which specific areas do you think it will have a significant impact?

interviewer: In which aspects of writing might it have a greater impact?

interviewee: In which aspects will it have a greater impact? I think it will. First, I think the structure of AIA I is quite formulaic, right? I feel that if you use the formula well, it feels quite good. You need to be good at using the formula. R, I feel, is more formulaic. You will be able to handle it better. It provides some formula questions, if you use it well. This is the biggest change in the problem.

interviewee: Secondly, there is grammar correction. I think grammar correction is also very important. To be honest, I only know a few important clauses. The simple grammar is too detailed, I don't understand it very well. It's not that I don't understand it very well, it's that I haven't studied it much. You don't have to deliberately memorize it, but if you really use it a lot, you can see it. You often see, for example, you write one article a day and finish it? 1000 revisions. For example, you might make the same mistake the first time, and the second time, but the more you revise, the less likely you are to make the same mistake, because you are seeing the revisions it makes for you every day. I didn’t pay attention to the grammar at all at the time. The AI I'm using now is that AI. For example, it will point out the parts it corrects for you, in the simplest way. It also has a certain ability to summarize. It actually saves you something, it saves you the time of summarizing yourself. But. If you want to pursue a certain level of perfection, you still need to summarize it yourself again. Or compare your summary with the AI's. I feel that this is also a kind of progress.

interviewee: For example, you first summarize it yourself, then use the AI to summarize your article and compare it with your own summary. See where you went wrong. For example, you listed six points, and the AI also summarized and corrected six points for you. I feel that this is a kind of progress, you can see where you wrote wrong.

interviewer: What do you think about the final version? After the AIgenerates the text, does it accurately express your writing intention?

interviewee: Regarding writing intention, I feel that firstly, it will reflect your own writing. You must clearly express the qualifiers you give to AI (V) to I (I), for example, the requirement. You should revise this article for me, not automatically, or according to the logic of the text itself. Don't overdo it, for example, by participating online. There's that online search engine, right? You just want it to avoid adding useless words or "experts." You should search first. It should only modify based on the original text, replacing advanced vocabulary. Grammar modification is like that. This way, it will be based on your ability, so I (A) is actually based on your own usage, or the qualifiers you use. How do you feed it?

interviewer: So, do you feel that in the process of using AI to assist your writing, you become more independent? Or more dependent on it?

interviewee : To be honest, I don't really rely on it! In high school, I almost never used it. Not for skincare, I never used it at all. I can say it has a rebound effect, but for my major courses, I sometimes use it. I feel like... Only a small amount remains.

interviewer : If you rely on it, do you think it's a good thing or a bad thing?

interviewee : It depends on the individual. Even if you think of it first, whether you use it or not is up to you. Don't blame your own problems on a machine.

interviewee : I feel like that.

interviewer: Can you give an example of how you rely on it? For example, what would you use AI for?

interviewee : Some simple questions, like answering a question, I might ask...

interviewer: So, when using AI, what kind of instructions do you think would be more effective?

interviewee): I'll just pick one and look at it. Wait a minute. Please wait a moment.

interviewer : It's okay.

interviewee: For example, I might give it a passage, or I might ask it to...

interviewer: It's not tasty.

interviewee: For example, I would give it a qualifier, something like, "from what perspective." This is a qualifier, first giving it a certain perspective, for example, "from what perspective."

interviewee: What subject, or what perspective of an undergraduate or graduate student? After that, give it some logical reasoning and clarity. Then, but... what do you ask of it? It doesn't actually want... it doesn't want to over-interpret the text, for example, this kind of thing. Some limited quantities.

interviewer : Understood! So, do you think you'll be more responsible for your writing when using AI? Are you saying you're handing over responsibility to AI?

interviewee : Impossible. It's not like the AI writes for me and I don't write anything myself. It mainly depends on the individual. Personal problems are the individual's fault. You can't blame it on your own poor learning or writing skills.

interviewee: It's entirely based on your own thoughts, not on...

interviewee: For example, this time... For example, a semester has passed, and it's time for the final exams. Or, a semester has passed, and you're about to take the CET-4 or CET-6. Because it's also around the time of the final exams, it's all because I didn't think about it at all. You think it's because you used it without being told to use it or anything?

interviewee : So, whether it's practice or writing, I think the AIplays a larger role due to my own abilities.

interviewer : That's right. Do you think you'd be willing to use AI as a self-study tool for writing in the future?

interviewee : The original intention of AI is actually to provide an auxiliary tool. That's my feeling.

interviewee: Just focusing on writing, don't give me what I want. The point I mentioned above, the one he gave me for revision, is very beneficial for me. To be honest, I still need to ask my teacher questions. For example, if you send a teacher a question on a weekday, I don't particularly like bothering them. Asking questions is definitely more convenient and beneficial, and I prefer using it.

interviewee: In the future, for example, if I'm learning English from a beginner level, or preparing for an exam for one or two months, I'll be studying English continuously for one or two months.

interviewee: We will continue to use AS.

interviewer: Since you mentioned that AI might help you learn and give you feedback, you must have received peer feedback in middle school. Between these two, which would you prefer, and what are their advantages and disadvantages?

interviewee: Teacher, could you ask me again? I haven't caught up yet.

interviewer : So, in middle school, didn't you use peer evaluation, like your deskmate evaluating your writing? Do you think AI, as a peer, could help you learn?

Would Xi be a good partner?

interviewee : He should be able to explain things to me. In high school, I was always second to last, to be honest, my deskmate was last. We didn't really have any English writing partners.

interviewee: It's unfair.

interviewer: For example, your teachers' evaluations could also be helpful.

Xuanz : None of my teachers took me seriously at all, not even the English class monitor or the English teacher in high school.

interviewer: Actually, AI would be a better partner for you.

interviewee: In high school, I felt that the only English teacher I was close to was the one who tutored me.

interviewer: I understand!

interviewee: We have a pretty good relationship, if I had to say. Throughout my high school English classes, I feel like I don't always compare myself to our teacher, unless, for example, during a few exams, we'll take time to do practice tests and review. I'll do it myself, like doing practice tests or doing exercises on my own, because the teacher doesn't really say anything. Anyway, I feel like I have my own issues with my high school English teacher, and I guess I have a lot of problems myself.

interviewer: I understand!

interviewee: After all, the impression I left from my first year of high school was that my English was just average.

interviewer: Actually, because it's a two-way street, it's impossible to evaluate. So what is your own understanding of writing?

interviewee: My English essay writing is actually something I learned quickly. In my first two years of high school, I only managed to score around 80 points on mock exams in my senior year. I was incredibly diligent in my first two years because of something I learned in my senior year – the kind of thing I call a "hero" – my teacher gave me a few templates, and I relied on memorizing them to write English essays.

interviewer : We're not talking about English essays, we're talking about writing in general.

interviewee: Just talking about it, right?

interviewer: Just speaking plainly, what does the concept of writing in both Chinese and English mean to you?

interviewee): It's about expressing yourself. I feel that writing is very subjective, unless it's historical writing. Generally speaking, even historical writing tends to be subjective, but some requirements, like those in our major, such as observations, field investigations, and field research, are necessary. A continuation is certain, although there's a degree of subjectivity involved, but the possibility must be maintained. This will be rewritten and re-asked based on the specific conditions at the time.

interviewer: So what do you think?

interviewee: I feel everything depends on subjectivity, because humans are driven by feelings, not just emotions, but by emotions. They are social beings; it's impossible for someone to be completely devoid of emotion, like a robot—that would make them all robots.

interviewer: If you use AI as a writing aid, do you think it changes your understanding of writing?

interviewee : I feel AI’s writing style doesn’t sound like my writing anymore.. It will only give me what I want; it can only be me. Perhaps I'll adjust my rhythm and style based on its feedback, but I won't completely become like its medicine. When I notice that something sounds wrong in the AI suggestion, I usually change it right away instead of leaving it as it is.

interviewer: So, regarding the relationship between AI and English writing, is there anything you'd like to add?

interviewee : The only thing I want to say is that AI is just an auxiliary tool. Whether you use it well or not depends on the individual. It's not a one-size-fits-all approach. Like I said, if you use it well, it's a good tool; if you don't, generally speaking, you can't use it alone. It's all your own problem.

interviewer : Okay, that concludes my interview. No further questions.

interviewee: Okay, thank you, teacher!

interviewer : Thank you!

interviewer: Thank you, and have a nice day!

interviewee: Okay.

interviewer: Bye!

interviewee: Okay, bye-bye, teacher!
