# Supplementary material for: Is Gen AI an editor or a revision tool? A case study of EFL students in AI-assisted revision
Source: PLoS One. 2026 Oct 29;21(10):e0359661. doi: 10.1371/journal.pone.0359661 (PMC13718921; doi:10.1371/journal.pone.0359661)
Supplement: Supplementary file 2 [file pone.0359661.s002.zip › Interview data/4.docx]

Interviewer: The interview is about a study on whether AI can assist you in English writing. Our conversation will not be known to Teacher Ai or anyone else, so just tell the truth.

Interviewee: Okay, okay!

Interviewer : I'd like to know your English score in the college entrance exam first.

Interviewee : I scored over 60 in English in the college entrance exam.

Interviewer: Okay, so how often do you use AI in your daily life?

Interviewee : Okay! I've been using AI a lot since I started university.

Interviewer : In what areas do you usually use it?

Interviewee : Usually, it generates some. Article summaries, like reading notes?

Interviewer : Do you directly have it generate reading notes, or do you write them yourself first and then have it revise and polish them?

Interviewee: I usually do it myself first. I write out the outline and the key points I need. Then I give the AI instructions on what role it should play. Then it generates and polishes a reading note for me.

Interviewer : Okay, so how often do you use AI in your English learning process?

Interviewee : Not very often.

Interviewee : Generally, in English learning. For vocabulary and such, in class. And for essays, maybe. Teacher Ai requires us to write one ourselves and then submit it to AI to generate one.

Interviewer : So how did you use AI to polish your writing in this assignment?

Interviewee : First, I wrote an article based on my own ideas. Then I submitted it to Deep Sick for him to revise it, pointing out key points and problems. He then listed the issues in the essay, and I revised it again based on those issues.

Interviewer: Which of the feedback he provided was the most helpful?

Interviewee: Grammar and related issues.

Interviewer: Anything specific?

Interviewee: Sentence structure?

Interviewer : Did you accept all the feedback he provided regarding sentence structure? So you said there were things you refused to do?

Interviewee: Yes, there were things I refused to do. My requirement for him was that he be a university English teacher with a level four proficiency to evaluate some sentences in my essay. I couldn't understand the sentence structure either. My level wasn't high enough, so I...

Interviewer: I understand. So after using it, do you feel that you are more likely to make mistakes in certain areas?

Interviewee: I selectively ignore the more advanced parts. Very advanced ones.

Interviewer : In which areas?

Interviewee: Like some... basic sentence connections, connections between contexts. Also... a lot of spelling problems in this essay.

Interviewer : Do you feel that you have improved in language expression?

Interviewee: Somewhat.

Interviewer: So what exactly is it?

Interviewee: Well, it's just that... Previously, writing text felt very awkward, but now... Now my sentence structure and language flow smoothly. It's a huge improvement. At least it doesn't feel so awkward when written.

Interviewer : I understand. After using it, do you feel that the final version reflects your initial writing intention?

Interviewee: No. With relatively complete instructions, the final version is what I wanted. If I only give some general instructions, the final text... the general meaning will change a lot.

Interviewer: Do you feel that when using AI in the process of assisted writing, you become more independent? Or more reliant on AI?

Interviewee: How so? Is it a tendency to rely on AI? Every time, I unconsciously finish writing and send it to them, then have them generate it several times in a row?

Interviewer: After it's generated, do you check it again?

Interviewee: Yes, I compare it with my original text.

Interviewer: Do you think this method will ultimately affect your writing score, for example, your writing score in the CET-4/6 exam? Will it improve it?

Interviewee : It will improve it somewhat, but not by much, because I rely heavily on AI for sentence structure modification.

Interviewer : Actually, you probably still don't know how you should write it yourself?

Interviewee: Right!

Interviewer: I understand. So, do you think you take responsibility for your own writing during the AIpolishing process? Did you entrust the responsibility to AI?

Interviewee: I think I'm quite responsible, because it will be graded. So I'll look at it again, and then... I'll look at the article it generates every time, and then revise it based on my own understanding.

Interviewer: If you were to use AI as a tool for self-studying English writing in the future, would you be willing?

Interviewee: I would be willing; it's quite convenient.

Interviewer : What aspects do you think you should focus on when self-studying?

Interviewee: One. Vocabulary expression. And sentence structure, etc.

Interviewer : Do you feel he inspired you to think more deeply about the content of your writing?

Interviewee: Well, yes, but not much.

Interviewer : How are you referring to whether it inspired you or not?

Interviewee : Yes, in the very last paragraph, he elevates my article. It makes me... maybe like Interviewee, someone I couldn't express. Not much. The final expression he gives is more formulaic. He does change the main point I want to make in my article.

Interviewer: Do you think AI helps or hinders you from expressing your own original viewpoints when writing?

Interviewee : It's both helpful and hindering, right?

Interviewer : Could you explain that separately?

Interviewee: As for assistance, it will, to a certain extent, remind me. Right now, I am an AI's highly developed expressive controller. I need to use my own ideas to guide this article. And then hinder it. Sometimes I might have a sense of responsibility towards this article, but it might not be enough, my willpower might not be strong enough. Then... then the AIwill guide me.

Interviewee: Think about it from the perspective of viewpoints.

Interviewer: Do you think the viewpoints it provides are acceptable?

Interviewee : It's also a matter of perspective; sometimes it's not acceptable.

Interviewer: Or are you looking at this critically?

Interviewee: Yes!

Interviewer : What is your understanding of writing, not English writing, just the act of writing itself?

Interviewee : This word means expressing the main points based on what I'm thinking or feeling in my mind.

Interviewee: To better present it to others.

Interviewer : Do you think your understanding of this concept has changed when AI assists you in English writing?

Interviewee : No. It seems not.

Interviewer : Do you think it has affected your way of thinking?

Interviewee : To some extent, it does have an impact. It does affect my way of thinking, as I just said, it's deliberate. I consciously follow the AI's viewpoint in that direction. I focus on it and think about it.

Interviewer: Because in middle school, you must have had peer or teacher evaluations, so you probably exchanged writing with your deskmate or classmates for feedback?

Interviewee: Yes!

Interviewer : Do you think AI would be a perfect partner?

Interviewee: No.

Interviewer : Why?

Interviewee: Because it's AI, after all, generated by a bunch of code. What it expresses is very complex. It's very different from my current competitive level compared to AI. I'm speaking from my own human perspective, my emotions.
