# Supplementary material for: Is Gen AI an editor or a revision tool? A case study of EFL students in AI-assisted revision
Source: PLoS One. 2026 Oct 29;21(10):e0359661. doi: 10.1371/journal.pone.0359661 (PMC13718921; doi:10.1371/journal.pone.0359661)
Supplement: Supplementary file 2 [file pone.0359661.s002.zip › Interview data/5.docx]

Interviewer : Okay! I'll find your article first. Okay, let me find out your English score on the college entrance exam first!

Interviewee : Okay, I scored 130 points on the college entrance exam. I think it was 6?

Interviewer : Such a high score, but does Hebei have a listening comprehension section?

Interviewee : Because I'm from Inner Mongolia, our questions are probably easier compared to others, since we have the old college entrance exam, there's no listening comprehension section.

Interviewer : Hebei does. Hebei does, okay. Okay, this interview is about the relationship between AI and the development of English learners' writing. I want to explore whether AI can assist your English writing. First, I'd like to know how often you use AI in your daily life.

Interviewee : I use AI almost every day in my daily life.

Interviewer: In what areas do you use it?

Interviewee : Besides academic questions, I also use it because I've been trying to lose weight recently. For example, when calculating the calories of a food, the energy chart usually says to calculate it using fiber, but I usually ask AI to directly convert it into calories, which is very convenient.

Interviewer: Do you use AI a lot in your English learning?

Interviewee: I don't actually use it that much in my English learning.

Interviewer: You only used it because Teacher Zhang asked you to use it in class.

Interviewee: Yes, I initially wrote an article myself and then gave instructions to the AI.

Interviewer: Could you describe how you used AI to polish your article in this writing task?

Interviewee: I first converted my article into an electronic version, then copied it to the AI. My instructions were to have it polish it, making the article as sophisticated as possible, but not too complex. It also indicated the parts it improved.

Interviewer: I saw your instructions. One point was to make it more sophisticated. I'd like to understand what "more sophisticated" means.

Interviewee: Yes! More sophisticated. To make some of my vocabulary less...

Interviewer: Can you hear me?

Interviewee: I can hear you, teacher. Are you having trouble? I'm here. Haha, teacher!

Interviewer: Okay.

Interviewee: I was a little slow just now.

Interviewer: Okay, okay, it's alright. So, what do you mean by "more advanced"?

Interviewee: Maybe some of my vocabulary is relatively simple, and I want him to use some more advanced vocabulary to express what I mean. Also, I want him to add some more advanced phrases or clauses to some of my simple sentences.

Interviewer: After he finishes expressing it, are you satisfied with his expression?

Interviewee: I'm quite satisfied because I initially used Dick's software instead of Doubao for conversion. After DC converted it, the article felt a bit too advanced for me; some of the vocabulary and phrases were unfamiliar. So I tried using AI and Doubao again to test it. The resulting essay, after polishing, was relatively more advanced, but I feel it's still within my capabilities.

Interviewer: You might use these in the future. Do you think you've used these advanced expressions in your subsequent studies?

Interviewee: Yes!

Interviewee : I haven't specifically encountered them in my subsequent studies.

Interviewer: You think you might think of using these advanced expressions, right?

Interviewee: Yes, is this still about this writing task?

Interviewer: I understand? Among the feedback provided by the AI, which do you think is the most useful and helpful for you?

Interviewer : Yes, not in the entire process of using AI?

Interviewee: Throughout the process of using AI, I felt that the information it provided was very concise, allowing me to see the answers I wanted. Before using AI, we usually used Baidu or browsers to search for information. But when I use it, for example, if I want to convert calories, Baidu might list a bunch of references, such as what calories a thousand peppers represent. If I use an AI like Adoubao, it directly gives me an answer that I can see at a glance. I feel this is very helpful to me.

Interviewer: In English writing?

Interviewee: In English writing. I feel the AI's suggestions for revision were the biggest help. It saved me from having to do very detailed comparisons. It gave me suggestions that I could modify.

Interviewer: Did you ask the AI for ideas?

Interviewer: When you were writing. It was before you even had a first draft.

Interviewee: Not for this particular writing assignment, because the teacher gave us a process to think about in class.

Interviewer: When using this AI in English writing, but not limited to this assignment, did you completely accept its suggestions or did you reject some of them?

Interviewee: Actually, there were some things I refused to use. Even with Doubao's help, I still didn't use some of the more advanced expressions I hadn't seen before. I had to leave some out.

Interviewer : You feel it might not match your current level.

Interviewee: Yes!

Interviewer: Anything else? Where did you refuse to use it?

Interviewee : Other things? Also, I mentioned it to Doubao before, and gave him some instructions regarding writing. His writing was a bit too simple, like a primary school student's level, so I refused to let him use it and asked him to make it more advanced.

Interviewer: I understand. Do you feel that after using AI editing, you are able to clearly recognize where you are prone to making mistakes?

Interviewee : Looking at the essay he polished for me, I can clearly see that my vocabulary might not be strong enough.

Interviewer : Yes, I noticed that almost everything in your essay was about vocabulary, changing it into more advanced expressions.

Interviewee : Right!

Interviewer: This shows that your English foundation is relatively solid. Do you think the final version after polishing more clearly and accurately expresses your original writing intention compared to your original version? In the process of using AI to assist your writing, do you feel you have become more independent or more dependent on AI?

Interviewee : I feel that the first draft I wrote based on my abilities might not have expressed my meaning very well. After his revisions, I am quite satisfied.

Interviewee : I do rely on it to some extent because the tool is quite powerful. It's used in many situations and is very convenient. Even when using it, I still offer my own ideas, such as when giving instructions. Even after it polishes my work, I still add my own thoughts to my essay. So I don't feel completely dependent on it.

Interviewer : You need to be the subject and extract what you need from it.

Interviewee : I don't just accept everything it gives me.

Interviewer: What kind of instructions do you think are more effective during interaction? Interviewee: I feel that I've used several AI software programs, such as Doubao, and actually, the one I use most often is Wenxin Yiyan. I feel that if you give them very precise and detailed instructions, they will give you a satisfactory answer. If it's a bit more general, for example, compared to Doubao over the past few years, it's relatively lower-level. If the instructions you give it aren't particularly precise, it won't react.

Interviewer: I understand. So, do you feel a sense of responsibility for your own writing when using AI for polishing?

Interviewee: Yes. For example, with writing. I remember when it gave me an AI prompt, I forgot if it was from this time, it fabricated something that wasn't in my original essay, and at the time I felt that it wasn't particularly good, and it completely changed what I wrote.

Interviewer : So, do you think you would be willing to use AI as a self-study tool for English writing in the future?

Interviewee: I would be willing, because I feel its functions are very powerful. We actually use it quite a lot in the learning process, and it's very convenient. Mainly, when self-studying, I feel it's a great tool, like the vocabulary memorization method taught by TeacherX.

Interviewer: Do you think it can be used for self-study?

Interviewee : I could give it instructions, take a picture of the words on this page, and have it compile a short essay with a Chinese translation. I feel that would be more convenient for understanding.

Interviewer: You could try this?

Interviewer : In the process of using AI, do you feel that it has inspired you to think more about your writing?

Interviewee: Some of the expressions it provides make me realize my shortcomings. I also carefully consider the essays it polishes and then think about how I should revise them. I feel that it helps me to express my own views.

Interviewer: Do you think the polishing process helps you express your own views in your writing, or does it hinder you from expressing your own views?

Interviewee: Even after it polishes my work, I still need to carefully review it myself. The parts it doesn't approve of actually make me think more deeply.

Interviewer: In your view, what is writing? It's not just English writing.

Interviewee: In my view, writing is more like recording the process of inspiration!

Interviewer : Do you feel that your understanding of writing itself has changed when using AI to assist your writing?

Interviewee : Because whether it's English writing or regular Chinese writing, I feel that inspiration often comes in a fleeting moment, which makes me really want to record it.

Interviewer: You also used it in middle school, and it wasn't just about using it; there was a process of mutual evaluation with your classmates and teachers.

Interviewee: I feel that it hasn't changed. Because no matter how powerful the function is, I feel that to write better, more emotional works, the work still relies more on our subjective consciousness. It's just a tool, at most it can be as a spell checker..

Interviewer : For example, what if you gave your essay to your deskmate, and you two reviewed each other's work? Do you think AI would be a perfect peer? If you let it evaluate your work?

Interviewee : I feel that if it's just AI evaluating your work, it involves a personal touch. It might be too official, too professional, lacking in emotional detail. It doesn't feel as real as when your classmates or teachers evaluate each other; it might be too professional.

Interviewer : Where do you think the personal touch is reflected?

Interviewee : The personal touch, like when teachers and classmates evaluate each other's essays, allows them to empathize with your writing and provide feedback. I feel that AI is more like offering suggestions for improvement regarding techniques and skills.

Interviewer: I don't have any other questions. Do you have anything to add? Regarding AI and English writing.

Interviewee : I still believe that AI is just a tool; it can help us polish our writing. However, I hope that when using AI, everyone should try to retain their own subjective awareness and not rely on it too much. This way, we can still leave ourselves some room for improve thinking ability.

Interviewer : I think it's very important; you must have critical thinking skills. Thank you very much for participating, and I hope your studies go smoothly.

Interviewee : Thank you, teacher!
