# Supplementary material for: Is Gen AI an editor or a revision tool? A case study of EFL students in AI-assisted revision
Source: PLoS One. 2026 Oct 29;21(10):e0359661. doi: 10.1371/journal.pone.0359661 (PMC13718921; doi:10.1371/journal.pone.0359661)
Supplement: Supplementary file 2 [file pone.0359661.s002.zip › Interview data/6.docx]

Interviewer: Our interview is about exploring the relationship between AI and the development of English learners' writing skills. Our conversation won't be known to other teacher, so please just tell the truth. First, let me know your English score in the college entrance exam.

Interviewee: I scored 80 on my college entrance exam English test.

Interviewer: Do you like English?

Interviewee ): I don't really like English. My English scores have always been quite low.

Interviewer: I understand! So, in your daily life, how often do you use AI?

Interviewer: In what areas?

Interviewee: For example, my sister's baby is about to be born, so I searched for names, and I didn't even ask her what a good name would be. Also? Not when you go home? For example, when traveling from Chaoyang District to Beijing West Railway Station, I would ask Du Bao which subway line is best to take.

Interviewer: How often do you use AI when learning English?

Interviewee: Quite often.

Interviewer: In what areas do you usually use it?

Interviewee: English. If I don't know a new word, I'll ask Douban what it means. What else? Basically, I ask about new words a lot.

Interviewer: Also... how much do you use it in English writing?

Interviewee: I use it for writing too. Basically, if I don't know a sentence, I ask Doubao to explain it. If I can't write a sentence, I ask it to generate one.

Interviewer: So he gave it to you, meaning you had him translate it?

Interviewee: It was mostly translation.

Interviewer): I understand. Could you describe how you used AI to polish your writing in this assignment?

Interviewee: I wrote it myself first. The teacher assigned me to write it myself, then take a picture of it, ask him to polish it, and then revise the polished parts. That's basically how I did it.

Interviewer: Did you look at his work again after you finished polishing it?

Interviewee: After he finished calculating, he gave me his polished work to revise again, changing some of the words.

Interviewer: I noticed that the version generated in the video I saw, the one I submitted to my assignment, is different from the one I actually used.

Interviewee : I changed the final version generated by the AI because...

Interviewer: So why didn't you choose the final version generated by the AI at that point? The final version generated by the AIand the final version you submitted to your assignment are not the same.

Interviewee : It's been a while, I forgot a bit.

Interviewer : Wait a moment, I'll find it for you.

Interviewee : With a card? I forgot, I didn't even know what I wrote.

Interviewer : It's okay, it's okay, I'll find it.

Interviewee: I feel...

Interviewer : It seems like you're talking about something similar. Let me check again. Yours is one, but I remembered it wrong. Have you ever had a situation where you didn't accept something after the AI generated it? Why didn't you accept it?

Interviewer : Yes.

Interviewee : I feel some words are very difficult. The words it generated are quite different from my level. The words it generated were very difficult; I couldn't imagine them being that difficult.

Interviewer): For example, after Teacher Zhang assigned you homework, did he ask you to draw out the words that might be modified and the words he learned? To make a mark. Do you think you can use these phrases or words again in the future? Can you think of them when you write them yourself?

Interviewee : Yes, it's possible. I feel the generated data isn't too difficult.

Interviewer: Of all the feedback it provides, which are the most helpful to you?

Interviewee: Vocabulary!

Interviewer: For example, in terms of grammar, vocabulary, sentence structure, sentence style, or language style, or content, which do you find most helpful? So, in what ways does the vocabulary help?

Interviewee : So, sometimes when I can't think of something, it gives me hints.

Interviewer: I understand. In the process of using AI, are you aware of where you might make mistakes, and do you often make mistakes?

Interviewee: H. In English, yes. I often make mistakes.

Interviewer: Because you see, Teacher Zhang had you submit a first version and a final version, and there were markings in between. At that point, did you realize which aspect of your English writing was weaker?

Interviewee : I feel it's still in the connection between sentences; they don't connect well.

Interviewer): Is it logically?

Interviewee: More or less. I always have trouble connecting sentences.

Interviewer: Then...

Interviewer: Do you think it will help you with your subsequent studies?

Interviewer : How will it help?

Interviewee: Expand my vocabulary and memorize the plot. Like, I can fit it into the sentences myself.

Interviewer: After completing the final draft, that is, after submitting this final version, do you feel that the final version clearly and accurately expresses your original writing intention?

Interviewee: Quite clearly.

Interviewer: In what aspects do you feel it's clearer?

Interviewee: My writing was disjointed; the version it generated for me was more coherent. My writing was too stiff.

Interviewer: Do you feel that in the process of using AI to assist your writing, you became more independent or more dependent on it?

Interviewee: I feel I'm somewhat dependent on it.

Interviewer: In what way? Interviewee News Li Xiaohuan (00:08:25): Because I use Douban a lot, but I end up asking questions about everything.

Interviewer: Do you think it affects your thinking?

Interviewee : A little.

Interviewer: Can you elaborate?

Interviewee: It's like that, I ask questions about everything, I don't even want to think about it myself anymore.

Interviewer: If it provides you with something in English, like words, sentences, or text, it's hard for you to think for yourself anymore, right? I understand. So, in the process of interacting with AI, what kind of input commands will get you the more specific and accurate answers you want? Interviewee News Li Xiaohuan (00:09:Interviewee): I'm a bit assertive, so I told him to generate this and that. If there's a word limit, it has to be within a certain number of words.

Interviewer: Do you feel more responsible for your English writing when using AI? Do you feel you've delegated responsibility to AI?

Interviewee: I give more to AI. In writing, I rely more on AI.

Interviewer : How so? Could you elaborate? In which areas have you relied more on it and given it more responsibility?

Interviewee: For example, writing the main body of an event report. I'm not good at that myself; I'm completely clueless!

HA): But you think its writing is okay.

Interviewee: Anyway, it's better than mine. I couldn't think of such good words.

Interviewer: If you were asked to write it yourself without AI assistance like Doubao, you wouldn't be able to write it at all.

Interviewee : I could definitely write these, but I don't want to write the kind of thing that Douban is more convenient for, where you can just search and copy.

Interviewer: But... do you think your writing ability has improved or decreased after using AI?

Interviewee: Probably about the same?

Interviewer: Think back to, for example, when you take an English exam, it's not a computer-based test. When you're writing an essay, you can't use your phone.

Interviewee: Right!

Interviewer: How do you feel about this compared to the final exams last semester?

Interviewee : About the same.

Interviewer: About the same, okay. So, while using AI, do you feel it inspires you to think more about your writing content or language?

Interviewee: I'll memorize some good sentences, like those related to delicious food.

Interviewer : How is it written?

Interviewer: Besides this task, in the future, do you use AI as a self-study tool for English writing?

Interviewee : Self-study. I don't know.

Interviewer: Do you have any English learning software? Interviewee News Li Xiaohuan (00:12:10): There's a Kimi algorithm.

Interviewer: Even excluding it, isn't it still AI? Do you think AI makes you think more about the content or the language of an article?

Interviewee: So it has both, even without content?

Interviewer: You mean both?

Interviewer: Is there a focus?

Interviewee: More content.

Interviewer: By content, do you mean it generates new things for you?

Interviewee: The content it generates must match the kind of content I want. The word order might not be exactly the same. I care more about the content.

Interviewer: You feel that it helped hinder you from expressing original viewpoints in your writing, that is, your own thoughts?

Interviewee : I gave him instructions. I only used it because its content aligned with my own ideas.

Interviewer : You sent him your ideas through instructions?

Interviewee : Yes!

Interviewer: I understand. Do you feel that your concept of writing itself has changed during the process of AI-assisted writing?

Interviewee : No, not at all.

Interviewer): Writing isn't just English writing; it can refer to writing in general.

Interviewee: I don't feel any change, nothing at all.

Interviewer: Is there anything else you'd like to share more about the relationship between AI and English writing?

Interviewer: Sure. Now, please recall that in your regular classes before AI was used, or in your middle school, there were always teacher evaluations and peer reviews. When writing, you and your deskmate would exchange essays. Now, if AI were your partner, would it be a good partner? How would it evaluate your writing?

Interviewee : Sometimes I'll take a photo and ask it for feedback. It's quite objective; it points out my strengths and weaknesses.

Interviewee: I think that's quite good.

Interviewer: Do you think that's less effective in peer evaluation?

Interviewee : Because I feel that those kinds of people, like those with broad knowledge, can make an evaluation.

Interviewer: I understand. Is there anything else you'd like to add?

Interviewee : Oh, and... what?

Interviewer): Then my interview is over. Thank you, I'm sorry to have taken up your time. I wish you all the best in your studies. Yes, bye!

Interviewee: You're welcome. Where shall I leave?

Interviewee : Bye!
