# Supplementary material for: Is Gen AI an editor or a revision tool? A case study of EFL students in AI-assisted revision
Source: PLoS One. 2026 Oct 29;21(10):e0359661. doi: 10.1371/journal.pone.0359661 (PMC13718921; doi:10.1371/journal.pone.0359661)
Supplement: Supplementary file 2 [file pone.0359661.s002.zip › Interview data/7.docx]

Interviewer: Haha, hello. Sorry to take up a little of your time. Thank you for participating.

Interviewer: Do you like English?

Interviewee: I didn't like it much in high school and college.

Interviewer: Why didn't you like it in college?

Interviewee: One? Back in high school, my English was pretty good, and it was usually one of those things that gave me extra points. But in college, classes became boring, and I just wanted to play on my phone. I didn't do well on my first final exam, and I started to give up. I didn't want to study anymore, and I didn't even register for the CET-4 (College English Test Band 4).

Interviewer: So, how often do you use AI in your daily life?

Interviewee: Pretty often. I probably use it for everything.

Interviewer: Could you be more specific about where you use it most often?

Interviewee: For example, what homework does the teacher assign? If I don't have a solution, I might use AI. Or... including some everyday problems. If I don't know the answer to a question on Baidu, I might use AI to look it up.

Interviewer: So, do you use it a lot in your English learning?

Interviewee: Quite a lot, including some writing assignments in class. Or looking up words, for example, if the teacher asks for a short essay, I just copy what the teacher wrote and give it to them.

Interviewer: For example, how do you use it when writing English essays?

Interviewee: AI generates more advanced features; I might add another one later. A simpler one too.

Interviewer: Do you understand this kind of instruction?

Interviewee: Yes!

Interviewer : Can you describe how you used AI to polish your essay in this writing task? Interviewee: I can't say for sure whether the original text was entirely written by me or aided by AI. For example, there were some sentences in the original text where I couldn't remember certain words. I used AI to input a passage of Chinese and asked if it could translate it, and it did. If there were very long and difficult words, I had to replace them, and then the AIpolished the result. It had high-difficulty vocabulary, and the transitions were very smooth, with long sentences.

Interviewer : So, did you adopt these suggestions?

Interviewee: I adopted them. I think this kind of daily writing makes my articles very sophisticated, and I envision my articles looking like this.

Interviewer: Is this the ideal situation?

Interviewee: Bye! Yes!

Interviewer : Because I saw that the content in the link you sent is different from the final version of the assignment you submitted. I don't know if you noticed this?

Interviewee: I noticed it.

Interviewer: Then why weren't the suggestions from AI adopted?

Interviewee: My previous assignment was in Tips. I found the answer, but now I can't find the original link. So this time, I submitted the assignment just to get it done, and I just asked someone to do it for me from Doubao.

Interviewer : You said you couldn't find the link to your previous essay, so you created a new one to respond to other's request.

Interviewee: No, this is a new one I generated?

Interviewer: I understand. You submitted this version, which is the final version generated by the AI.

Interviewer : No, I'll share the paper version you submitted. Yes.

Interviewee: This is my first draft written by AI.

Interviewer : It's entirely AI-generated.

Interviewee: I also analyzed this myself.

Interviewer: I understand!

Interviewer : Because the links you submitted didn't contain phrases like "significant have emerged between," or the words I highlighted in yellow. Did you choose not to use these? Yes.

Interviewee: The assignment I submitted was generated by AI, and I didn't read the details carefully before submitting it hastily.

Interviewer : I understand. So you generally don't read the feedback provided by AI carefully?

Interviewee: Yes, like this time when I submitted the assignment. I usually read the written assignment very carefully. If I don't understand something, I translate it myself and look it up. But I didn't read the last assignment carefully.

Interviewer: In other assignments, which types of feedback provided by AI have been helpful to you, especially in English writing?

Interviewee: I think I'm very satisfied with the long and complex sentences and advanced vocabulary it provided, because that's something I lack. I can only think of some very simple words, but I want my writing to look sophisticated, so I really need very advanced, long words. The first time it's generated for me, I'll look at it carefully and read it thoroughly, but after 35 days, I might forget it.

Interviewer: Have you looked at it? After it was provided to you, did you study it on your own?

Interviewee: I might completely forget it.

Interviewer: Do you think that the next time you use it, you'll be able to recall using these words when writing in English?

Interviewee: I can think of some words that might exist, but I probably won't remember them in writing because I haven't memorized them.

Interviewer : But when you're reading, you might have some impression. When you use AI editing, can you clearly recognize which aspects you need to improve?

Interviewee: Yes!

Interviewee: You realize that?

Interviewer: For example, in which aspect?

Interviewee : It's about polishing AI articles to make my articles. There are many long and difficult sentences. Long sentences first need to be made coherent, that is, they can't be just subject-verb-object sentences, one after another, and then there has to be that.

Interviewee : Formed sentences.

Interviewer: Anything else?

Interviewee: And then. And then connect them, but I don't know how to connect them, and because my logic isn't very strong, maybe when I write articles, it's like... one sentence after another, there's no logic.

Interviewer : I understand. Do you think that when you finished the final polishing, the final version reflected your original writing intention?

Interviewee: No, I feel it's still me. The one written by the other writer reflected it more. What?

Interviewer : Writing intention? Then why do you feel it didn't reflect it? Did they make any changes?

Interviewee: Because I didn't look at it carefully.

Interviewer : So, do you think that in the process of using AI for English writing, you've become more independent?

Interviewee: Definitely more dependent. I usually start writing at 11, and I can't help but generate the AI.

Interviewer: More dependent on AI?

Interviewer : I understand. So, how do you usually generate the AI?

Interviewee: I would say, "I'll give you the teacher's writing requirements. I'll type the original text directly and tell them what format I need. For example, around 200 words, simple."

Interviewer : After it generates the text, do you think about where it needs to be changed before giving it further instructions?

Interviewee: Yes, I usually do that. There are some sentences I'm not satisfied with. For example, the sentences generated by Doubao are relatively simple, while "deep thick" is more difficult. For example, "deep sick," I'll say, "Make it simpler," and then Doubao will add a translation. Doubao might then make it even more complex.

Interviewee: For example, I might regenerate it, or make it generate more words.

Interviewer: Do you feel that your English writing ability has improved during this process? Interviewee: Overall, no. I think my writing skills are nowhere near as good as they were in high school. For example, in exams now, is the writing section just the exact text from the book? For the last sentence, I usually just want to memorize it and copy it directly without adding my own thoughts. I'm too lazy to think now.

Interviewer: But in high school, you were willing to think.

Interviewee: Yes!

Interviewer: So why do you think you need to think when writing?

Interviewee: I think writing is definitely subjective. Everyone might have different opinions. Even with the title of an essay, everyone might have different interpretations. I might really want to write down what I'm thinking, but I don't know how. I might use AI.

Interviewer: Do you feel that your concept of writing is different now that you've used AI-assisted English writing?

Interviewee: Not really!

Interviewer : In what ways is it different?

Interviewee: In high school, writing was more of a subjective choice; I wanted to write. But in university, writing became a task. Without a task, I don't even think about English writing. Even for exams like the CET-4/6, where there are writing assignments, it's only because I want to pass. If there's no exam to pass, I don't even think about writing practice; it's not that important.

Interviewer: Do you think English writing is important for your future development?

Interviewee: I'm not currently working in an English-related field, and I don't plan to develop a career in English in the future.

Interviewer : So you think it's fine to just use AI to generate it, since you don't want to waste too much time, right?

Interviewee: Yes!

Interviewer : I understand. In the interaction between AIs, what kind of instructions do you give them so that they can more accurately understand what you need?

Interviewee: First of all, sometimes AI doesn't understand human language, and the generated output is completely different from what I expected. I have to tell it what I want. Simple and easy to understand. Yes, simple and easy to understand.

Interviewer: Let me ask you another question. Do you feel more responsible for your work when using AI to polish it? Do you mean you've handed over the responsibility to the AI?

Interviewee: Mainly, I've handed it over to the AI, right?

Interviewer: You don't seem to care much about the final grade because it's written by AI.

Interviewee: I do care about the grade, mainly because of last time. My handwriting was very messy and perfunctory in my assignments. I didn't expect it to teach me anything, and last year I got a very low participation grade. This year I knew I couldn't do that again, so I wrote my handwriting much better.

Interviewer : Good!

Interviewee: Very good. I read some parts myself and worked very hard, but the article was indeed written by AI; I couldn't write it myself.

Interviewer : Okay! What? I understand!

Interviewer : I understand. Do you think that in the process of using AI, it helps you improve your own viewpoints or hinders you from expressing your own opinions?

Interviewee: Does it help me express my views? If some of it doesn't meet my expectations, will I let it continue generating, or will I retain my own views? What does it mean to have it translate from Chinese to English for me?

Interviewer: In what ways are your own views generally retained?

Interviewer : You just said you have your own views? What kind of views does the AI express that you haven't accepted?

Interviewee: It's still that subjective opinion. Everyone has their own perspective on the content of an article, and I'll retain mine. I won't adopt the AI's perspective, and I might even have it replace its own viewpoint. I'll say, "Change a certain paragraph to something else," and that's my opinion.

Interviewer: So, what do you think? In high school or university, you probably had peer review sessions. You'd hand your assignments to your deskmate, and you'd critique each other. Do you think that when using AI in university, it's a perfect peer?

Interviewee: I think it's a better peer than classmates. In university, when classmates exchange papers, it seems like no one has the patience to read them carefully. And if the teacher gives an instruction to exchange papers, I might secretly look at my phone instead of reading the other person's paper, because I know it's probably written by AI. Then in high school, nobody had cell phones, so everyone usually wrote their own stories.

Interviewer : Right, why do you feel you don't want others to see your wildly imaginative writing?

Interviewee: But high school also has its limitations. Because everyone writes their own stories, you don't really want others to see those kinds of sequels, those kinds of wildly imaginative stories.

Interviewee: Is it even possible to write a sequel? Everyone has their own ideas. Maybe I didn't understand it, but I still forced myself to write it. Sometimes I understood it, and I made up a really outrageous story. If others saw it, they would say, "What did you write? It's not what you meant at all!" And then I would feel a little embarrassed, so I would only read it myself, or only show it to the teacher.

Interviewer: So what do you think? Is your classmate's behavior okay?

Interviewee: It's not very good. I do it too, everyone does it, but it's probably not good for teachers, and they don't have enough patience to read the article.

Interviewer: It's not like what you just mentioned in high school. When others see your continuation, they think it's outrageous, what is this? You feel embarrassed. Do you think their behavior is right?

Interviewee: Actually, I think it's okay, it's just that it's written incorrectly, definitely incorrectly. And when they correct it, it's probably just a joke between classmates. People who aren't very close wouldn't say what you wrote, it's definitely someone very close who would say that.

Interviewer: In your concept, what is your concept of the word "writing"? Is it just English writing?

Interviewee: Writing, including my current studies in journalism, involves writing. I feel that when writing subjective things, you have to incorporate your own thoughts and express genuine feelings.

Interviewer : Do you think English writing has changed your understanding of this concept? Why not?

Interviewee: I mainly write in both Chinese and English. For example, during the college entrance exam, I specifically practiced writing in English to improve my English score. In high school, English writing was also assigned as homework by the teacher. When you write normally, you definitely wouldn't suddenly write an English article; it's usually more in Chinese, at least for me. I want to share this.

Interviewer: In your eyes, English writing is just for exams and for scores. I understand. Is there anything else you want to share about the relationship between AI and English writing? Feel free to add anything you want to say. Anything that sparks your thinking and that you want to share with others.

Interviewee: No, for example, this time it was homework assigned by my English teacher. I feel it wasn't really necessary. Actually, it wasn't even my original article. It wasn't entirely half of what I wrote myself; it was AI writing, letting AI write its own article. I still had to handwrite everything and then organize it. For the first five minutes, I had a relatively strong impression, but after that short time, I probably forgot everything. I think this kind of homework is unnecessary.

Interviewer : So what do you think? At the beginning, you just said it was written by AI, and then you let AI polish it. How did you initially let AI write it?

Interviewee: At the beginning, I might have signed it. I definitely had some things I wrote myself. If there were parts I didn't know, I would ask the AIto translate the sentence into Chinese, and then it would translate it for me. I just copied it down. This is a kind of logical demonstration; I suddenly thought of a sentence, I asked it to translate, and I wrote it down. Obviously, if I think of another sentence, I translate it, and then I ask it to write it down again, the logic isn't very strong. After I organized the whole article, I asked it to be polished, and then I added a translation. But even the polished version wasn't satisfactory; it might be too long or too difficult. I suggested something simpler, and then I copied down the generated version.

Interviewer : I understand, from that perspective. In my eyes, it probably won't improve you. For example, if you were asked to write in English without your phone during an exam, wouldn't you feel your writing ability is weaker than in high school?

Interviewee : Yes!

Interviewer: I understand. So, what do you think we should do in the future to give you more motivation or better methods to learn from AI and English writing? Interviewee(00:24:07): A better way to learn. I think we need to break away from AI, completely break away from AI, and then you write it yourself. No matter how it turns out, it's your own writing. Then, with teacher guidance, I think it's definitely better than AI.

Interviewer: What aspects do you think teachers should focus on to help you absorb more?

Interviewee: If you write it yourself, you'll definitely write very simple sentences. Then the teacher can expand on those sentences, teach more complex vocabulary, or correct grammar, etc.

Interviewer: And then let you practice on your own in class, without using your phones. Wouldn't that be better?

Interviewee: It would definitely be better, but I just don't really want to write anymore?

Interviewer: I understand. Okay, is there anything else you'd like to add? That concludes my interview. Thank you, and goodbye!

Interviewee: Okay, okay, thank you, teacher. Goodbye!
