# Supplementary material for: Is Gen AI an editor or a revision tool? A case study of EFL students in AI-assisted revision
Source: PLoS One. 2026 Oct 29;21(10):e0359661. doi: 10.1371/journal.pone.0359661 (PMC13718921; doi:10.1371/journal.pone.0359661)
Supplement: Supplementary file 2 [file pone.0359661.s002.zip › Interview data/8.docx]

Interviewer: Sorry to take up a little of your time.

Interviewee : Okay!

Interviewer: Are you at school now?

Interviewee : I'm at school!

Interviewer : Okay, so I wanted to understand some of the relationships between AI and English learning/writing. Some exploration, and I'd like to understand your thoughts. Our conversation won't be known to anyone, so just tell me the truth.

Interviewer: How often do you use AI in your daily life? In what areas do you use it most often?

Interviewee : I used to ask Baidu when I didn't know the answer to a question. Now, with more AI, I feel its answers are more targeted. I don't need to search through posts anymore, so I usually ask AI whenever I have a question.

Interviewer: How often do you use it in your English learning process?

Interviewee : More often for knowledge points I don't understand, and I ask it for homework assignments or to write an example so I can look at it myself.

Interviewer: Also a lot in writing? Have you ever had any activities where you were asked to use AI to polish your writing?

Interviewee: Yes, I think so.

Interviewer: How do you use it for polishing? What instructions do you give it? Interviewee : Use some adjectives and adverbs to make the sentences better.

Interviewer: Which of the feedback he gave you was the most helpful?

Interviewee : Position.

Interviewer: Because after you give him some instructions, he returns a version, right? The version he returns will definitely be different from your initial version. You'll check it again. So, what do you think is most helpful at this point, for example, grammar, vocabulary, content, or sentence structure?

Interviewee: Maybe better coherence, the connection between sentences.

Interviewer: You mean the logic is correct?

Interviewee: Sometimes what he gives just doesn't look right, and sometimes I don't use it.

Interviewer: So, in the process of using AI, do you think you will completely accept its suggestions? Sometimes do you reject them?

Interviewer: Why do you feel it's not quite right? Why doesn't it meet your requirements?

Interviewee: Maybe... before. Before, when I was writing that literature review, I searched online, and I didn't give it an example. Then it gave me one that was completely different from the example the teacher gave me, and I... felt something was wrong.

Interviewer: So, after reading it, do you think you can realize in which areas you are more likely to make mistakes? Hello, can you hear me?

Interviewee : I can hear you thinking.

Interviewer: Okay!

Interviewee : I haven't used it too much. I use it occasionally, but not a lot. In various aspects.

Interviewer : After you've seen his revisions, do you feel he's captured your initial writing intent? Do you feel that using AI to assist your writing has made you more independent or more reliant on it?

Interviewee : Reliant on it means that if I think the instructions he generated this time are good, I'll just give him those instructions next time and revise them myself. I might not think for myself first; I'll just let him write. When the AI highlights a different wording, I compare it with my sentence and revise it straight away if I think it works better.

Interviewer: For example, in what aspects do you rely on it?

Interviewer: Do you stop thinking for yourself, for example, how to structure the essay, what content you want to write?

Interviewee: I just give him some general instructions and let him write it. I check it, give him a quick look.

Interviewer : So, do you feel that you've become more responsible for your English learning or writing? Have you delegated responsibility to AI?

Interviewee: For assignments, it might reduce the sense of responsibility, but it's normal for exams.

Interviewer: Will you continue to use AI as a self-learning tool in the future?

Interviewer: In which areas will you use it?

Interviewee: In areas I'm not very familiar with.

Interviewer: I didn't quite catch what you meant.

Interviewee: In areas I'm not very familiar with, areas I don't understand!

Interviewer: What do you think? Which areas are you not very familiar with?

Interviewee: It's those technical terms and things that cause rejection. Sometimes it might be because the specified numbers aren't clear, so the result it gives doesn't meet my expectations.

Interviewer: So what do you think? Did you fully accept his advice? Did you reject any suggestions? What were your reasons for rejecting them?

Interviewee: It wasn't useful.

Interviewer: In what specific aspects did you feel it didn't meet your expectations?

Interviewee: For example, I gave him a document, and I wanted to find something in it. His example was based on that example, sometimes he might use online searches or something, combining many cases, and then giving me a range that's different from what I initially entered.

Interviewer: I understand. So, you're asking about your own perspective on writing, not just English writing, but also fun writing.

Interviewee: I think it probably still comes down to personal feeling. I feel like the person writing it has to write it themselves. Because the words don't seem to have any emotion.

Interviewer: So, after using AI to assist your writing, do you feel your views on writing have changed?

Interviewee : I might be more inclined towards human-written content.

Interviewer : Think about it again. In university or high school, you've definitely had peers or teachers evaluate your work. For example, you might exchange assignments for feedback. From this perspective, if there's no physical peer evaluation in the future, do you think AI will be a perfect form of peer evaluation?

Interviewee: Maybe. I always feel it might be too perfect.

Interviewer : Too perfect? What do you mean?

Interviewee : It might be... too perfect. Peers might have more... their own... their evaluation might be more accurate and comprehensive than a peer's evaluation, but peers... They might evaluate them based on their age. Evaluation!

Interviewer : So, is there anything you'd like to add regarding AI and English writing?

Interviewee: No, teacher.

Interviewer: Okay, then there are no other questions.
