# Supplementary material for: Is Gen AI an editor or a revision tool? A case study of EFL students in AI-assisted revision
Source: PLoS One. 2026 Oct 29;21(10):e0359661. doi: 10.1371/journal.pone.0359661 (PMC13718921; doi:10.1371/journal.pone.0359661)
Supplement: Supplementary file 2 [file pone.0359661.s002.zip › Interview data/9.docx]

Interviewee: Hello teacher, do I need to turn on the video?

Interviewer: No need to turn on the video, this is fine. Are you at school?

Interviewee: Okay, my teammates are in the humanities building.

Interviewer: That's quite high. How often do you use AI?

Interviewer: I should add that Xiaoyu won't know the content of our interview. So, how often do you use AI?

Interviewee: Yes, and you tell me?

Interviewer: Where do you usually use it?

Interviewee: I usually use it for less important assignments. For more important course assignments, when I don't know the answer, I can ask the AI. Based on its answer, I might adjust and reflect on it. Sometimes, for things I think are important, I won't completely copy it; I just let its answer provide a perspective. Based on its answer, I'll refine it further, and maybe... When participating in certain activities, if I'm asked to write a report, I might feel it's not very important, so I'll have AI write one for me.

Interviewer: Do you do a lot of English writing?

Interviewee: I do a lot of English writing. I had writing courses in my first and second years of university. In the first semester, there was a final exam, so I didn't write much myself at first. Later, I found it too slow, and the teacher suggested I collect materials online and start using them gradually. I also used many other writing courses later, and after finishing, I would adjust it based on the content because the generated document often had many problems, for example, the sentences were too long, and one sentence was too long and I had to break it down and change words myself.

Interviewer : Have you ever used AI to polish your articles? After you finish a draft, do you send it to them?

Interviewee: Yes, including now after I write my thesis proposal, when I input the literature review. Sometimes, after he generates a dimension for improving students' cultural awareness based on the curriculum standards, he lacks something. I revise it based on his writing, and I find that the logic isn't very coherent. I then send it back to him, telling him which two or more aspects, and which sentences should have transitional language added, so he can polish it for me. That's been going on recently.

Interviewer: So, which of his feedbacks do you find helpful? And what exactly did you mean by "need content"?

Interviewee: It's helpful. The helpful part is that his answers always have main headings and subheadings, making it very clear what he's talking about. Then, based on this framework, we integrate the content, because we don't actually need the framework itself. We just need the content. With this framework, we can clearly understand which aspect it's talking about, which I think is very useful.

Interviewee: For example, if I had a feasibility analysis today or yesterday, I would tell him all the literature I had read. Then I would ask him to generate a summary based on these documents, extracting the mainstream theories of reading instruction from both China and abroad. It would then generate some content for me. Whether the generated points are useful depends on whether they are what I want. Sometimes what it generates isn't what I want; there will be deviations. I will promptly send him more instructions to make him understand. Sometimes I feel he doesn't understand.

Interviewer : What kind of instructions do you think make him understand you better?

Interviewee: This is how I do it. If he doesn't quite understand after one or two times, I will continue to tell him what additional conditions to add based on the above information. I will continue to add and explain what I need. I have a little trick to make him understand: after telling him the requirements, I give him an example. I then strictly follow the example based on the requirements. My rule is, for example, to have him convert foreign names into abbreviated forms for academic documents. Because with some names, I don't know the country of origin. I don't know which part is the surname; some are very long, like "Maria." So I just copy the instructions, the requirements for abbreviating names from the academic level, and the examples, and then ask him to provide those requirements and examples.

Interviewee : These names. Converting them into abbreviated forms for academic documents, he'll understand very well. I'll tell him that a normal, correct requirement will include a small example, and he'll generate the abbreviated form based on that example, which will be very accurate.

Interviewer: I understand. After using AI for polishing, do you feel you clearly realize which aspects you need to improve?

Interviewee : I think the most important area for improvement is the lack of a broader perspective. I don't know how to write; that's the biggest problem—a lack of ideas. You see, if we know the direction of our thinking, at least we can write something. The biggest problem is not knowing where to start. Interviewer: After he gives you some ideas, are you inspired and know roughly which aspect to write about?

Interviewee: Yes, I ask him to give me a perspective and examples, mostly like that. This includes writing papers, because recently I've been writing literature reviews and such.However, even though it was accurate, it didn’ t fit my writing.

Interviewer: In your classes, for example, there are writing classes or essay writing classes, have you ever used AI to assist in writing?

Interviewee : Thank you. Yes! Yes! We all do, including the teachers? We ourselves?

Interviewer: Yes, anything is fine, basically any assignment the teacher gives you.

Interviewee: Anything is fine. There were also teachers who didn't assign him any enriching tasks. You do have I Smart, which we use for writing. We use it for electronic writing, but we don't use AI to help us. I mostly use AI ourselves, including for advanced English and other writing courses.

Interviewer: Do you think it has improved your language skills?

Interviewee: No, I don't think so, haha.

Interviewer: You think the vocabulary it provides is bad, and you didn't accept it?

Interviewee : The vocabulary is quite good, sometimes it's quite difficult, sometimes I don't understand it. But for me, I think language ability can't just be judged by the difficulty of the vocabulary. I think it's more important to look at the beauty of the language, the quality of its rhetoric, which better reflects the richness of the language, as well as the sentence structure. Just looking at the vocabulary might not be enough to assess the language proficiency.

Interviewer: So, do you think the final version after using it for polishing accurately reflects your initial writing intention?

Interviewee: Yes, it does. It went through 11 iterations, showing how it generates what you want. It can't say it covers everything, but it definitely borrows some elements.

Interviewer: Do you feel that in the process of using AI-assisted writing, you've become more independent or more reliant on AI?

Interviewee : I think I've become more reliant.

Interviewer: Why?

Interviewee: Because knowing there are convenient conditions, I felt that since I had to complete the task anyway... There's no need to go through the same rigorous process as in high school, racking your brains and painstakingly typing out each word. Just write a rough outline, and you can correct it yourself. This approach makes you more reliant on the current system, as you're content with just getting your usual grades. You won't put in the extra effort to write everything yourself, because you're busy with other things besides studying for exams, and you can't avoid spending more time on this assignment.

Interviewer : So you think your future career path has nothing to do with English?

Interviewee: Yes. I'm currently preparing for the civil service exam, so if I pass, I won't become a teacher. If I don't pass, I'll take the teacher recruitment exam and become a teacher.

Interviewer: I understand. For you, right now you feel that as long as I complete this task, that's enough. Do you feel more responsible for your English learning or writing during the process of interacting with AI and having it polish your work?

Interviewee: Yes.

Interviewer : You've handed the responsibility over to AI?

Interviewee: I've handed the responsibility over to AI, yes, that's right, it has nothing to do with me.

Interviewer: So if you get this score in the end, whether it's high or low, you feel it doesn't really matter much to you? As long as you pass, that's fine, what do you think?

Interviewee : That's right!

Han: How did you originally view writing? Did you only mean general writing, not just English writing?

Interviewee: It's not just English writing. All writing is used to express our views and thoughts. At the same time, it can highlight the depth of a person's thinking and their language proficiency. Writing is also used for assessment; you see it in exams to test everyone's language skills. All language courses, including Chinese and English, include it. So writing can highlight a person's comprehensive language proficiency because it's not just about word choice, but also logic and the writing intention, even if it's not clear.

Interviewer: After AI appeared, and you started using AI to assist your writing, has your understanding of writing itself changed?

Interviewee: No, I still think it reflects a person's comprehensive language proficiency.

Interviewer : So, since you had peer review sessions in high school or university, and didn't use AI, maybe you'd review each other's essays or something, do you think AI would be a perfect peer to evaluate your writing?

Interviewee: Yes.

Interviewee: It can't be called perfect. I think it's still a program and can't completely replace human evaluation. Because writing is a very subjective thing, very subjective. For example, the other day, some students were preparing for their TEM-4 exams, and some even had AI grade their essays. The result was that when Doubao, Kimi, or Deep Thick were given the same scoring criteria, the scores they gave were completely different, even very different. From my perspective, I thought it was very well written and logically sound. In this TEM-4 (Test for English Majors-Band 4), if the score is 20, I think I could get around 13 or 14 points. But the AI generated single-digit scores, which I think are too low, like 7 or 8. Then there are some AIs that I think are quite high, giving me 18 points, which is still quite different. It can't completely replace a human; after all, it's a program.

Interviewer: Have you thought about why you got 7 or 10 points?

Interviewee: It might be due to different programs. Based on the requirements, the AI might focus more on two aspects of the requirements. It might emphasize those two or a few aspects, and if the student writes very well in those areas, then the score will be very high or very low.

Interviewer: So you think that if it were a peer or teacher evaluating you, they would have a specific scale, and you would know where your score came from, right?

Interviewee : Yes! Yes!

Interviewer: So you'd be more inclined to trust the scores given by teachers or peers based on the scale.

Interviewee: Yes, I think AI isn't very accurate, because it's ultimately a rational machine.

Interviewer: Okay!

Interviewee : I think writing is a very emotional and subjective process. This is about the relationship between AI and writing.

Interviewer: Do you have anything to add regarding the relationship between AI and writing? You can share some of your previous thoughts.

Interviewee: I still think that if you have time, everyone should write more on their own. Don't rely too much on it. I've found that relying too much on AI can significantly reduce the depth of your own thinking and thought processes, and you'll completely get stuck in the current situation. I'd have the AI generate a scale for me and then look at it. When I was a freshman or sophomore and had more time, I would still choose it. I did quite a lot of writing independently back then, for example, when it was still quite traditional, using pen and paper. I really liked using pen and paper instead of typing; I preferred to write by hand. I think it would be better to write it myself first and then have AI generate a copy so I could compare and use it as a reference. Or, I could send my essay to them and see their evaluation and thoughts, how they could improve it based on my writing. This would be much more helpful for my writing skills than if it were generated directly and I revised it myself. Because once it's generated, it's basically a whole piece, and we can only make minor changes. So, it's better to write it myself first and then have AI evaluate and provide feedback. Even if our writing is terrible, it can gradually give some suggestions for improvement, and at least we can understand where we can work harder.

Interviewer: Then I have no other questions.

Interviewee: Okay, teacher, it's alright, thank you, teacher! It's nothing!

Interviewer: Thank you, and I hope you pass the civil service exam! Bye!
